# Evolutionarily divergent spliceosomal snRNAs and a conserved non-coding RNA processing motif in *Giardia lamblia*

**DOI:** 10.1093/nar/gks887

**Published:** 2012-09-27

**Authors:** Andrew J. Hudson, Ashley N. Moore, David Elniski, Joella Joseph, Janet Yee, Anthony G. Russell

**Affiliations:** ^1^Alberta RNA Research and Training Institute, ^2^Department of Biological Sciences, University of Lethbridge, Lethbridge, Alberta T1K 3M4 and ^3^Departments of Biology and Chemistry, Biochemistry Program, Trent University, Peterborough, Ontario K9J 7B8, Canada

## Abstract

Non-coding RNAs (ncRNAs) have diverse essential biological functions in all organisms, and in eukaryotes, two such classes of ncRNAs are the small nucleolar (sno) and small nuclear (sn) RNAs. In this study, we have identified and characterized a collection of sno and snRNAs in *Giardia lamblia*, by exploiting our discovery of a conserved 12 nt RNA processing sequence motif found in the 3′ end regions of a large number of *G. lamblia* ncRNA genes. RNA end mapping and other experiments indicate the motif serves to mediate ncRNA 3′ end formation from mono- and di-cistronic RNA precursor transcripts. Remarkably, we find the motif is also utilized in the processing pathway of all four previously identified *trans*-spliced *G. lamblia* introns, revealing a common RNA processing pathway for ncRNAs and *trans*-spliced introns in this organism. Motif sequence conservation then allowed for the bioinformatic and experimental identification of additional *G. lamblia* ncRNAs, including new U1 and U6 spliceosomal snRNA candidates. The U6 snRNA candidate was then used as a tool to identity novel U2 and U4 snRNAs, based on predicted phylogenetically conserved snRNA–snRNA base-pairing interactions, from a set of previously identified *G. lamblia* ncRNAs without assigned function. The *Giardia* snRNAs retain the core features of spliceosomal snRNAs but are sufficiently evolutionarily divergent to explain the difficulties in their identification. Most intriguingly, all of these snRNAs show structural features diagnostic of U2-dependent/major and U12-dependent/minor spliceosomal snRNAs.

## INTRODUCTION

Eukaryotic precursor (pre-)RNA processing often requires ribonucleoprotein (RNP) complexes consisting of conserved and essential non-coding (nc)RNAs. Notable examples are the small nucleolar (sno) RNPs that participate in eukaryotic ribosome biogenesis through structural modification of specific nucleotides in ribosomal RNA (rRNA) and/or targeting cleavage of the pre-rRNA [reviewed in (1–3)]. Another prevalent eukaryotic RNA processing event is mRNA splicing—the removal of intervening intron sequences from pre-mRNAs that is catalysed by the dynamic RNP complex termed the spliceosome [reviewed in (4)]. The vast majority of spliceosomal introns are classified as major-type (U2-type) and are removed by the major (U2-dependent) spliceosome. The U2-dependent spliceosome consists of five evolutionarily conserved small nuclear (sn) RNAs, U1, U2, U4, U5 and U6, and potentially hundreds of associated proteins [reviewed in (5,6)]. Spliceosome-mediated intron recognition and excision requires intricate base-pairing interactions between the snRNAs and conserved intron boundary and internal branch-point sequences and numerous snRNA–snRNA intermolecular base pairings, dynamically changing during the splicing cycle (7).

Although nearly all examined eukaryotic genomes seem to contain major spliceosomal introns, a much smaller subset of eukaryotic organisms also possess a rare class of minor (U12-type) spliceosomal introns, which are excised by a distinct minor (U12-dependent) spliceosome [reviewed in (8,9)]. The U12-dependent spliceosome contains a unique set of snRNAs, U11, U12, U4atac, U6atac that are functionally analogous to the U1, U2, U4 and U6 snRNAs, respectively, but shares the U5 snRNA also found in the U2-dependent spliceosome. Features shared between the U2-dependent and U12-dependent spliceosomes, including U5 and some common core protein constituents, and secondary structural similarities of the snRNAs, may indicate a common ancestral origin for both spliceosomes (8). The evolutionarily distant relationship of the limited number of species known to possess a U12-dependent minor spliceosome indicates its early origin in eukaryotes (10–12). Based on detailed analysis of ancient intron insertion sites, Basu *et al.* 2008 (13), proposed that major U2-type introns pre-dated the existence of U12-type minor spliceosomal introns, and the observation that all organisms containing a U12-dependent spliceosome also have a U2-type major spliceosome is not inconsistent with this idea. Furthermore, it is hypothesized that spliceosomal introns and components of the spliceosomal machinery are derived from group II introns, based on observation of regions of similar snRNA and intron structure, and splicing reaction mechanism (14–17). Identification of any extant organisms possessing splicing systems and introns with features characteristic of transition stages in such evolutionary pathways would help to further evaluate these models for intron evolution.

The diplomonad protist *Giardia lamblia* is a prevalent human enteric parasite that displays a highly reduced compact genome and somewhat limited metabolic capacity (18). To date, only nine spliceosomal introns have been identified in *G. lamblia*, and they exhibit extended highly conserved 5′ splice sites, and atypical fused branch point and 3′ splice sites (18–21). Our group (21) and others (22,23) identified several cases of *trans*-splicing of these *Giardia* spliceosomal introns (four of the nine characterized introns). In this *trans*-splicing pathway, exons dispersed to distant regions of the genome are expressed as distinct pre-mRNA transcripts that somehow associate to mediate exon ligation. Determining the mechanistic details of how this occurs will require identification and characterization of *Giardia* spliceosomal components and potentially other required RNA processing complexes. Association of individually transcribed exon–intron containing pre-mRNA precursors is predicted to occur through base-pairing potential evident in respective introns halves (21,23) somehow positioning intron splice sites for recognition by the *Giardia* spliceosome.

Identification of spliceosomal introns and putative core spliceosomal proteins in *G. lamblia* (19) strongly argues for the existence of a functional spliceosome in this organism. The *Giardia* spliceosomal snRNAs have been elusive and recently putative *G. lamblia* U1, U2, U4 and U6 spliceosomal snRNA candidates were predicted computationally by examining the *Giardia* WB strain, the only one for which extensive genomic DNA sequence information was available at that time (24). These candidates were structurally divergent, and our recent search for orthologues of these snRNA candidates in the genomic sequences now available from the two related *G. lamblia* isolates (see later in the text) reveals extensive unexpected sequence variation, including nucleotide substitutions disrupting critical and strictly evolutionarily conserved sequence motifs and secondary structures that are fundamental to spliceosome function in other eukaryotes.

In our study, we have taken advantage of the genomic sequence information available for three different *Giardia* isolates (strains), non-coding RNA sequence information obtained from previously constructed cDNA libraries and our discovery of a conserved RNA processing motif, to identify and characterize new *Giardia* non-coding RNAs (ncRNAs). This includes the identification of a new set of spliceosomal snRNAs that show strict conservation of functionally important sequence elements in all three isolates and compensatory mutations maintaining predicted secondary structures.

## MATERIALS AND METHODS

### RNA motif identification and characterization

Genomic regions encoding biochemically isolated *G. lamblia* WB isolate ncRNAs (25–28) were identified by BLASTN searches using the GiardiaDB website (www.giardiadb.org). For each region, 300 nt of additional upstream and downstream flanking genomic sequence were then analysed by manual inspection for any conserved sequence elements evident when aligning the collection of genomic regions. This analysis revealed the presence of a conserved 12 nt motif residing adjacent to or overlapping genomic regions encoding documented (or predicted) mature ncRNA 3′ ends. Homologous ncRNA-encoding regions in the *Giardia* GS and P15 isolate genome sequences were identified using the WB sequences as BLASTN queries. After aligning all sequences using ClustalW (29) to identify all motif sequences in ncRNA genes and those also found in *trans*-spliced intronic regions, a motif consensus frequency plot was generated using WebLogo software (30). Secondary structures for ncRNAs plus their downstream motifs were predicted using Mfold (31) or RNAalifold software (32).

### Identification of new *Giardia* ncRNAs

Our search strategy for new ncRNAs exploited several emergent properties of *Giardia* ncRNA genes: (i) they are usually located in intergenic regions between open reading frames (ORFs); (ii) the conserved RNA sequence motif is located in their 3′ downstream flanking region; and (iii) many *Giardia* ncRNA sequences are preceded by A-T rich genomic sequence elements that are predicted initiation sites (25) based on similarity to the transcription initiation sites of *G. lamblia* protein-coding genes (33,34). Initially, we performed simple BLASTN searches using the more common individual RNA motif sequence variants as queries against the WB genome and searched for instances that also contained upstream A-T rich sequences. This analysis unexpectedly identified motif sequences in the downstream regions of all four previously characterized *Giardia trans*-spliced intron 5′ halves (the 3′ ends of which were previously unknown) and also motif sequences within four putative ORFs, but this method was not efficient for identifying new ncRNAs. Next, we utilized the pattern matching program ‘scan for matches’ (35). Whole genome sequences for *G. lamblia* WB, P15 and GS isolates (provided at GiardiaDB.org) were used as local databases in ‘scan for matches’ searches using the following parameters: AAAAAAAAAA (allowing five mismatches)… 1–500 nt … CCTTYNHTHAA, where ‘Y’ is a pyrimidine, ‘H’ is A, C or T nucleotide and ‘N’ is any nucleotide. This scan yielded ∼400 matches in each *G. lamblia* isolate, which were further screened using pairwise BLASTN comparisons of each *G. lamblia* WB match against the matches from the P15 and GS genomes. Only instances where the promoter and motif sequence elements were present in corresponding genomic regions in all *Giardia* isolates, and the matches also mapped to intergenic regions, were deemed probable ncRNA candidates and were further considered. The candidates were then inspected for other hallmark sequence elements (e.g. conserved box C/D and H/ACA sequences for snoRNAs; conserved snRNA elements, such as the 5′ splice site binding sequence for U1, ACAGAGA sequence for U6) and overall secondary structures (Mfold and RNAalifold) to classify their function. This strategy identified the GlsR26 and GlsR27 box H/ACA snoRNAs, the GlsR28 ncRNA of unknown function and the new U1 and U6 snRNA candidates.

The novel *G. lamblia* U6 snRNA candidate then permitted prediction of new U2 and U4 snRNA candidates through its evolutionarily conserved ability to form extensive intermolecular base pairs with these other snRNAs. Many previously identified *Giardia* ncRNAs have no assigned function (27); therefore, we reasoned that some of these may be snRNA homologues. From these, we generated a concatenated sequence file appropriate to serve as a library for our searches.

To identify the U4 snRNA, *Giardia* U6 nucleotides C21 to U60 are those predicted to be involved in U6/U4 snRNA base pairing and were, therefore, used as query in BLASTN searches of the concatenated ncRNA file, increasing expect thresholds to 10^4^ to optimize search sensitivity for short sequences. This revealed extensive complementarity between the *G. lamblia* U6 snRNA and ncRNA ‘Candidate’-11 (27), implicating it as a potential U4 snRNA candidate. Further Mfold analysis and manual sequence inspection showed that Candidate-11 could form conserved intermolecular helices I and II with the U6 snRNA candidate and also a canonical U4 snRNA 5′ stem-loop (SL). These findings, in combination with compensatory mutations in *Giardia* GS isolate intermolecular helix II (Figure 4B) that maintain U6/U4 snRNA base pairing provided the evidence that Candidate-11 is the *G. lamblia* U4 snRNA.

Identification of a U2 snRNA candidate is more challenging because of the short and discontinuous base pairing between U2 and U6 snRNAs. Here, we used the Spin program (Staden freeware package, 1996) to search the concatenated library for any ncRNA that had the ability to form base pairs with the extended branch-point sequence found in *G. lamblia* introns (AACTAACAC, branch point ‘A’ underlined). This search revealed that uncharacterized ncRNA Candidate-14 (27) contains nucleotides ‘_26_GUGUAGUU_33_’ that are able to form extensive base pairs with the intron branch point with ‘bulged’ adenosine nucleotide configuration. Further analysis revealed that Candidate-14 could form canonical intermolecular helices I through III with the U6 snRNA, with regions of pairing occurring at the same relative positions as other representative eukaryotic U2/U6 snRNA complexes. Mfold analysis predicted U2-like helices IIa, III and IV in the 3′ half of Candidate-14, further implicating it as the *Giardia* U2 snRNA.

### Reverse transcriptase-polymerase chain reaction, primer extension, random amplification of cDNA ends and northern blot experiments

*G. lamblia* WB strain (clone 6, ATCC 30957) axenic trophozoites were cultured in modified TYI-S-33 medium, and *Giardia* genomic DNA and total RNA were extracted using DNeasy Kits (Qiagen) and TRIZOL Reagent (Invitrogen), respectively, according to the manufacturer’s instructions. Reaction conditions used for reverse transcriptase (RT) and polymerase chain reaction (PCR) experiments on *Giardia* nucleic acid samples were performed as previously described (20), using oligonucleotide primers in Supplementary Figure S13.

Cellular expression of candidate ncRNAs was verified by northern blot (snRNAs only), and primer extension analysis (all ncRNAs) using ncRNA-specific reverse primers that anneal ∼10 nt upstream of the conserved ncRNA motif sequences (see Supplementary Figure S13). Primer extension reactions contained 1 pM [^32^P] 5′ end-labelled oligonucleotide primer and 10 μg *Giardia* total RNA. Primer extension products and *Giardia* total RNA samples (10 μg) used for each northern blot experiment were resolved by 8% urea polyacrylamide gel electrophoresis (PAGE). RNAs were then transferred to Amersham Hybond™-XL membranes using a Bio-Rad Trans Blot Cell apparatus, according to the manufacturer’s instructions. DNA probes used for northern blots were created by PCR amplification of snRNA coding regions (also see Supplementary Figure S13) and radioactively [^32^P] 5′ end-labelled. Radioactive gels and membranes were visualized using a GE Healthcare Typhoon phosphorimager.

Mapping of mature ncRNA ends was performed using random amplification of cDNA ends (RACE) techniques. NcRNA 5′ and 3′ ends were mapped using the RACE procedures described previously (36); however, using oligonucleotide P-94 (Supplementary Figure S13) as reverse primer during the PCR step of 3′ RACE. To map 5′ ends and for detection of 5′ cap structures, we also performed RNA linker-mediated (RLM) 5′ RACE. Thirty micrograms of DNase I-treated *G. lamblia* total RNA was divided equally into three different samples as follows: (U) untreated, (C) treated with 20 U calf intestine alkaline phosphatase (CIP, New England Biolabs (NEB)) or (CT) treated with 20 U CIP and subsequently treated with 10 U tobacco acid pyrophosphatase (Interscience). Following this, for each sample, RLM-5′ RACE oligomers were ligated onto available RNA 5′ ends in 100 μl reactions containing 10 μg *Giardia* treated RNA sample (aforementioned), 3 μg RLM-5′ RACE linker oligo, 1 mM adenosine triphosphate, 50 U T4 RNA ligase I (NEB), 1× RNA ligase buffer (NEB) and 20% wt/vol polyethylene glycol 8000. Ligation reactions were incubated for 1 h at 37°C and then used directly as the template for reverse transcriptase-polymerase chain reaction (RT-PCR) using adaptor-specific forward primers (PCR step) and ncRNA-specific reverse primers (RT and PCR step). Products generated during either RT-PCR or RACE experiments were agarose gel purified using eZNA Gel Extraction Kits (Omega Biotech) (when multiple amplicons were present) or directly cloned into the CloneJet vector (Fermentas) according to the manufacturer’s protocol and subject to automated DNA sequencing (Macrogen).

### *In vitro* U4/U6 snRNA complex formation

Regions encoding the mature *G. lamblia* WB isolate U4 and U6 snRNA candidate sequences, as determined by the end mapping experiments, were PCR-amplified from *Giardia* WB genomic DNA, using appropriate reverse primers and forward primers additionally containing the T7 viral promoter sequence. All PCR products were cloned and sequenced to verify their identities. Gel-purified PCR products then served as templates for *in vitro* transcription to generate unlabelled or [^32^P] 5′ end-labelled transcripts using methods described elsewhere (37). *Giardia* U4 and U6 *in vitro* complexes were formed by assembling 20 nM radioactively labelled RNA transcript with 200 nM unlabelled RNA in the presence of 20 μM of each oligo oAH136 and oAH137, to optimize U4/U6 intermolecular base pairing (38) in assembly buffer (50 mM NaCl, 20 mM HEPES, pH 7.0, 1.5 mM MgCl_2_, 0.1 mM ethylenediaminetetraacetic acid). Reactions (15 µl) were heated to 80°C for 2 min and were then allowed to slowly cool to room temperature and were placed on ice. Complexes were then resolved on 6% native PAGE and visualized by phosphorimaging.

## RESULTS

### Identification of a conserved sequence motif in *Giardia* ncRNA genes

ncRNAs display varying modes of genomic organization, expression and maturation in different eukaryotes. In *G. lamblia,* those ncRNAs identified to date are encoded as predicted single gene transcriptional units or as dicistronic gene clusters (25,27). However, the mechanisms underlying their expression and subsequent precursor transcript processing have yet to be examined. Consequently, we examined the genomic context of previously biochemically identified *Giardia* ncRNAs searching for conserved sequence elements that may be involved in their expression and/or processing. Genomic regions encoding previously annotated *G. lamblia* WB isolate box C/D and H/ACA snoRNAs, RNase MRP RNA, and other uncharacterized ncRNAs (25–28) were aligned and inspected for recurring sequence motifs (Supplementary Figure S1). Strikingly, this analysis uncovered a highly conserved 12 nt sequence motif overlapping or residing a few nucleotides downstream of the predicted 3′ ends of the mature RNAs. We also exploited the current availability of near-complete genome sequences of three *G. lamblia* isolates, WB, GS and P15, that display substantial sequence divergence (∼77% nt identity between WB and GS in protein-coding regions) (18,39,40). BLASTN searches using *G. lamblia* WB ncRNA genomic regions as queries readily identifies orthologous P15 and GS genomic regions showing conservation of ncRNA sequences and in many cases even higher conservation of the 12 nt 3′ end sequence motif (Supplementary Figure S2).

**Figure 1.**
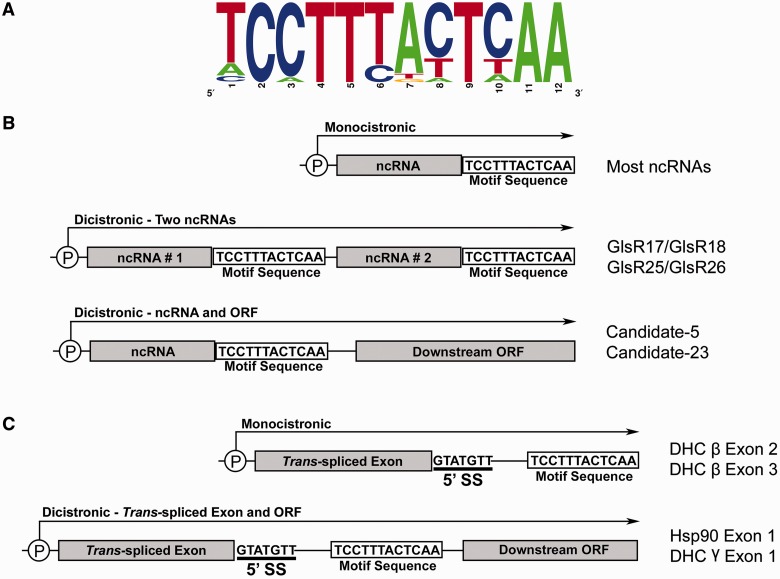
Identification of a 12 nt sequence motif within *G. lamblia* ncRNA and *trans*-intron containing genes. (**A**) Motif sequences from *G. lamblia* WB, P15 and GS isolates (*n* = 132 sequences) were used to construct a WebLogo sequence logo frequency plot (30). Nucleotide frequency at each motif position is denoted by the relative height of the letter. Genomic organization and mode of expression of *G. lamblia* ncRNAs (**B**) and 5′ *trans*-spliced intron halves (**C**) containing the 5′ splice site and showing the relative location of the processing motif. Promoter sites are indicated by a circled ‘P’ with extended arrows indicating predicted initiation sites, lengths and directionality of precursor transcripts. Representative examples of each mode of gene organization are indicated.

The collection of 3′ end motif sequences from *Giardia* WB, GS and P15 genomes (*n* = 132 sequences) revealed the consensus: 5′-[T/A/C]C[C/A]TT[T/C][A/T/C][C/T/A]T[C/T/A]AA-3′ (Figure 1A). Thirty-nine unique variations of the sequence motif were identified, with ‘TCCTTTACTCAA’ being observed in 34/132 instances (Figure 1A, Supplementary Figure S3). Motif variant prevalence is similar between *Giardia* isolates, and the motif displays strong sequence conservation with 6 of 12 positions being invariant. Because snoRNA 3′ end processing in some eukaryotes requires the formation of a SL structure (41), we examined whether the identified *Giardia* motif may participate in the formation of such structures. Mfold RNA secondary structure predictions (31) of motif variants either alone or in the context of adjacent flanking upstream and downstream sequences does not indicate significant secondary structure potential, and instead suggests the sequence motif may be exposed within single-stranded regions of an RNA primary transcript.

### The conserved motif mediates 3′ end formation of *G. lamblia* ncRNAs

**Figure 2.**
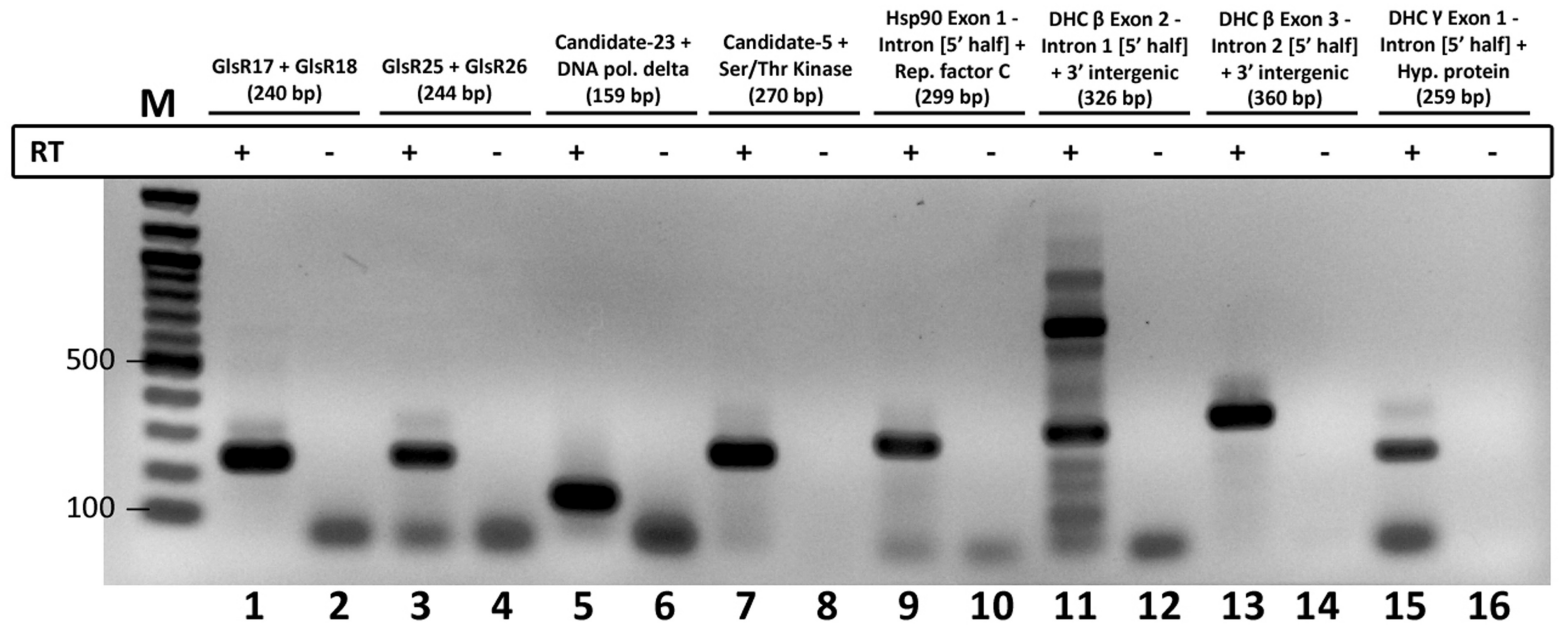
Dicistronic transcription of *Giardia* ncRNA and *trans*-spliced intron precursors. RT-PCR detection of precursor transcripts containing two different ncRNAs (designated GlsR#) (lanes 1 and 3), ncRNA (designated Candidate-#) with downstream ORF (lanes 5 and 7) or *trans*-spliced intron 5′ half with unrelated downstream ORF (lanes 9 and 15). Products of expected size (sizes indicated in parentheses; also refer to Supplementary Figure S4) were sequenced to confirm their identity. Experiments were either performed with the addition (+) or omission (−) of RT enzyme during the cDNA synthesis reaction. M = molecular weight marker, bp = base pairs. Gel image was inverted for better visualization of bands.

The conservation of the motif and its consistent position relative to predicted mature RNA 3′ ends suggests it may play an important role in *Giardia* ncRNA 3′ end formation. Additionally, several *Giardia* snoRNAs are encoded immediately upstream of annotated ORFs or other ncRNAs, with short intervening spacer sequences that contain the motif (Figure 1B). Thus, we hypothesized the motif may serve to either mediate transcription termination or post-transcriptional cleavage of precursor ncRNAs. To examine this, we used RT-PCR and 3′ RACE techniques, in conjunction with DNA sequencing of amplified products, to detect precursor transcript species and to map mature RNA 3′ ends (Figure 2 and Supplementary Figure S4). These experiments confirmed the presence of dicistronic precursor transcripts consisting of two different ncRNA species or an ncRNA with a downstream ORF. The 3′ RACE experiments determined that mature ncRNA 3′ ends are consistently within or a few nucleotides upstream of the conserved motif sequences (Supplementary Figure S5), suggesting post-transcriptional RNA cleavage of mono- or dicistronic precursor transcripts near the motif.

### The conserved sequence motif has a role in the novel *Giardia* mRNA *trans*-splicing pathway

Given the common association of the sequence motif with previously annotated ncRNAs, we predicted that we should be able to use this motif as a tool to identify other genomic regions specifying novel ncRNAs. Initially, we performed simple BLASTN searches using individual motif sequence variants against the *G. lamblia* WB genome sequence database. Surprisingly, these searches revealed canonical motif sequences residing in the 5′ halves of all four known *Giardia trans*-spliced introns previously identified by our group and others (21–23) (Figure 1C and Supplementary Figure S1). Motif sequences locate immediately downstream or overlap with those nucleotides predicted to be involved in intermolecular base-pairing interactions that mediate the *in vivo* association of the intron 5′ and 3′ halves.

We next performed RT-PCR experiments to characterize *trans*-spliced intron containing precursor transcripts. These experiments detected extended precursor mRNA species with 3′ ends extending beyond the conserved sequence motif, and in some cases, extending into downstream unrelated ORFs (Figures 1C and 2). Mapping of 3′ ends by 3′ RACE shows that precursor RNAs containing *trans*-spliced intron 5′ halves are also cleaved at their motif sequences (Supplementary Figure S5). In summary, these results indicate that some of the steps in the unusual *Giardia* mRNA *trans*-splicing pathway involve the generation of extended transcripts containing the conserved sequence motif (processing motif) that is then cleaved to generate intron 5′ halves whose ends reside directly adjacent to nucleotides predicted to mediate association to intron 3′ halves.

Because all identified *Giardia trans*-spliced introns contain the conserved motif sequence downstream of the 5′ splice site, we also searched for any instances of motif sequences within annotated protein-coding genes that may indicate the presence of additional uncharacterized *trans*-spliced introns. Searches of the *G. lamblia* WB genome identified four cases in which a motif sequence could be found within a conserved protein coding gene (Supplementary Figure S6). RT-PCR experiments confirmed expression of each of the four motif-containing genomic regions; however, 3′ RACE experiments failed to detect products corresponding to transcripts terminating near motif sequences. Unlike the previously characterized *trans*-spliced introns, these regions do not interrupt protein-coding continuity in these genes (consistent with these regions being exons), and it is interesting to note that the presence of the sequence motif alone does not always result in RNA cleavage. This may indicate a requirement for the association of motif recognition/cleavage factors with other ncRNA assembly or processing factors (or even spliceosomal components in the case of *trans*-spliced introns) that only occurs when the motif is located in the correct structural or spatial context.

### Identification of novel *Giardia* U1 and U6 snRNA candidates

We next used the high sequence conservation of the newly discovered motif to identify candidate novel *Giardia* ncRNAs, using the sequence pattern matching program ‘scan for matches’ (35). Genomic sequences for WB, GS and P15 isolates (GiardiaDB.org) were searched for instances where a *Giardia* transcription initiation site sequence (AAAAAAAAA, allowing five mismatches) (33,34) is located within 500 nt (upstream) of the RNA (processing) motif sequence. These searches produced ∼400 such matches for each *Giardia* isolate genome. Next, the candidates were examined for conservation between isolates using pairwise BLASTN comparisons. Five novel ncRNA candidates remained that maintained promoter and motif sequences in all three isolates, mapped to intergenic regions and showed significant sequence conservation in all three isolates (Supplementary Figure S2-27, 28, 29, 32 and 40).

**Figure 3.**
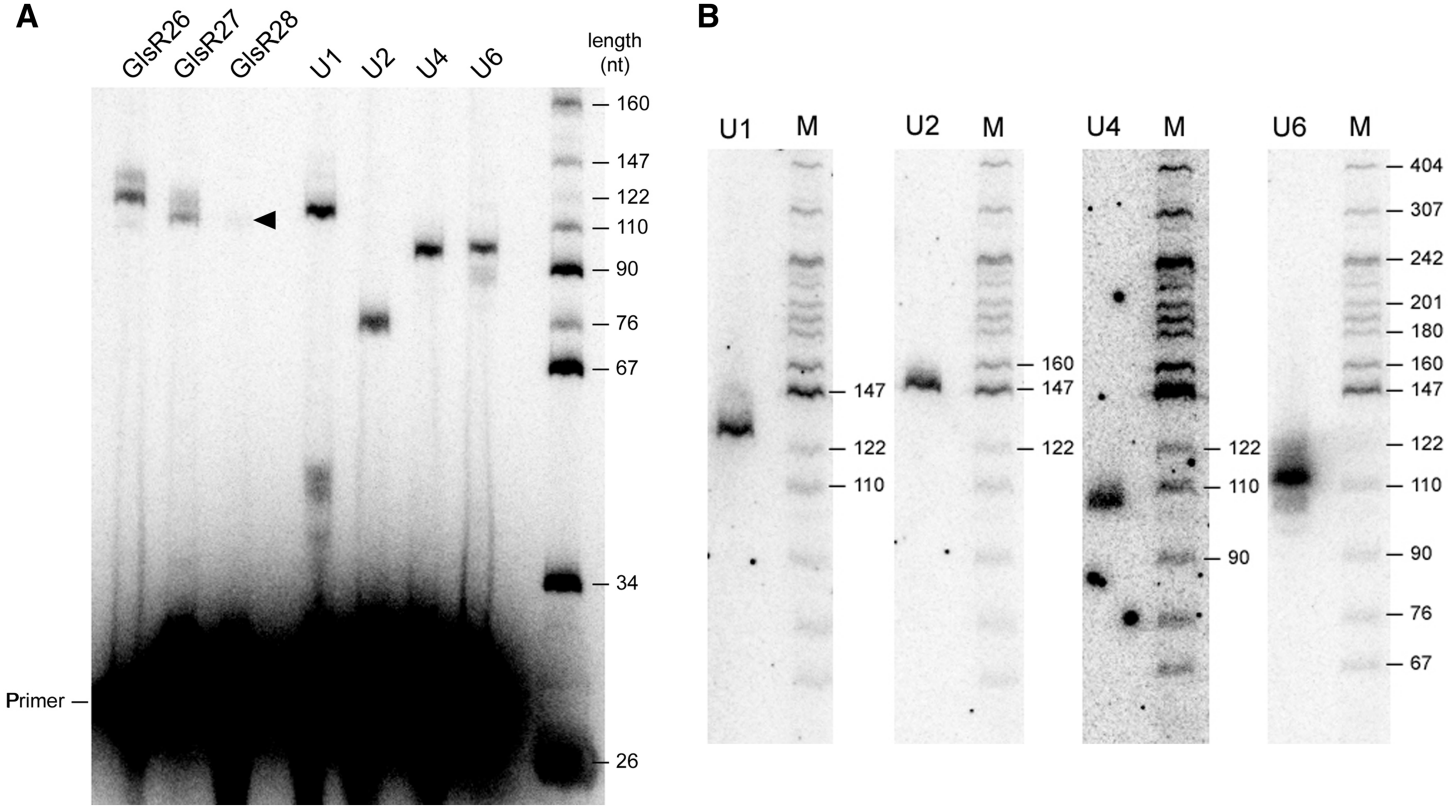
Detection of *G. lamblia* ncRNA expression. (**A**) RT-primer extension experiments were performed using gene-specific ^32^P-labelled oligonucleotides annealing directly adjacent to the RNA processing motif of each *Giardia* ncRNA (GlsR) or spliceosomal snRNA candidate. For each RT reaction, 10 μg of *G. lamblia* WB isolate total RNA was used as template with SuperScript II™ RT, and products were resolved by 8% denaturing PAGE and visualized by phosphorimaging. Rightmost lane is ^32^P- labelled *Msp* I digested plasmid pBR322 size marker, with fragment sizes indicated in nucleotides (nt). An arrowhead indicates a faint primer extension product of expected size for the GlsR28 ncRNA. (**B**) Northern blot analysis of *Giardia* snRNAs. DNA probes specific for U1, U2, U4 or U6 snRNA candidate sequences were hybridized to *Giardia* total RNA that was fractionated by 8% denaturing PAGE. The DNA size ladder (M) is the same as in part (a).

To verify *in vivo* expression of these ncRNA candidates, primer extension and northern blot analysis of total *Giardia* RNA was performed (Figure 3). Products of expected size were detected for all five ncRNA species (detected in isolate WB). Next, 5′ and 3′ RACE experiments were used to more accurately determine the sizes and map the mature 5′ and 3′ ends of each species (Supplementary Figure S5). Again, these experiments show that mature 3′ ends coincide with the conserved 3′ end sequence motif, and suggest that these RNAs may be processed by a similar mechanism to the *trans*-spliced intron containing transcripts.

Two of the novel ncRNAs, designated GlsR26 and GlsR27, show the conserved structural features of box H/ACA snoRNAs. GlsR27 is predicted to guide the formation of pseudouridine (Ψ) modification at U1745 of the *Giardia* large subunit rRNA (Supplementary Figure S7). The GlsR26 candidate is encoded immediately downstream of the GlsR25 box H/ACA snoRNA genomic sequence (26). This arrangement is similar to the previously reported organization of the GlsR17/GlsR18 snoRNA gene cluster (25) (Figure 1B, Supplementary Figure S7C). The ncRNA GlsR28 has extensive secondary structural potential but could not be assigned to any known ncRNA class (data not shown).

**Figure 4.**
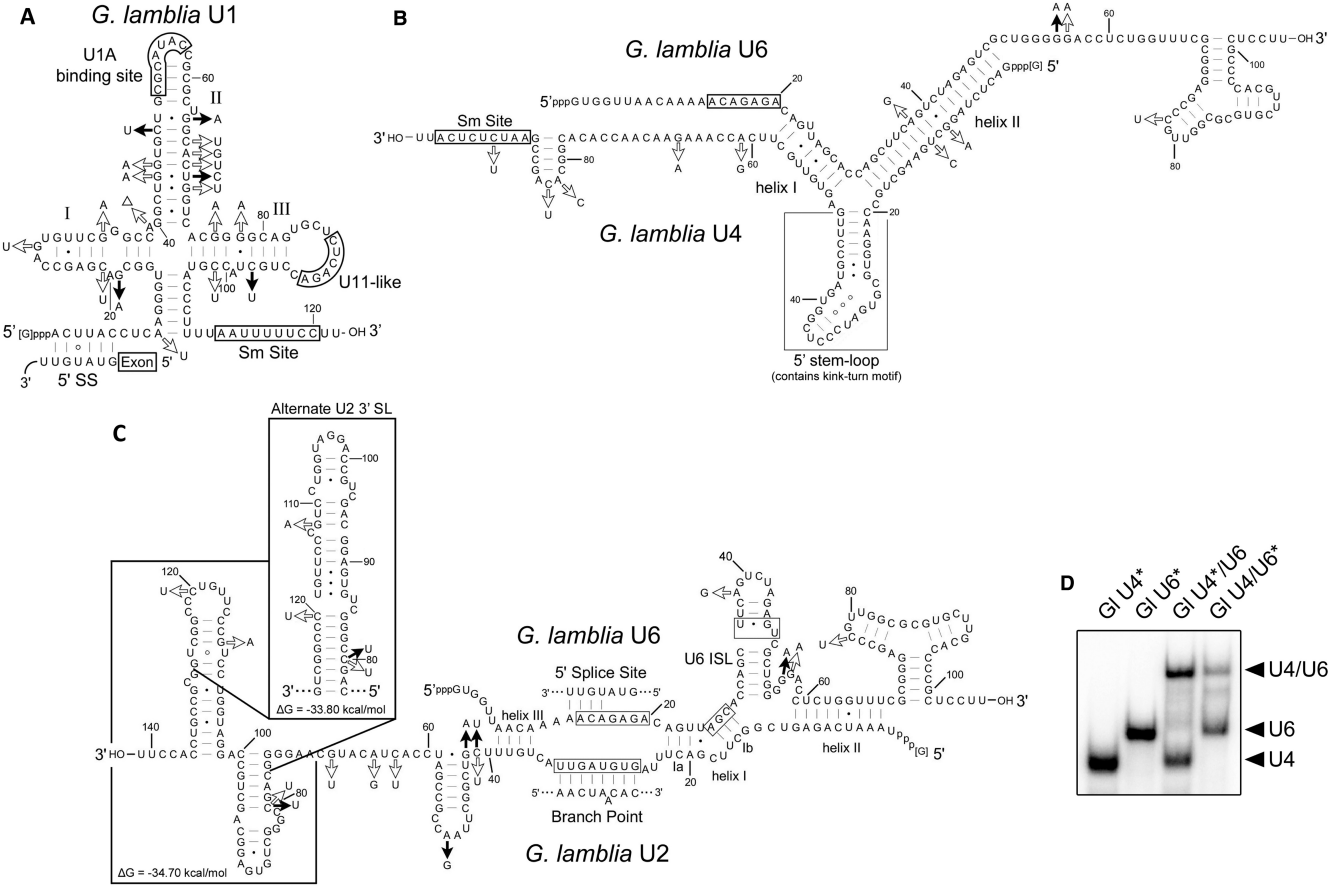
Evolutionarily divergent spliceosomal snRNAs in *G. lamblia.* Mfold (31) secondary structural predictions for *G. lamblia* WB isolate snRNA candidate sequences are shown with arrows denoting nucleotide sequence differences observed in the P15 (filled arrows) and GS (open arrows) isolates. The snRNAs are shown base pairing to the 5′ splice site (**A**) and branch-point sequence (**C**) of the Hsp90 *trans*-spliced intron (21). The two alternative SL structures for the 3′ most terminal portion of the U2 snRNA candidate are shown (C, inset) with predicted free energies. In the *Giardia* U4/U6 interaction (**B**), the 5′ stem loop structure (boxed) contains a kink-turn motif. (**D**) *G. lamblia* U4/U6 snRNA transcripts form a complex *in vitro*. *Giardia* WB isolate *in vitro* synthesized U4 and U6 transcripts were incubated either individually or together and then fractionated by 6% native PAGE and visualized by autoradiography. The asterisk indicates which transcript is radioactively ^32^P end-labelled.

**Figure 5.**
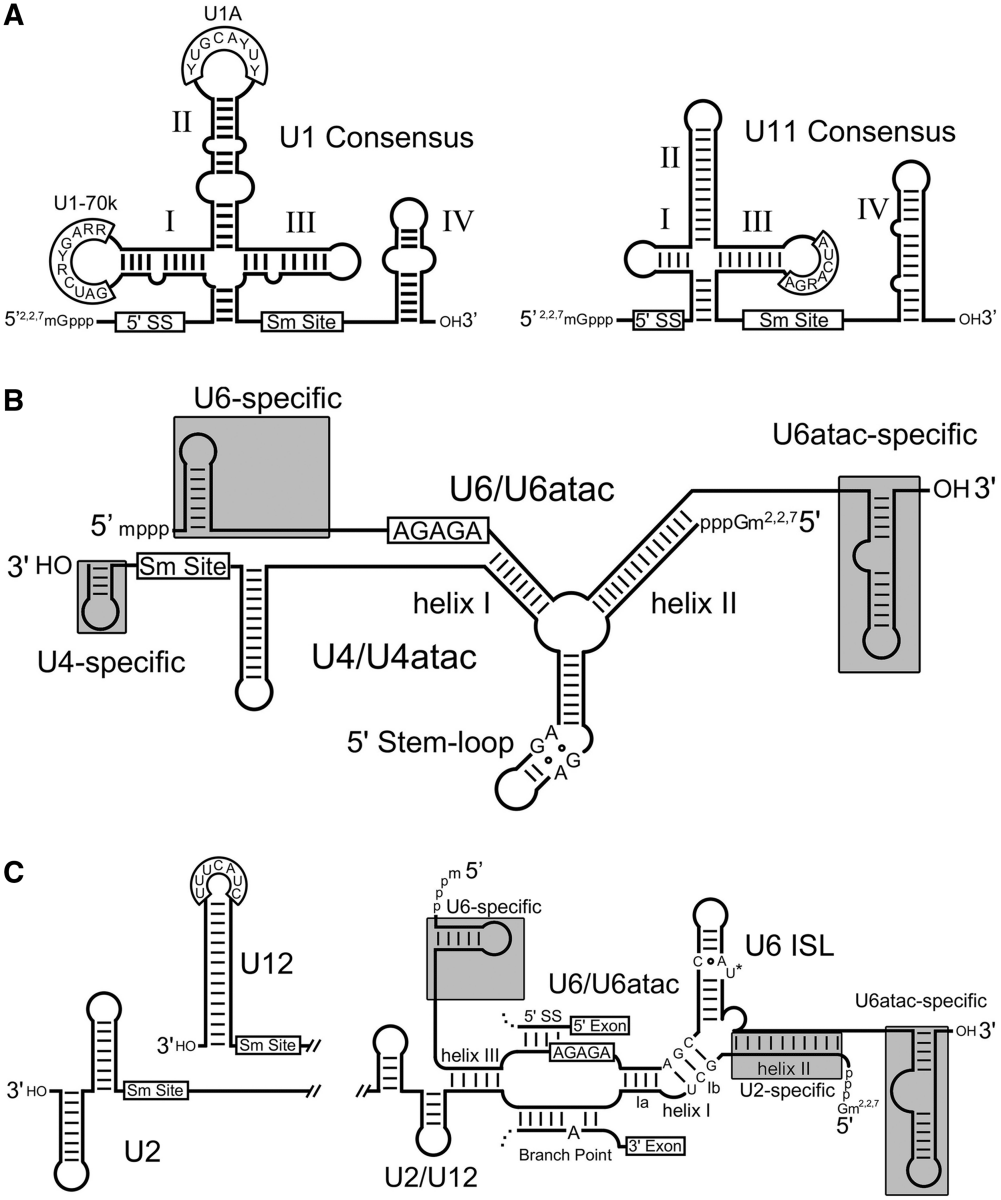
Comparison of major and minor spliceosomal snRNAs. Consensus secondary structures and conserved sequence elements (open boxes) characteristic of major and/or minor spliceosomal snRNAs are shown. (**A**) Comparison of major spliceosomal U1 to minor spliceosomal U11 snRNA, where the 5′ splice site interacting sequence (5′ SS), Sm protein binding (Sm site) or U1 specific protein binding sites are boxed. A conserved sequence of unknown function in SL III of U11 is also boxed. (**B** and **C**) Structures that distinguish major (U2, U4 and U6) and minor (U12, U4atac and U6atac) spliceosomal snRNAs are highlighted in grey boxes. The snRNAs (excluding U6) are shown containing 5′ trimethylguanosine caps as occurs in most characterized eukaryotes.

Most surprising was our finding that the remaining two ncRNAs possess conserved sequence motifs and secondary structures diagnostic of U1 and U6 spliceosomal snRNAs (Figure 4). The predicted *Giardia* U1 snRNA structure adopts the typical ‘cloverleaf’ secondary structure and contains a predicted U1A binding site sequence that is a close match to the *Saccharomyces cerevisiae* ‘CACAUAC’ sequence (42). This contrasts to the previously identified U1 candidate (24) that has an atypical predicted secondary structure lacking a recognisable U1A binding site (compare Figure 4A and Supplementary Figure S8). We mapped the 3′ end of the new U1 RNA candidate as directly downstream of the predicted Sm site, similar to the *Candida albicans* U1 snRNA, which also lacks the SL IV structure (43) that is commonly found in the U1 RNAs of other eukaryotes (Figures 4A and 5A). Another noteworthy feature of the *Giardia* U1 RNA is the lack of a conserved U1-70 kDa protein binding site sequence in SL I. A *bona fide* U1 snRNA is expected to base pair to the 5′ splice site of *Giardia* spliceosomal introns, and we observe extensive base pairing potential of the 5′ end of the U1 snRNA to the highly conserved *Giardia* 5′ splice site sequence (Figure 4A). The non-canonical U•U pairing is also observed in *S. cerevisiae* U1-5′ splice-site interactions at the same relative position (44). In humans (45) and *S. cerevisiae* (46), the two adjacent ‘U’ residues in U1 snRNA are converted to Ψ, which may be important for the function of U1 snRNA in splicing (47). As the identified *Trichomonas vaginalis* snRNAs are not 5′ capped (48), we examined the capping status of the *Giardia* snRNAs using RLM 5′ RACE (Supplementary Figure S9). Only CIP plus tobacco acid pyrophosphatase-treated *Giardia* RNA samples generated PCR amplicons containing snRNA 5′ end sequences after linker addition, RT-PCR and sequencing. These results suggest the majority of *Giardia* U1 and U2 snRNAs are 5′ capped; however, experimental results for the U4 and U6 snRNAs are ambiguous (see Supplementary Figure S9).

The U6 snRNA candidate contains the conserved ‘ACAGAGA’ and invariant ‘AGC’ trinucleotide sequences found in all known U6 snRNAs (49) (Figure 4B and C). The predicted Mg^2+^ binding site in U6 intramolecular SL (ISL) (boxed region, Figure 4C) shows a sequence differing from the typical non-canonical C•A pair followed by bulged ‘U’ residue. Similar divergence within the U6 ISL is also observed in *C. albicans*, which contains a bulged ‘C’ instead of a ‘U’ (43), and in *Trypanosoma cruzi*, which has an unusual C•C pair instead of C•A (50) (Supplementary Figure S10). Some key differences in the *Giardia* U6 snRNA candidate that we report here, as compared with the different U6 snRNA candidate previously reported (24), is the presence of complete ‘ACAGAGA’ and ‘AGC’ trinucleotide sequences that are strictly conserved between *Giardia* isolates (compare Figure 4C and Supplementary Figure S8D and F), unlike the previously predicted U6 snRNA that displays unexpected substitutions in the three isolates disrupting these functionally critical sequence elements. Our identified U6 snRNA also displays more robust base-pairing potential in the U6 ISL. We also note that sequence differences evident in the U1 and U6 snRNAs that we have identified in the three different *Giardia* isolates either occur in single-stranded regions or alternatively show compensatory mutations or formation of G•U wobble pairs that maintain the base-pairing interactions in the predicted secondary structures (Figure 4).

### Identification of novel *G. lamblia* U2 and U4 snRNA candidates

The surprising finding of a novel U6 snRNA candidate stimulated us to search for the interacting *G. lamblia* U2 and U4 snRNA candidates. In the U6/U4•U5 tri-snRNP particle, U6 snRNA forms an evolutionarily conserved three-way helical junction with U4 snRNA involving nucleotides immediately downstream of the ‘ACAGAGA’ sequence in U6 snRNA. This region of U6 base pairs extensively to the 5′ half of U4 snRNA (4) (Figures 4B and 5B). Formation of the intermolecular U4/U6 snRNA helices I and II stabilizes a 5′ SL in U4 snRNA that contains the RNA structural motif known as the kink-turn (51,52). Although the primary sequence of nucleotides involved in U4–U6 snRNA intermolecular pairing are conserved in U6 snRNAs, the interacting region of U4 is somewhat more divergent (48), but maintains the ability to form intermolecular helices I and II that are critical to spliceosome function (53,54). Using the sequence downstream of the ‘_14_ACAGAGA_20_’ sequence in the *G. lamblia* U6 snRNA candidate as query, we searched the library of previously characterized *Giardia* ncRNAs of unknown function for a potential U4 snRNA candidate that could base pair with the U6 snRNA candidate. Our analyses revealed that the ncRNA Candidate-11 (27) is capable of forming the extended base-pairing interaction with the *Giardia* U6 snRNA candidate, generating a canonical helical junction containing helices I and II of similar length to those found in other eukaryotic U4/U6 snRNA complexes. The predicted interaction is further substantiated by compensatory mutations in the U6 and U4 snRNA sequences from the *G. lamblia* GS isolate that maintain contiguous base-pairing between the two molecules (Figure 4B). Furthermore, the intermolecular pairing between the *Giardia* U6 and U4 snRNA candidates would allow for the formation of a 5′ SL in the U4 snRNA candidate containing a typical kink-turn motif with the sheared G-A base pairs (52). Inspection of the potential interaction of the previous U4 and U6 candidates (24) reveals significantly weaker base-pairing potential between the two molecules, particularly intermolecular helix II, and no conserved kink-turn motif in the U4 snRNA 5′ SL region (compare Figure 4B and Supplementary Figure S8F). In eukaryotes, the protein Snu13p is an important U4 and box C/D snoRNP assembly factor that binds to kink-turns found in box C/D RNAs and U4 snRNA. The previous identification of a well-conserved *G. lamblia* Snu13p homologue (55) that has been experimentally demonstrated to interact with kink-turn motifs (56) predicts its role in *G. lamblia* U4 snRNP assembly and is consistent with finding a conserved kink-turn in our new U4 snRNA candidate.

Next, we assessed the ability of the U6 and U4 snRNA candidates to form a complex *in vitro*. Using full-length *in vitro* synthesized transcripts of complete U4 and U6 sequences (as determined by the 5′ and 3′ RACE end mapping experiments), we performed gel mobility shift assays using either radioactively end-labelled U4 incubated with unlabelled U6 or labelled U6 incubated with unlabelled U4. In both cases, U4/U6 complexes were readily observed (Figure 4D).

After the recruitment of the U6/U4•U5 tri-snRNP to the intron, U6/U4 intermolecular base pairs are unwound, allowing for the formation of U2/U6 snRNA intermolecular helices I through III and the U6 snRNA ISL (7). During this remodelling, the ‘ACAGAGA’ sequence in U6 snRNA forms base pairs with the intron 5′ splice site, and a sequence in U2 snRNA pairs with the branch point sequence, juxtaposing intron elements for the first transesterification reaction (7). Using the same strategy as described for identification of the U4 snRNA candidate, we determined that the previously identified ncRNA Candidate-14 of unknown function (27) is capable of forming all of the conserved interactions with the U6 snRNA candidate, displaying close adherence to those interactions characterized in other eukaryotes. This includes a discontinuous helix I containing the U6 ‘AGC’ nucleotides implicated in magnesium ion binding (57) (Figure 4C). The U2 snRNA candidate exhibits an extended and canonical interaction with the highly conserved *Giardia* intron branch-point sequence (AACUAACAC, branch point ‘A’ underlined) found within the nine currently identified *G. lamblia* introns. This interaction is significantly different from the interaction between the previously proposed *G. lamblia* U2 snRNA candidate (24), and the conserved branch point sequence in which only a limited base-pairing interaction was possible that unexpectedly only includes intron nucleotides 5′ upstream of the branch point ‘A’ residue involved in the first transesterification reaction (Supplementary Figure S8B). Nucleotide changes observable in the novel U6 and U2 snRNA candidates from the *G. lamblia* GS and P15 isolates occur in predicted single-stranded regions; therefore, the intermolecular U2/U6 snRNA interactions are strictly maintained in the other *Giardia* isolates.

Next, we assessed the expression of the U2 and U4 snRNA candidates by primer extension and northern blot analysis (Figure 3A and B) and mapped their mature ends using 5′ and 3′ RACE. The experiments generated products of expected size for the *G. lamblia* U2 and U4 snRNA candidates. When using excess oligonucleotide primer, similar signal intensities for extended cDNA products from U2, U4 and U6 snRNAs are observed (Figure 3A), indicating all three snRNA candidates are likely present at similar levels *in vivo*. These experiments also seem to indicate size homogeneity (discrete ends) for each mature snRNA species. Finally, BLASTN searches of individual snRNA sequences against *G. lamblia* WB, P15 and GS isolate genomes identified only one match per genome, indicating these snRNAs are encoded by single copy genes.

Collectively, these data strongly suggest a functional role for the previously identified but uncharacterized ncRNA candidates as authentic *G. lamblia* U2 and U4 spliceosomal snRNAs. Identification of U2 and U4 snRNA candidates capable of forming evolutionarily conserved base-pairing interactions with the U6 snRNA candidate and conserved intron sequence elements also further validates our correct identification of a *bona fide Giardia* U6 snRNA.

## DISCUSSION

### *Giardia* snRNA candidates are evolutionarily divergent with properties of U2-type major and U12-type minor spliceosomal snRNAs

The identified snRNA candidates display the core structural features and nucleotide motifs conserved in spliceosomal snRNAs; however, they have noteworthy structural simplification lacking some of the evolutionarily conserved domains. Curiously, all show features also resembling U12-type (minor) spliceosomal snRNAs.

Nucleotide co-variation in the U1 snRNA sequences from *Giardia* WB, P15 and GS isolates strongly support the proposed cloverleaf secondary structure with SLs I to III having lengths similar to U1 snRNAs found in other eukaryotes (Figure 4A). The 5′ terminal sequence ‘_1_ACUUAC_6_’ is predicted to form base pairs with the conserved 5′ splice site found in *G. lamblia* introns (‘[G/A/C]UAUGUU’) similar to the interactions that occur in *S. cerevisiae,* and a conventional Sm protein binding site is located at the 3′ end of the RNA. Beyond these features, the U1 snRNA candidate is divergent, lacking SL IV and a recognizable U1-70 kDa protein binding sequence (AUCACGAA) (58). In fact, the shortened *Giardia* SL I loop sequence is more similar in size to the loops observed in the corresponding regions of U11 snRNAs. Even more intriguing is that the *Giardia* U1 snRNA candidate contains a SL III loop sequence ‘_87_CUCAGA_92_’, which is similar to the conserved ‘A**UCA**R**GA**’ sequence of unknown function which we note in the equivalent region of U11 snRNAs from diverse eukaryotes (Figure 5, Supplementary Figure S11). The *Giardia* U1 snRNA candidate SL II sequence ‘_51_CGCAUAC_57_’ (boxed, Figure 4A) is conserved between *Giardia* isolates and divergent relative to the eukaryotic consensus (U**GCA**CU**C**, identical positions in bold) (49). Interestingly, it most closely resembles the U1A binding site sequence present in *S. cerevisiae* U1 snRNA (**C**A**CAUAC**) (43); however, it is also akin to the sequence present in *T. vaginalis* U1 snRNA (U**GCAUA**U) (48), the most closely related eukaryote to *Giardia* in which snRNAs have been characterized. The apparent lack of a U1-70 kDa binding site and a divergent U1A protein binding site sequence prompted us to search for homologues of these proteins in *G. lamblia*. Consistent with previous reports (59), we could not identify clear homologues for either U1-70 kDa or U1A, or U11 snRNP-specific minor spliceosomal proteins in *G. lamblia* (10), suggesting these proteins are either highly divergent or absent.

Analysis of the *G. lamblia* U6 candidate in complex with U4 also reveals some intriguing similarities to U12-dependent spliceosomal snRNAs (Figures 4B and 5B). The U6 candidate lacks the upstream U6 snRNA-specific SL I (boxed, Figure 5B), a structure which is not present in minor spliceosomal U6atac snRNAs (8), and instead has a 5′ end position identical to U6atac RNAs (Supplementary Figure S12C). Likewise, we note that the extreme 5′ terminal sequence of the *Giardia* RNA most closely resembles U6atac RNAs. Additionally, the *Giardia* U6 snRNA candidate has an extended 3′ terminal region containing a terminal complex SL structure, more characteristic of U6atac snRNAs (60) and a structure usually not present in U6 snRNAs. The *Giardia* U4 snRNA candidate lacks a 3′ terminal SL downstream of its predicted Sm protein binding site, which is also absent in U4 snRNA from *S. cerevisiae* (61) and *C. albicans* (43). Interestingly, in metazoans, this terminal SL is present in U4 snRNA (53) but not in minor spliceosomal U4atac snRNA (60). At the primary sequence level, the *Giardia* U4 is divergent but displays some similarity to U4 and U4atac RNAs of other species (Supplementary Figure S12B).

Inspection of the *Giardia* U2 snRNA candidate and the U2/U6 interaction also shows major/minor spliceosomal characteristics. For example, the 5′ terminal nucleotides of the U2 snRNA candidate are predicted to form the extended nine base pair helix II (Figure 4C) with the U6 snRNA candidate, which is typically observed in major spliceosomal U2/U6 snRNA complexes (62) but not in the minor spliceosomal U6atac/U12 snRNA counterpart (63). The U2 candidate nucleotides in the region forming U2/U6 helices I and III and comprising the intron branch-point interacting sequence (Supplementary Figure S12A-1 and A-2) also show significantly higher sequence identity to other U2 snRNAs than to U12 RNAs (e.g. 26/39 identical nucleotides when comparing *Giardia* U2 positions 13–51 to the human U2 sequence). The downstream 3′ half of the *G. lamblia* U2 snRNA candidate is somewhat unusual, as it seems to lack a canonical Sm protein binding site before the 3′ terminal SL element(s). Sm protein homologues have been identified in *G. lamblia*, and phylogenetic analysis indicates they are divergent (19). It is, therefore, plausible that lineage-specific non-canonical Sm sites may exist. Secondary structural predictions indicate that the 3′ terminal ∼70 nt of the U2 snRNA candidate may fold into two distinct structural conformations, with nearly identical predicted thermodynamic stabilities (Figure 4C). In one conformation, the 3′ terminus folds into a dual SL structure resembling SLs III and IV of U2 snRNAs (Figure 5C, U2) (8). In the other conformation, the *Giardia* U2 snRNA candidate forms a single extended SL element reminiscent of SL III of U12 snRNAs but lacking a recognizable U12 65 kDa protein binding site sequence (CUACUUU) in the loop region (64) (Figure 5C, U12), consistent with the lack of a recognizable coding region for this protein in the *Giardia* genome. The intriguing possibility exists that both conformations may be functionally relevant in *Giardia*, and this observation further emphasizes the ‘major/minor’ hybrid nature of the *Giardia* snRNAs.

Examination of features of the predicted *Giardia* U6 snRNA candidate ISL region shows noteworthy differences from typical U6 ISL structure (Figures 4C and 5C). The Mg^2+^ binding site in U6 ISL usually contains a non-canonical C•A wobble pair and bulged uridine residue involved in metal-ion coordination (65), a feature present in U6 and U6atac snRNAs (8). The *G. lamblia* U6 snRNA candidate is instead predicted to contain a U•G wobble pair followed by bulged uridine and cytidine (we note that alternative pairing interactions are also possible). In trypanosome species, sequence variations are also observed in the U6 ISL Mg^2+^ binding site (Supplementary Figure S10), and curiously these organisms, like *Giardia*, have relatively few introns and can *trans*-splice precursor mRNAs. The *C. albicans* U6 ISL also differs by having a bulged cytidine instead of uridine. It seems that organisms containing relatively few introns and possessing more evolutionarily divergent spliceosomes display sequence variation in this region of U6. It is also interesting that the equivalent structural region of group II introns, the Mg^2+^ binding site within domain V, also show alternative sequences and non-canonical interactions (Supplementary Figure S10).

In summary, the *Giardia* spliceosomal snRNAs show some novel characteristics, in particular, a surprising number of structural similarities to both major and minor spliceosomal snRNAs. The observation of highly conserved splice site sequence motifs in the currently identified *Giardia* introns that most closely match the consensus sequences of major (U2-type) introns would initially lead one to predict the existence of a major rather than minor spliceosome in *Giardia*. However, other features of these introns make their classification less than straightforward. Recently, we identified an ‘AT-AC’ intron (21), and the first intron identified in *Giardia* in a ferredoxin gene was a ‘CT-AG’ intron (19). The collection of characterized *Giardia* introns also show an apparent fusion of branch point and 3′ splice site sequences that highly constrains the distance between the branch point adenosine and 3′ splice site. These are features commonly observed in minor (U12-type) introns; therefore, like the snRNA candidates, the introns are also showing hybrid features of major and minor spliceosomal introns.

## CONCLUSION

Collectively, we have used bioinformatic and molecular techniques to identify novel *G. lamblia* ncRNAs and characterize their expression and processing strategies, which to date are largely unexplored in this organism. In addition to identifying novel *G. lamblia* snRNAs, we find that a large number of *G. lamblia* ncRNAs (including snRNAs) are initially transcribed as longer mono- or di-cistronic precursors that are subsequently processed at the conserved 12 nt RNA sequence motif present at the 3′ downstream regions of mature ncRNAs. Surprisingly, we also identify motif sequences residing in the 5′ halves of the four known *G. lamblia trans*-introns, indicating an unexpected common RNA processing pathway for *Giardia* ncRNAs and *trans*-spliced introns. We speculate that such positioning of motif cleavage sites to liberate intron 5′ halves from longer precursor transcripts may allow for more efficient association of *trans*-intron halves for splicing, particularly when initially transcribed with downstream ORFs (e.g. Hsp90 exon1-intron 5′ half + replication factor C subunit 5; see Figure 2, Supplementary Figure S4). We also note that our RT-PCR assay cannot distinguish whether motif-cleavage occurs before or after *trans*-intron splicing. Thus, if motif cleavage occurs after *trans*-intron splicing, perhaps motif-mediated processing of the resultant Y-shaped intron may be required for expression of downstream ORFs and/or be important for subsequent *trans*-intron RNA turnover. Identification and characterization of the factors involved in motif recognition and cleavage will be important areas of future ncRNA research in *G. lamblia*.

## ACCESSION NUMBERS

*Giardia* ncRNA sequence data and annotation were submitted to GenBank. ncRNA GlsR26: Accession no. JX416859, GlsR27: JX416860, GlsR28: JX416865, U1: JX416861, U2: JX416862, U4: JX416863, U6: JX416864.

## SUPPLEMENTARY DATA

Supplementary Data are available at NAR Online: Supplementary Figures 1–13 and Supplementary Reference [66].

## FUNDING

Discovery Grant funding (to A.G.R. and J.Y.); Natural Sciences and Engineering Research Council of Canada [NSERC]. NSERC Alexander Graham Bell (CGS-D3) Graduate Scholarships (to A.J.H. and A.N.M.); University of Lethbridge Chinook Research Award (to D.E.). Funding for open access charge: Natural Sciences and Engineering Research Council of Canada—Discovery Grant.

*Conflict of interest statement*. None declared.

## Supplementary Material

Supplementary Data
