# Supplementary material for: Evolutionarily divergent spliceosomal snRNAs and a conserved non-coding RNA processing motif in Giardia lamblia
Source: Nucleic Acids Res. 2012 Sep 27;40(21):10995–1008. doi: 10.1093/nar/gks887 (PMC3510501; doi:10.1093/nar/gks887)
Supplement: Supplementary Data [file supp_40_21_10995__index.html]

Evolutionarily divergent spliceosomal snRNAs and a conserved non-coding RNA processing motif in Giardia lamblia — Evolutionarily divergent spliceosomal snRNAs and a conserved non-coding RNA processing motif in Giardia lamblia — Supplementary Data 

# Evolutionarily divergent spliceosomal snRNAs and a conserved non-coding RNA processing motif in *Giardia lamblia*

## Supplementary Data

files

**Files in this Data Supplement:**

- Supplementary Data - pdf file
