# Supplementary material for: Evolutionarily divergent spliceosomal snRNAs and a conserved non-coding RNA processing motif in Giardia lamblia
Source: Nucleic Acids Res. 2012 Sep 27;40(21):10995–1008. doi: 10.1093/nar/gks887 (PMC3510501; doi:10.1093/nar/gks887)
Supplement: Supplementary Data [file supp_gks887_nar-01834-a-2012-File015.pdf]

Supplementary Figure 1

	Genomic Location (WB)	Genomic DNA Sequence Flanking Mature RNA 3' Ends (WB isolate)
**Box C/D RNAs**		
GlsR1†	GLCHR03:412149-412256(+)	ttcaaGTCCACTGGCCTCTCCTGAGGCAGATGATGACTTTGCGACGGGCGGACGGAGGGACGCGTGACGAAGTTTGTCGTATTCTGAATTccttcatttaaaattggg
GlsR2†	GLCHR05:2883797-2883904(+)	GCAAGCGTTGCTACGAGGCGATGGAGACAAAAGCAGTTACGTTCGCAACTCTCTGAGGGTTCCTGATGCTTCCTTGGATGTCCGAGCCttcctttacttaatcgaccg
GlsR4†	GLCHR05:2609094-2609201(-)	caatttgggaagaaaaaaagtgaggcaggcAGTCTCCATGACGAGAATTACGCCGCCCCAGTCTGACCCCTGACGAACGGCTTCTCTGATCattcactcaacccgccg
GlsR5†	GLCHR03:1407731-1407838(-)	taAATTAAAAGCTGTGATGACAGGTTCTTGCCCCGTATGACCCTGCGATGAGTTATACAAAAGAACGCATCCAAGCCAACCGGCTGAGCTCcttcactcaaatcctgc
GlsR6†	GLCHR03:957087-957194(-)	aataaatcgaagtgaaacgtaaaaaatgcAATGATGGCTTGTTATCCCTGTCTGAGGTCAATACCTTGATTAGACGATTTGACAGAGCatccttcactcaacaccctt
GlsR7†	GLCHR01:808626-808733(+)	acaaaaacggacggcgtcttgccttcctcCCGCGATGATTACCGAATCACAGCGATACACGATGAAGCACTCATAGTTACTCTGAGCGGtcctttactcaacaagcaa
GlsR8†	GLCHR03:136649-136756(-)	gcagctcacagaaaaggggttCGTAGATGAAGAGAGATAAATCAGCTACCGCTGAGCCCAACGTGAGGAAGAAACCGCCTTTCGTCTGACCcttcactcaacagcccc
GlsR9†	GLCHR01:636600-636707(-)	gaaaaaagtccTAGCAACCCGTGATTTGCAACGCTTAGTCCGTGTTTCGGAGTGTCTTGCACGCTGATGAGTGAAAGCACACATGAGGTTcctttaataaaatgcaga
GlsR10†	GLCHR05:3181795-3181854(+)	agtttttacgcggtgtcgAGAATGATGAGACGTGTTCCTCTCTCCTACAGACTCCCTGGGGATGCTATGTACACCTTACTGATTTActttcctttctcaagggccat
GlsR13†	GLCHR05:4314721-4314828(-)	TATGAGATATGATGATTGGGAGCGACCTATCTTGAGGACGACGGCCGCCCGTCTTACCTTGTGACGTTTGCCGTCTTACAATGCTCTGACCctttacttaagctgccg
GlsR14†	GLCHR01:1279750-1279857(-)	ctccacgggaaccggaaataaataAAATGATGACAATGCGCATTTGTCAGAAGGCTCACTTCTGATGATTCCTCTGTCCATTCCCCTGATCCTttcctcaacaggtat
GlsR15†	GLCHR01:1329992-1330099(-)	aacccGATTCAGACTACTCCTTGGTTCCTCGCAGAATGATTATCTGTCTCCGAGCAAGCACGACTATGAGCTTACTTATGAGATCTGACTCCtttactcaatgttagt
Candidate-1ϕ	GLCHR01:1215994-1216101(-)	agcaaatgagaacaaaagcagacgAAAAAATAAATGAAGACAGAACCACAGACCTGTACTGACCCTTGATGTTAGTTGTCGCTCTGATAtcctttactcaatcgtgtc
Candidate-2ϕ	GLCHR01:33293-33400(+)	tttctattaattgaaagtcgaaaataaagTGATGATTCGAATTACCGCCCGAGGGCCCTCGGGCTCCGCTGAGGACATGCTGGTCTGACTcctttgctcaacctttcc
Candidate-13ϕ	GLCHR01:1010277-1010384(-)	aaaaccagtaaattaaaaatgattactccaaCACGACGGTCTACTGAGAACCCAGTATCTTTAGACTGCTGAGACAGTGTTATATGATTtcctttacttaaggctctc
Candidate-23ϕ	GLCHR01:449998-450105(-)	tctgtaggtagggccgatgagctattttgTACCACTCTGACCGTGAGGCGTATGCCTAGGGCATGGAGAAGAGCAGACTTGAGgcctgttcctttactcaattttgtg
**Box H/ACA RNAs**		
GlsR17†	GLCHR01:149373-149480(+)	GTGAGGATCCGGGGCACTGAGCAATCCCCAGGACACAGGCGGAGCGGAAGGCACGGCTGCGCCACGCAGCCTAATCACCGCCCCTATAGTCcttttctaaacgcgtgg
GlsR18†	GLCHR01:149494-149601(+)	CCGCTGGCGCTTGCGAGCGTGCACAGGCCTACATCCAGGGTCATAGGTGGGGAGCGGATCCCGTCCATCCTCAATCCGGGCCCGCACAGTCctttactcaagcttact
GlsR19†	GLCHR05:2415660-2415767(-)	GGCGTATGCATGTGCATAGGCTGGCCAAGCATCGTTGATAGAAGCTGCTCTTGGTCACCGGAGGGTCTCCGGTTTCATACGCAGAGACATCCttcaattaaaaacttt
GlsR20†	GLCHR04:893921-894028(-)	TACGTCTGTGTGCACAGGCGCGTCAGAGGCCGGCTAGAGCGCGACTGGTTGAGTTCCCAGAGCGATCTGGGTGATTAGCAGTCATACAGTCctttacttaagcctact
GlsR21†	GLCHR04:1151475-1151582(-)	TCGGTCCTCACCACTAGATCGGTGTTATGCTTTGTTGGGATAGCAGGCCGTGCCAGTTGGACAGCCAAGGTCCACCTCTGGTTCGGCACACATttattcaagacatct
GlsR22‡	GLCHR03:1225005-1225112(-)	GCCGGAGCCTGTGTCTCGTTCCCTGGGGCGATAGCTCTTGCTGGCAGGTCTTGCAGTGTCCATACCCGGGCAACACGTTTTCCAGCTACACCTttactcaacgtgcac
GlsR23‡	GLCHR04:1890711-1890818(-)	ACGATGGGCTAGGGAAATGCCGTGACGAGACACGCACTGGGTGGCCATTGCGTCTGCGGTAGATCCGCCGATTCCACAGCCCAGAAACACCCtttactcaagctggct
GlsR24‡	GLCHR05:1301296-1301403(+)	TTAAGGCTCCGGGGCCCGGGGCAGAGTCGGCCCTCCCAGAGCCCGCCGACGCCCCCGAGCGCCAGCCCGGCGCAGGGGCCCGGCCACACTCatttattaaacagcgat
GlsR25‡	GLCHR05:1459391-1459498(+)	ACAGCATCCTTGCACCTGCGTAGCCGATAGGTACGGGTGACCGTTTATCCCGGGCTCGTGTGGGCCCGGGTAGGCACGGTCAAAGAGTTtccttcattcaattttag
Candidate-16ϕ	GLCHR05:1006432-1006539(-)	atggagcgcggatCTGCGCTCTGCCAGATACGCCGACAGAAAGCACCAAGGAAGGATGTGGATCTCCATGTCTGCCGTGTGCGCGCATATCctttactcaatctgtgt
GlsR26*	GLCHR05:1459529-1459636(+)	TCCCGGTCCGGCCCTACCTTGCGTGCGCATATCTCCGGGATCTGCGCCGCGTCTGCTCGCGGCGATTTCGGTTATGCCGGCCCGAACACTCCttcattcaacaggccc
GlsR27*	GLCHR01:1371298-1371405(+)	AGCGCCAGCTACGTGTTATGGGCAGCGAAAGTACCAGAGCCAAAGAGTTCCTCTGATCGCCTGGCCGGAGCACATTTGTGATCTCCTATACCTtcatttaattagcgt
**Spliceosomal snRNA Candidates and other ncRNAs**		
Gl U1 Cand*	GLCHR03:661194-661301(-)	TGTTCGGGCCAGGCTGGTGCTGCGCATACCGCGCTGGCACTGGTCACGGGGCAGTGCTCTCAGACCTGCTACCGTACCCTTTTAATTTTTCCTtcacttaaggcccat
Gl U2 Cand	GLCHR04:581396-581503(-)	TTAACCGCCGATCCACTACATGCAAGGGGCAGCCGGGCTGTGAGGCAGCTGCCAGGATGGTCCTGCCCTTGTCCCGGCTGGCGCCGTCCACCTTtattcaagttttct
Gl U4 Cand	GLCHR02:1195038-1195145(+)	TAGGCTGAAGCTGCCAAGGTGCGTGATCCCTCGGTGATGCCTTGAGTGTTGCTTCACCAAAGAACAACCACACGGCACAGCCGAATCTCTCATTttttaaactttctc
Gl U6 Cand*	GLCHR04:1813414-813521(-)	AACAGAGACAGTTAGCACCAGCTTCAGTCTAGAGTCGCTGGGGGACCTCTGGTTTCGCGGGAGCCCGTTGGCGCGTGCTTGCACCCCGCTCCTtttctcaatcttcgc
RNase MRPΩ	GLCHR01:479893-480000(+)	GATCGGACACTCCCTAGCCGCCACACTGACAGTTATGGTTGCAGGACAAGCTTAGCGAGTCCGAACTCGACAGGGATACTCTACAGCGTTCCTttattcaatcattga
**NcRNAs with No Assigned Function**		
Candidate-3ϕ	GLCHR01:702197-702304(-)	CGGTGCTAGGTTCAAGCCAGGTCCAAGACCCGGGCAGTCTGTGCTGTGGGGCGCCGCTGTAGACGTCTTCCGAACACACCTGCGATAAACcctttatttaaaagatta
Candidate-5ϕ	GLCHR03:299427-299534(+)	aaatggctagcaacacgaggaaacgagtgtttcgccgggcataactgggCATGCATTTTCCTTGCCCAGTCTGCCTCCATACTAATTTCTCCTttactcaatcaggat
Candidate-12ϕ	GLCHR03:474659-474766(-)	tttgatcccccgagaaaaagaaccccaaCCCGATGACGAATAGCTGTCCTGGCGGAGGCGGTCATGACGACGAAGCCATCACGTAGGATCccttcactcaacctctgc
Candidate-15ϕ	GLCHR02:350570-350677(+)	gcCTTCAACTCAGCCGGACAGCCGGAGGCCGGAGACGGAGCACGGTCAGGCGGGCGGGGTGCAGTGCCAGCCCCAGCCGCAGAGCGGCTTCCTTtactcaagatcggg
Candidate-17ϕ	GLCHR03:1601283-1601390(-)	TACCTTGCCGCAGGGCCGTTAAGCGAGGCTTGGCCCGTGCGACGATGAGGCTCCCTGCGGGGAagccctgcggcgcgtcttaaggaggctccttcactcaacggcgtc
Candidate-21ϕ	GLCHR05:1896857-1896964(-)	CTGGACACGGCTTTGCTCTCCCACCGGAGCACATATGCTGCAGGATGACCGGCGCCTGTCTCCCACCACGTGCCAGCTAAACTGCAGCCACATTtattcaaccttttc
GlsR28*	GLCHR01:978028-978135(-)	CCCTCACCACCAGTTCTTCTCGCAGTGGTCTCTGTCAAAGACTGCCGCACGGTACACCAGAAGCAAGGGGAAGGATCCCATCCACGCAGTCCTttacttaaacatggg
***Trans*-spliced Intron 5' Halves**		
Hsp90 Intron	GLCHR05:2515303-2515410(+)	CATGTGGGACGCTCAGAAGAAGAAGACGGGCATCAAGCTCATGGTCAAGAAAGTgtatgttatgtttgtatgctgtatgtgtgcgagactcctttactcaaatttgcg
DHC β Intron 1	GLCHR03:577636-577743(+)	AAACTCTCAAGGATCAATTTGACAAGGgtatgttactgggtgaaacgctacttatgtatgtatgcttatatgtcttcgcgctcaggcgctcctttactcaattatcag
DHC β Intron 2	GLCHR05:4266366-4266473(+)	GGgtatgttttgtaatctgtgtagtcgcagtatgccattattttataacgtgtatgtcattatgtcagtatgccagtgcgctggtgagtttcctttattcaaatgttgt
DHC γ Intron	GLCHR03:967523-967630(+)	AGAATCTCAAAGCCTTATTCCGTAGCGTTGCAATGatatgttcacaggtggtttggtgtgtatgcttggcgtgtatgtgtgtatgttcctcctttactcaatacttgg

Supplementary Figure 1

Conserved 12 nucleotide sequence motif is located downstream of many ncRNAs and 5’ *trans*-spliced intron halves in *Giardia*

Comparison of DNA sequences surrounding ncRNA and *trans*-intron expressed sequences reveals a conserved motif located immediately downstream of mature RNA 3’ ends. Coding sequences (uppercase and bold) and flanking genomic sequences or intronic sequences (lowercase) for *Giardia* WB isolate ncRNAs and *trans*-spliced introns are shown with the conserved downstream sequence motif highlighted in green. Specific genomic locations for the displayed sequences are indicated as annotated for the *G. lamblia* WB isolate genome database. Predicted conserved snoRNA box elements for box C/D and H/ACA RNAs are highlighted in grey. *Trans*- spliced intron 5’ splice sites are underlined and intronic regions predicted to form base-pairing interactions with 3’ intron halves are those indicated in red text. NcRNA sequences identified by (25) [†], (26) [‡], (27) [ɸ] and (28) [Ω] are denoted. An asterisk [*] indicates ncRNAs identified in this study.

Supplementary Figure 2 – Motif sequences are conserved between *Giardia* isolates

ClustalW alignment of ncRNA genes and *trans*-spliced intron 5' halves reveals the conservation of ncRNAs and motif sequences within the *Giardia* WB, P15 and GS genomes. Predicted mature RNAs for *G. lamblia* WB isolate are in bold letters with motif sequences highlighted in green. Conserved genomic regions encoding ncRNAs and *trans*-spliced intron 5' halves are aligned.

Box C/D RNAs

1. GlsR1

Genome	Sequence	Start	End	Strand	#Nucleotides
G. lamblia WB	GLCHR03	412091	412256	+	166
G. lamblia GS	ACGJ01002434	489	654	+	166
G. lamblia P15	contig30	137430	137594	-	165

```
G. lamblia WB     CAATGTAAATCATATGTTCA--AAAAAGCAAATTAATTCCGCTTCTGATTTCATATAAAT 58
G. lamblia P15    CAATGTAAACCATG-GTTCA--TAAAAGTAAATTAATTCCGTTTTCGATTTTACATAAAT 57
G. lamblia GS     CAATATGGGCTATA--CTCAGGAAAAAATAAATTAATTTCGCTTCCGATTTTATATAAAT 58
                  **** *     **    ***   ****  ********* ** **  ***** * ******

G. lamblia WB     TTCAAGTCCACTGGCCTCTCCTGAGGCAGATGATGACTTTGCGACGGGCGGACGGAGGGA 118
G. lamblia P15    TTCAAGTCCACTGGCCTCTTCTGAGGCAGATGATGACTTTGCGACGGGCGGACGGAGGGA 117
G. lamblia GS     GTCAAATCCACCAGTCTCTCTGGAGACAGATGATGACTTTGCGACGGGCGGACGGAGGGA 118
                   **** *****  * ****   *** **********************************

G. lamblia WB     CGCGTGACGAAGTTTGTCGTATTCTGAATTCCTTCATTTAAAATTGGG 166
G. lamblia P15    CGCGTGACGAAGTTTGTCGTATTCTGAATTCCTTCATTTAAAATTGGG 165
G. lamblia GS     CACGTGACGAATTCTGTCGTATTCTGAATTCCTTCATTTAAAATTGGG 166
                  * ********* * **********************************
```

2. GlsR2

Genome	Sequence	Start	End	Strand	#Nucleotides
G. lamblia WB	GLCHR05	2883739	2883904	+	166
G. lamblia GS	ACGJ01002924	13357	13522	-	166
G. lamblia P15	contig204	1336	1501	+	166

```
G. lamblia WB     ATGAAACAAAGCTCACGCATACACAGGCTCCGGAAAAATAAATGTAGCGAACCCACGCGC 60
G. lamblia P15    ATGAAACAAAGTTCTAGCGTACACAGGCCCCGGGAAAATAAATGTAGCGAACCCACGCGC 60
G. lamblia GS     ATGAAACGAAGATCAGGTGTGCACAGGTCTCGGGAAAATAAATGTAGCGAACCCACGCGC 60
                  ******* *** **  *  * ******   *** **************************

G. lamblia WB     AAGCGTTGCTACGAGGCGATGGAGACAAAAGCAGTTACGTTCGCAACTCTCTGAGGGTTC 120
G. lamblia P15    AAGCGTTGCTACGAGGCGATGGAGACAAAAGCAGTTACGTTCGCAACTCTCTGAGGGTTC 120
G. lamblia GS     AAGCGTTGCTACGAGGCGATGGAGATAAAAGCAGTTACGTTCGCAACTCTCTGAGGGTTC 120
                  ************************* **********************************

G. lamblia WB     CTGATGCTTCCTTGGATGTCCGAGCCTTCCTTTACTTAATCGACCG 166
G. lamblia P15    CTGATGCTTCCTTGGATGTCCGAGCCTTCCTTTACTCAATCGACCG 166
G. lamblia GS     CTGATGCTTCCTTGGATGTCCGAGCCTTCCTTTACTCAATTGACCG 166
                  ************************************ *** *****
```

3. GlsR4

Genome	Sequence	Start	End	Strand	#Nucleotides
G. lamblia WB	GLCHR05	2609094	2609259	-	166
G. lamblia GS	ACGJ01001410	70458	70589	+	132
G. lamblia P15	contig19	6558	6725	-	168

```
G. lamblia WB     ATAATGCATGCGCACACTGGTCCCAAATTTTACAATAAAATCAAAAGTATTGTAAATTCA 60
G. lamblia P15    ATAATACGTGCGCATACTGGTCCCAAATTTTGTAATAAAATCTAAAGTATTTTAAATTCA 60
G. lamblia GS     ----------------------------TTT--AAGAAAGTTTGAAGTATTTTAAATTCA 30
                                              ***  ** *** *   ******* ********

G. lamblia WB     ATTTGGGAAGAAAAAAAGTGAGG--CAGGCAGTCTCCATGACGAGAATTACGCCGCCCCA 118
G. lamblia P15    ATTTAGTAAGAAAAAAAGTGGGG--CAGGCAGTCTCCATGACGAGAATTACGCCGCCCCA 118
G. lamblia GS     ATTCG--AAGGAGAAAAATGGAGGCTGGGCAGTCTCCATGATGAAAGTTACGCCGCCCCA 88
                  ***    *** * **** **  *    ************** ** * *************

G. lamblia WB     GTCTGACCCCTGACGAACGGCTTCTCTGATCATTCACTCAACCC-GCCG- 166
G. lamblia P15    GTCTGAACCCTGACGAACGGCTTTTCTGATCATTCACTCAATCCTGCCCG 168
G. lamblia GS     GTCTGACGTCTGACGAACGGCTTTTCTGATCATTTACTCAA--CAG---- 132
                  ******   ************** ********** ******  * *
```

4. GlsR5

Genome	Sequence	Start	End	Strand	#Nucleotides
G. lamblia WB	GLCHR03	1407731	1407896	-	166
G. lamblia GS	ACGJ01002910	40807	40981	-	175
G. lamblia P15	contig377	136179	136345	-	167

```
G. lamblia WB     GAAATTTCACTTGAATTTCCAATTTAATATCGTTTT----------TTGCCGACTTCCAG 50
G. lamblia P15    GAAATTTCACTTGAATTTCTAATTTAATATCGTTTTC---------TTGCAAGCTTCCAG 51
G. lamblia GS     GAAATTCAAGTAAAACTTCTAATTTAATGTTGTTTTGCCATTCCTTTTGCCACTTCTCAG 60
                  ******  * *  ** *** ******** * *****          ****    *  ***

G. lamblia WB     AATCGCACTAAATTAAAAGCTGTGATGACAGGTTCTTGCCCCGTATGACCCTGCGATGAG 110
G. lamblia P15    AATCGCATCAAATTAAAAGCTATGATGACAGGTTCTTGCCCCGTATGACCCTGCGATGAG 111
G. lamblia GS     AACC-CACTAATTTAAAAGCTGTGATGACAGGTTCTTGCCCCGTATGACCCTGCGATGAG 119
                  ** * **  ** ********* **************************************

G. lamblia WB     TTATACAAAAGAACGCATCCAAGCCAACCGGCTGAGCTCCTTCACTCAAATCCTGC 166
G. lamblia P15    TTATACAAAAGAACACATCCAAGCCAACCAGCTGAGCTCCTTTACTAAAACATTAC 167
G. lamblia GS     TTATACAAAAGAACGCACTCAAGCCAACCGACTGAGCTCCTTTACTCAAATTCTGT 175
                  ************** **  **********  *********** *** ***   *
```

5. GlsR6

Genome	Sequence	Start	End	Strand	#Nucleotides
G. lamblia WB	GLCHR03	957087	957252	-	166
G. lamblia GS	ACGJ01002918	30612	30786	-	175
G. lamblia P15	contig38	47067	47231	+	165

```
G. lamblia WB     CTACCGTGTCCTACACTCTGACACTCAGCAGCTAA-----------AGGCATTCCACCAC 49
G. lamblia P15    CTGCCGTGTCTTGCACTCTAACACTCAGCAGTTAA-----------AGGTATTCCGCCAC 49
G. lamblia GS     CTGGCACGTCTTGCACTCTGACGTTTAGTAATTAGCTTAGTACTAGAAGCGCCTTGCCGT 60
                  **  *  *** * ****** **  * ** *  **            * *       **

G. lamblia WB     AAGTAAGTGAATAAATCGAAGTGAAACGTAAAAAATGCAATGATGGCTTGTTATCCCTGT 109
G. lamblia P15    AAGTAAGTGAATAAATCAAAGTGAAACATAAAAA-TGCAATGATGGCTTGTTATCCCTGT 108
G. lamblia GS     AAACGAGTGAATAAATTAAAGCGCGA--TAAAAAATGCAATGATGGCTTGTTATCCCTGT 118
                  **   ***********  *** *  *  ****** *************************

G. lamblia WB     CTGAGGTCAATACCTTGATTAGACGATTTGACAGAGCATCCTTCACTCAACACCCTT 166
G. lamblia P15    CTGAGGCTAACCTCCTGATTAGACGATTTGGCAGAGCATCCTTCACTCAATCCCCTC 165
G. lamblia GS     CTGAGGCCAATGGCTTGATTAGATGATTTGACAGAGCATCCTTCACTCAACCTCTTC 175
                  ******  **   * ******** ****** *******************   * *
```

6. GlsR7

Genome	Sequence	Start	End	Strand	#Nucleotides
G. lamblia WB	GLCHR01	808568	808733	+	166
G. lamblia GS	ACGJ01002422	30090	30256	-	167
G. lamblia P15	contig5	23963	24128	+	166

```
G. lamblia GS       CGGCATCGGGGGTAAAGCTTGGCGTTTTTTATTGAGAATCTAATTTCAGCAAGAATTTAC 60
G. lamblia P15      CGGCATCGGGGGTAAAGCTTGGCGTTTTTTATTGAGAATTTGTTTTTACCAGGAATTTAC 60
G. lamblia WB       CGGCATCGGGGGTGAAGCTTGGCGTTTTTTATTGAGAATTTGTTTTTAACGGGAATTTAC 60
                    ************* ************************* *  *** * *  ********

G. lamblia GS       AAAAACGGACGGCGTTCTGCCTCCTTCCCCGCAATGATTACTACATCACAGCGATATAGA 120
G. lamblia P15      AAAAACAGATGGCGTTCTGCCCTCCTCCC-GCAATGATTACTGAATCACAGCGACACATG 119
G. lamblia WB       AAAAACGGACGGCGTCTTGCCTTCCTCCC-GCGATGATTACCGAATCACAGCGATACACG 119
                    ****** ** *****  ****  * **** ** ********   ********** * *

G. lamblia GS       ATGAAGCGTTCATAGTTACTCTGAGCGGTCCTTTACTCAACAGGTAA 167
G. lamblia P15      ATGAAGCGTTCATAGTTACTCTGAGCGGTCCTTTACTCAACAGATAA 166
G. lamblia WB       ATGAAGCACTCATAGTTACTCTGAGCGGTCCTTTACTCAACAAGCAA 166
                    *******  *********************************   **
```

7. GlsR8

Genome	Sequence	Start	End	Strand	#Nucleotides
G. lamblia WB	GLCHR03	136649	136814	-	166
G. lamblia GS	ACGJ01002439	4398	4564	+	167
G. lamblia P15	contig30	12902	13068	+	167

```
G. lamblia WB       TACGCCATAATGTGATGAAAAGATACTTTAAAAA-TAGATTGTATTTTAAATTCACTTTG 59
G. lamblia P15      TAAGCCATAATGTAATGAAAAGATACGTTAAAAAATAGTTTGTATTTGAAATTCACTTTC 60
G. lamblia GS       TAAGCCATAGTGTGATGAAAATGTACTCCAAAAAATAATTTGCATTTGAAATTTACTTTT 60
                    ** ****** *** *******  ***   ***** **  *** **** ***** *****

G. lamblia WB       CAGCTCACAGAAAAGGGGTTCGTAGATGAAGAGAGATAAATCAGCTACCGCTGAGCCCAA 119
G. lamblia P15      CGGTCCACGGAAAAGGAGCTCGTAGATGAAGAGAGATAAATCAGCTACCGCTGAGCCCAA 120
G. lamblia GS       AATTATATGGAAAAGGGCCTCTTAGATGAAGAGAGATAAATCAGCTACCGCTGAGCCCAA 120
                          *  *******   ** **************************************

G. lamblia WB       CGTGAGGAAGAAACCGCCTTTCGTCTGACCCTTCACTCAACAGCCCC 166
G. lamblia P15      CGTGAGGAAGAAACCGCCTTTCGTCTGACCCTTCACTCAACAGCCCC 167
G. lamblia GS       CGTGAGGAAGAAACCGCCTTTCGTCTGATCCTTCACTCAACAGCTCC 167
                    **************************** *************** **
```

8. GlsR9

Genome	Sequence	Start	End	Strand	#Nucleotides
G. lamblia WB	GLCHR01	636600	636765	-	166
G. lamblia GS	ACGJ01002928	11774	11939	-	166
G. lamblia P15	contig348	14517	14692	-	176

```
G. lamblia WB       CAAAATCCATACTAAA----------AAATGGATGACAGTAATCATATTAAATTGCATTG 50
G. lamblia P15      CAAAGTTCATGCTAAATCATGCCAAAAAATGAATGATAGTAACCATATTAAATTGCATTA 60
G. lamblia GS       CAAAGTTCATGCTCAA----------AAATGAATGACAGTAACTATATTAAATTACATTG 50
                    **** * *** ** **          ***** **** *****  ********** ****

G. lamblia WB       CATGTGGGGAAAAAAGTCCTAGCAACCCGTGATTTGCAACGCTTAGTCCGTGTTTCGGAG 110
G. lamblia P15      CTGACAAGGAAAAAAGTCTTAGCAACCCGTGATTTGCAACGCTTAGTCCGTGTTTCGGAG 120
G. lamblia GS       CTGGCAGGGAAAAAAGACTAGATAACCCATGATTTGCAATGCTTAGTCCGTGTTTCGAAG 110
                    *      ********* *     ***** ********** ***************** **

G. lamblia WB       TGTCTTGCACGCTGATGAGTGAAAGCACACATGAGGTTCCTTTAATAAAATGCAGA 166
G. lamblia P15      TGTTTTGCACACTGATGAGTGAAAGCACACATGAGGTTCCTTTAATAAAATGCAAA 176
G. lamblia GS       TGTTTTGCACGCTGGTGAGTGAAAGCACACATGAGGTTCCTTTAATAAAATGCAGA 166
                    *** ****** *** *************************************** *
```

9. GlsR10

Genome	Sequence	Start	End	Strand	#Nucleotides
G. lamblia WB	GLCHR05	3181737	3181902	+	166
G. lamblia GS	ACGJ01002075	5843	6008	+	166
G. lamblia P15	contig161	35239	35404	+	166

```
G. lamblia WB     GTTCGTTTCCGGATTGCTCGTTTCCAGTTGTAAATTAATTTAAAGTGAATTCTTCTCAAG 60
G. lamblia P15    GTTCGTTTCCGGATTGCTCGTTTCCAGTTGTAAATTAATTTAAAATGAATTCTTCCCGAA 60
G. lamblia GS     TTTCGTTTCCAGATTGTCTGTTTCCAATTATGAACCAATTTAAAATGAATTCTCTCAGGC 60
                   ********* *****   ******* ** * **  ******** ********

G. lamblia WB     TTTTTACGCGGTGTCGAGAATGATGAGACGTGTTCCTCTCTCCTACAGACTCCCTGGGGA 120
G. lamblia P15    TTTTCGTGTGATGCTGAGAATGATGAGACGTGTTCCTCTCTCCTACAGACCTCCTGGGGA 120
G. lamblia GS     TTTTTGCGCCAGGCCAAAGATGATGAGACGTGTTCCTCTCTCCTACGGACACCCTGGGGA 120
                  ****   *    *   *  *************************** ***  ********

G. lamblia WB     TGCTATGTACACCTTACTGATTTACTTTCCTTTTCTCAAGGGCCAT 166
G. lamblia P15    TGCTATGTACACCTTACTGATTTACTTTCCTTTTCTCAAAGGCTAT 166
G. lamblia GS     TGCCATGTACACCTTACTGACTTACTTTCCTTCTCTCAAGATCTAG 166
                  *** **************** *********** ******   * *
```

10. GlsR13

Genome	Sequence	Start	End	Strand	#Nucleotides
G. lamblia WB	GLCHR05	4314721	4314886	-	166
G. lamblia GS	ACGJ01002906	52793	52956	-	164
G. lamblia P15	contig39	120508	120671	-	164

```
G. lamblia WB     AGTCATCTATTTAGAATTGGAATTAGACTTTGAAATTCATCGCCCTCCGATCCATTCGTA 60
G. lamblia P15    --TCATCTATTTAGAATTGGAATTAGACTTTGAAATTCATCGCCTCCCGATCCATTCGTA 58
G. lamblia GS     AATCATCTATTTAGAATCAGAATCGGGTTTTAAAACTCTTCCCTCTCCAATCCATTCGTG 60
                    ***************  ****  *  *** *** ** ** *   ** **********

G. lamblia WB     TGAGATATGATGATTGGGAGCGACCTATCTTGAGGACGACGGCCGCCCGTCTTACCTTGT 120
G. lamblia P15    TGAGATATGATGATTGGGAGCAACCTATCTTGAGGATGGCGGCCGCCCGTTTTACCTTGT 118
G. lamblia GS     TGAGATATGATGATTGGGAGCGACCTATGTTGAGGATGGCGGCTGCCCGTCTTACCTTGT 120
                  ********************* ****** ******* * **** ****** *********

G. lamblia WB     GACGTTTGCCGTCTTACAATGCTCTGACCCTTTACTTAAGCTGCCG 166
G. lamblia P15    AACGTTTGCCGTCTTACAATGCTCTGACCCTTTACTTAAGCTGCTG 164
G. lamblia GS     GACTTCTGCCGTCTTGCAATGCTCTGACCCTTTACTCAAGCCAC-- 164
                   ** * ********* ******************** ****  *
```

11. GlsR14

Genome	Sequence	Start	End	Strand	#Nucleotides
G. lamblia WB	GLCHR01	1279750	1279915	-	166
G. lamblia GS	ACGJ01002915	13366	13500	-	135
G. lamblia P15	contig173	79729	79895	+	167

```
G. lamblia WB     CACAGACAAA-ACTAATCCACCAGTAGAATGACGAGGGGGTACACCGACAGCGGTTGATC 59
G. lamblia P15    --TATAAAAA-ACTAATCCGTCAGTAGAATGACGAGAAAATACACTGACAGCAGCTGACT 57
G. lamblia GS     TATAGCTAAATGCTATTTAGTTGATAG--CTGCGA------------ACAGC-------- 38
                     *   *** *** *       ***     ***            *****

G. lamblia WB     TCCAC---GGGAACCGGAAATAAATAAAATGATGACAATGCGCATTTGTCAGAAGGCTCA 116
G. lamblia P15    TCCACCACGGGGGCTGAAAATAAATAAAATGATGACAATGCGCATTTGTCGGAAGGCTTA 117
G. lamblia GS     -------------CTAGGAATAAATAAAATGATGATAATGCGCATTTGTCGGAAGGCTCA 85
                               *    ***************** ************** ******* *

G. lamblia WB     CTTCTGATGATTCCTCTGTCCATTCCCCTGATCCTTTCCTCAACAGGTAT 166
G. lamblia P15    CTTCTGATGATTCCTCTGTCCATTCCCCTGACCCTTTGCTCAATAGGTAT 167
G. lamblia GS     CTTCTGACGATTCCTTTGTCCATTCCCCTGACCCTTTATTCAAAGATCAA 135
                  ******* ******* *************** *****  ****     *
```

12. GlsR15

Genome	Sequence	Start	End	Strand	#Nucleotides
G. lamblia WB	GLCHR01	1329992	1330157	-	166
G. lamblia GS	ACGJ01002916	840	1001	-	162
G. lamblia P15	contig173	30316	30481	+	166

```
G. lamblia WB      AAGAGGCTGCGACGCGGGTTATTCAGTTCGATGCGCCCAGGCTGACGGTAGGACGCCTAA 60
G. lamblia P15     AAGAGGCCGCGACGTGGGTTGTTCAGTTCGATGCGCCCAGGCTGACGGTAGGACGCCTAA 60
G. lamblia GS      AAGGGGCTGCGACGCGGGTTGTTCAGTTCGATGCGCTCAGGCTGACAGTAGGACGCCTAA 60
                   *** *** ****** ***** *************** ********* *************

G. lamblia WB      CCCGATTCAGACTACTCCTTGGTTCCTCGCAGAATGATTATCTGTCTCCGAGCAAGCACG 120
G. lamblia P15     CTCGATTCAGACTACTCCTTGGTTCCTTGCAGAATGATTATCCGTCTCTGAGCAAGTGCG 120
G. lamblia GS      CTCAATTCAGACTACTCCTCGATCCTTCGCAGAATGATTATCTATCTCTGGGCAAGCGTG 120
                   * * *************** * * * * **************  **** * *****   *

G. lamblia WB      ACTATGAGCTTACTTATGAGATCTGACTCCTTTACTCAATGTTAGT 166
G. lamblia P15     ACTATGAGCTTACTTATGAGATCTGACTCCTTTACTCAATGTCAGA 166
G. lamblia GS      GCTATGAGCTTACTTATGAGATATGACTCCTTTACTCAATGA---- 162
                    ********************* ******************
```

13. Candidate-1 [as named in (27)]

Genome	Sequence	Start	End	Strand	#Nucleotides
G. lamblia WB	GLCHR01	1215994	1216159	-	166
G. lamblia GS	ACGJ01002331	42201	42365	+	165
G. lamblia P15	contig25	103482	103675	+	194

```
G. lamblia WB      CAGCAAGTTCAAGTCTGGGAACCGAGATCGTTTCAAAAACGGTTTTAAAAAGC----TCC 56
G. lamblia P15     TCGGAAGTCTAAATCCAGAGACCAAAGTCGTTTAAAAAATGATTTTAAAGAGCGAGCTTT 60
G. lamblia GS      CAAAAAATTCAACACCAGAGACAAAATCTGAGTCAAAAACAGTTTTAAA-AGC----CCC 55
                       ** *  **  *  *  **  *    *  * *****   ******* ***

G. lamblia WB      GAAGCAAATGAGAACAAAAGCA-GACGAAAAAATAAATGAAGACAGAACCACAGACCTGT 115
G. lamblia P15     GAAGCAAATGAGAAACAAAATATGAAAAACAAAAAAATGAAGACAGAACCACAGACCTGT 120
G. lamblia GS      GAAATAAAT-------------------------AAATGAAGATAGAACCACAGACCTGT 90
                   ***  ****                         ********* ****************

G. lamblia WB      ACTGACCCTTGATGTTAGTTGTCGCTCTGATATCCTTTACTCAATCGTGT-C-------- 166
G. lamblia P15     ACTGACTCTTGATGTTAGTTGTCGCTCTGATATCCTTTACTCAATCATTT-CTGGGACTA 179
G. lamblia GS      ACTGACTATTGATGTTAGTTGTCGCTCTGATACCCTTTACTCAAGCTTTTTCAAGTGTCG 150
                   ******  ************************ *********** * * * *
```

14. Candidate-2

Genome	Sequence	Start	End	Strand	#Nucleotides
G. lamblia WB	GLCHR01	33235	33400	+	166
G. lamblia GS	ACGJ01002208	5884	6052	+	169
G. lamblia P15	contig52	39757	39923	-	167

```
G. lamblia WB      GTCTTT-TTCCAGAATTTGTTCCTTTCAGTGTTTAGTGTCTTT-GTCTTTTATCTTAGC- 57
G. lamblia P15     GTCTTT-TTCCAAAATTCGTCTCTTTCAACATTTAATGTTTTTTGTCTTTTACTCTAAC- 58
G. lamblia GS      GTCTGTGTTCCAAAATTTGCTCCCTTCCATTTTTAAGTTTTTTTGGCTTTTA--TTAATC 58
                   **** * ***** **** *   * ***    ****   * *** * ******   **

G. lamblia WB      -TTTTCTATTAATTGAAAGTCGA-AAATAAAGTGATGATTCGAATTACCGCCCGAGGGCC 115
G. lamblia P15     -TTTCTTATTAATTGAAGGATGA-AAATAAAGTGATGATCCGAATTACCGCCCGAGGGCC 116
G. lamblia GS      ATTTTCTATTAATTGAGAATCGCTAAATAAAGTGATGATCCGAATTACCGCCCGAGGGCC 118
                    ***  **********     *  *************** ********************

G. lamblia WB      CTCGGGCTCCGCTGAGGACATGCTGGTCTGACTCCTTTGCTCAACCTTTCC 166
G. lamblia P15     CTTGGGCTCTGCTGAAGACATGCTGGTCTGATTCCTTTGCTCAACCTTTCT 167
G. lamblia GS      TTCGGGCTCCGCTGAGGACATGCTGGTCTGACTCCTTTTCTTAATCTTTCT 169
                    * ****** ***** *************** ****** ** ** *****
```

15. Candidate-13

Genome	Sequence	Start	End	Strand	#Nucleotides
G. lamblia WB	GLCHR01	1010277	1010442	-	166
G. lamblia GS	ACGJ01002748	30795	30925	-	131
G. lamblia P15	contig59	121266	121421	+	156

```
G. lamblia WB     TAGGTATACTTTGTGCGGACTAGAAACGAACTAGAAAATCAGTAAA-AAGGTCTTGAGCA 59
G. lamblia P15    TAAATGTACTTTGTGAG-----------AACTAGAAAGCCAGTAAATAAGGTCAAAAACA 49
G. lamblia GS     --CACGTGTTCTG-GCG---------------TGAAA-----TAAATAA----------- 26
                        *  * ** * *                ****     **** **

G. lamblia WB     AAACCAGTAAATTAAAAATGATTACTCCAACACGACGGTCTACTGAGAACCCAGTATCTT 119
G. lamblia P15    AAGCCAACAAATAAAAAATGATTACTTTAACACGACGGTCTGCTGAGAACCCAGTACCTT 109
G. lamblia GS     --ATCAGTAAATAAAAAATGATTACTCCAACACGACGGTCTGCTAAGAACCCAGTATCTT 84
                      **  **** *************  ************* ** *********** ***

G. lamblia WB     TAGACTGCTGAGACAGTGTTATATGATTTCCTTTACTTAAGGCTCTC 166
G. lamblia P15    TAGACTGCTGAGATAGTGTTATATGATTTCCTTTACTTAAAGTTCAC 156
G. lamblia GS     TAGACTGCTGAGACAGTGTTGTATGATTCCCTTTATTCAAACATCTC 131
                  ************* ****** ******* ****** * **   ** *
```

16. Candidate-23

Genome	Sequence	Start	End	Strand	#Nucleotides
G. lamblia WB	GLCHR01	449998	450163	-	166
G. lamblia GS	ACGJ01001805	1801	1965	+	165
G. lamblia P15	contig818	118027	118189	+	163

```
G. lamblia WB     GAGGCATGTATAATTATACCAAAATTAATTGCAGAGTTCTCCTTTTTCAAAAAGCCTCTC 60
G. lamblia P15    -GGGTGTGTGTAATTATACACAAATTAATTGCAAAATTCT--TTTTTTAAAAAGCCTCTC 57
G. lamblia GS     -GGGGATGTATAATTATACACAAATTAATTGTAAAGTCGACTTTTCTCAAAATGCCCCTC 59
                    **  *** *********  ********** * * *     *** * **** *** ***

G. lamblia WB     TGTAGGTAGGGCCGATGAGCTATTTTGTACCACTCTGACCGTGAGGCGTATGCCTAGGGC 120
G. lamblia P15    TGTAGGCAGGGCCAATGAACTGTTTTGTACCACTCTGACCGTGAGGCATGCGCCTAGGGC 117
G. lamblia GS     CGTGGGGGAGGCCGATGAGCTATTTTGTACCACTCTGACTGCAGGGCGTACGCCCAGGGT 119
                   ** **   **** **** ** ***************** *   *** *  *** ****

G. lamblia WB     ATGGAGAAGAGCAGACTTGAGGCCTGTTCCTTTACTCAATTTTGTG 166
G. lamblia P15    ATGGAGAAGAGCAGACTTGAGGCCCGTTCCTTTACTCAATTTTGTG 163
G. lamblia GS     ATGGAGAAGAGCAGACTTGAGGCCTATTCCTTTATTCAATTGTCCG 165
                  ************************  ******** ****** *  *
```

Box H/ACA RNAs

17. GlsR17

Genome	Sequence	Start	End	Strand	#Nucleotides
G. lamblia WB	GLCHR01	149315	149480	+	166
G. lamblia GS	ACGJ01001410	76605	76772	+	168
G. lamblia P15	contig11	8260	8426	+	167

```
G. lamblia WB     GGTCAGTTTCTAG-ACCTCCTGGGATAATGCGCTTCTTTGAGCCGCGGGTTTACTCGTGG 59
G. lamblia P15    GGTCAGTTTCTAG-ACCTCCTGGGATAATGCGCTTCTTTGAGCCGCGGGTTTACTCGTGG 59
G. lamblia GS     AAGCAGTTTCTAGCACCTCCTGAGTTAATGCGCTTCTTTGAGCCGCGGGTTTACTCGTGG 60
                     ********** ******** * ***********************************

G. lamblia WB     TGAGGATCCGGGGCACTGAGCAATCCCCAGGACACAGGCGGAGCGGAAGGCACGGCTGCG 119
G. lamblia P15    TGAGGATCCGGGGCACTGAGCAATCCCCAGGACATAGACGGAGCGGAAGGCACGGCTGCA 119
G. lamblia GS     TGAGGATCCGGGGCACCGAGCAATCCTCAGGACACAGACGGAGCGGAAGGCACGGTTGTG 120
                  **************** ********* ******* ** ***************** **

G. lamblia WB     CCACGCAGCCTAATCACCGCCCCTATAGTCCTTTTCTAAACGC-GTGG 166
G. lamblia P15    CTGTGCAGCCTAATCACCGCCCCTATAGTCCTTTTCTAAACCTTATGG 167
G. lamblia GS     TGACGCAACCTAATCACCGCCCCGACAGTCCTTCGCTTAATTATGCGG 168
                      *** *************** * *******  ** **      **
```

18. GlsR18

Genome	Sequence	Start	End	Strand	#Nucleotides
G. lamblia WB	GLCHR01	149436	149601	+	166
G. lamblia GS	ACGJ01001410	76727	76894	+	168
G. lamblia P15	contig11	8381	8548	+	168

```
G. lamblia WB     ACGCAGCCTAATCACCGCCCCTATAGTCCTTTTCTAAACGC-GTGGCCGGTGCAGCTGCC 59
G. lamblia P15    GTGCAGCCTAATCACCGCCCCTATAGTCCTTTTCTAAACCTTATGGCTGGTGCAGCCGCC 60
G. lamblia GS     ACGCAACCTAATCACCGCCCCGACAGTCCTTCGCTTAATTATGCGGCCGGTGCGGCTGCC 60
                    *** *************** * *******  ** **      *** ***** ** ***

G. lamblia WB     CGCTGGCGCTTGCGAGCGTGCACAGGCCTACATCCAGGGTCATAGGTGGGGAGCGGATCC 119
G. lamblia P15    CGCCAGTGCTTGCGGGTGTGCACAGGCCTACATCTAGGGTCATAGGTGGGGAGCGGATCC 120
G. lamblia GS     CACTGGTACCTTTGGGCGCGCACAGGCCCACAGCCGGGGTCATAGGTGGGGAGCGGATAC 120
                  * *  *  * *  * * * ********* *** *  ********************** *

G. lamblia WB     CGTCCATCCTCAATCCGGGCCCGCACA-GTCCTTTACTCAAGCTTACT 166
G. lamblia P15    TGTCCATCCTCAATCCGGGCCCGCACATGTCCTTTATTCAAGTTTACT 168
G. lamblia GS     TGTCCATCCTCAATCCGGGCCCTCACATGTCCTTTACTCAAATTTCAT 168
                   ********************* **** ******** ****  **  *
```

19. GlsR19

Genome	Sequence	Start	End	Strand	#Nucleotides
G. lamblia WB	GLCHR05	2415660	2415825	-	166
G. lamblia GS	ACGJ01001859	18642	18807	-	166
G. lamblia P15	contig10	39723	39888	-	166

```
G. lamblia WB       TGGAGGCTCGGCGTCTCGTTCTGGAAAAGCAAGCAGAAGCCCAGTTTGGTCTCTACCGG 60
G. lamblia P15      TGGAGGCTTGGCATCCCGTTCTGGAAGGGCAAGCGGAGATCCAGTCTGGTCTCTACCAG 60
G. lamblia GS       TGGAGGTCTGACGTCCCGTTCTGGAGGGGAGGATGGAAGCCTAGCTTGGTCACTACCAG 60
                    ******   * * ** **********   *      **   * **  ***** ***** *

G. lamblia WB       CGTATGCATGTGCATAGGCTGGCCAAGCATCGTTGATAGAAGCTGCTCTTGGTCACCGGA 120
G. lamblia P15      CGTATGCATGTGCGTAAGCTGGTCAAGCATCGTTGATAGAAGCTGCTCTTGGTCACCGGA 120
G. lamblia GS       TGTGCACATGTGTGTAGACTAGTCAAGCATCGTTGATACAAGCTGCTCTTGGTCACCGGA 120
                     **   ******  **  ** * *************** *********************

G. lamblia WB       GGGTCTCCGGTTTCATACGCAGAGACATCCTTCAATTAAAAACTTT 166
G. lamblia P15      GGGTCTCCGGTTTCATACGCAGAGACATCCTTCAATTAAAAACTTT 166
G. lamblia GS       GGGCCTTCGGTTTCATATGCAGAGACATCCTTCAATTAAAAACTTT 166
                    *** ** ********** ****************************
```

20. GlsR20

Genome	Sequence	Start	End	Strand	#Nucleotides
G. lamblia WB	GLCHR04	893921	894086	-	166
G. lamblia GS	ACGJ01000491	21037	21190	+	154
G. lamblia P15	contig4	36319	36488	-	170

```
G. lamblia WB       CCATCCAGTTTGATA----GGGGGT-TCTTTTCTTTTTGCCAAGTTAAAAATGCCAGCTG 55
G. lamblia P15      CCATCCAGTGTTAAATGATGGGGGT-TCTTTTCTTTTTGCTAAGTTAAAAATGCCAGCTA 59
G. lamblia GS       CCATC-AATGTTGACGCTTGAAGTTATCTTTTTCTTTTG----GTTAAAAATGCCAGCTA 55
                    ***** * * *        *  * * ******  *****    ****************

G. lamblia WB       AGTTACGTCTGTGTGCACAGGCGCGTCAGAGGCCGGCTAGAGCGCGACTGGTTGAGTTCC 115
G. lamblia P15      AGTTACGTCTGTATATACAGACGCGTCAGAGGCTGGCTAGAACGCGACTGATTGAGTTCC 119
G. lamblia GS       AGTTACGTCTGTATGTACAGGCGCGTCAGAGGTTGGCTAGAGCGTGACTGGTTGAGTTCC 115
                    ************ *  **** ***********  ******* ** ***** *********

G. lamblia WB       CAGAGCGATCTGGGTGATTAGCAGTCATACAGTCCTTTACTTAAGC----CTACT----- 166
G. lamblia P15      CAGAGCAATCTGGGTGATTGGCAGTCATACAATCCTTTACTTAACT----CTACT----- 170
G. lamblia GS       TGGGGAAGCCTGGGTGATTAACAGTCATATAGTCCTTTACTCAAGTTGGGCTTCCAGTCT 175
                      * *    **********  ******** * ********* **      ** *
```

21. GlsR21

Genome	Sequence	Start	End	Strand	#Nucleotides
G. lamblia WB	GLCHR04	1151475	1151640	-	166
G. lamblia GS	ACGJ01002311	35663	35829	-	167
G. lamblia P15	contig696	15261	15427	+	167

```
G. lamblia WB       ACATTTTAATTGTCG-CTTCAAGCAAAAGTGTACTGTATAAAAACCAATATTACTACCAT 59
G. lamblia P15      ACATTTTAATTGCCG-TTTCAAGCAAAAGTGTACTGTATAAAAACCAATATTACTACCAT 59
G. lamblia GS       ACATTTTAATTGTTGGTTTCTAGCAAA-GTATACTGCATAAAAACCAATATTACTACCAT 59
                    ************  *  *** ****** ** ***** ***********************

G. lamblia WB       CGGTCCTCACCACTAGATCGGTGTTATGCTTTGTTGGGATAGCAGGCCGTGCCAGTTGGA 119
G. lamblia P15      CGGTCCTCACCACTAGATCGGTGCTATGCTTTGTTGGGACAGCAGGCTGTGCCAGTTGGA 119
G. lamblia GS       CGGTCCTCACTACTAGATCGGTGTTATGCTTTGTTGGGATAACATGCCGTGCCAATCAGG 119
                    ********** ************ *************** * ** ** ****** *  *

G. lamblia WB       CAGCCAAGGTCCACCT-CTGGTTCGGCACACATTTATTCAAGACATCT 166
G. lamblia P15      CAACTAAGGTCCATCTACTGGTCCAGCACACATTTATTCAAGATATTT 167
G. lamblia GS       CAGCTAGTGTCTCTCTACTGGTGCGGCATACATTTATTCAAGACATCT 167
                    ** * *  ***   ** ***** * *** ************** ** *
```

22. GlsR22

Genome	Sequence	Start	End	Strand	#Nucleotides
G. lamblia WB	GLCHR03	1225005	1225170	-	166
G. lamblia GS	ACGJ01002895	16172	16337	-	166
G. lamblia P15	contig18	1797	1962	-	166

```
G. lamblia WB       GTCAATTCTATCATATTTTTTTGACAGCCTGCGACGCAAGCCCTCTAGCAAGATGCAGGC 60
G. lamblia P15      GTCAATTCTATCGTATTTTTTTGATAGCCTGAGACGCAAGCCCTCTAGCAAGATGCAGGC 60
G. lamblia GS       GTAAATTCTATAGCATTTCTTTGACAGCCTGTGACGCAAGCCCTCCAGCAAGGTGCAGAC 60
                    ** ********   **** ***** ****** ************* ****** ***** *

G. lamblia WB       CGGAGCCTGTGTCTCGTTCCCTGGGGCGATAGCTCTTGCTGGCAGGTCTTGCAGTGTCCA 120
G. lamblia P15      CGGAGTCTGTGCCTCGTTCCCTGGGGCGATAGCTCTTGCTGGCAGGTCTTGCAGTGTCCA 120
G. lamblia GS       CGGAGTCTGTGCCTCGTTCCCTGGGGCGATAGCTACTGCTGGAGGGTTTTGCATTATCCG 120
                    ***** ***** **********************  ******  *** ***** * ***

G. lamblia WB       TACCCGGGCAACACGTTTTCCAGCTACACCTTTACTCAACGTGCAC 166
G. lamblia P15      TGCTTGGGCAACACGTTCTCCAGCTATACCTTTACTCAACGTGTAT 166
G. lamblia GS       TATCTGGATAACACGTCCCCCAGCTATACCTTTACTCAAAGTGCGC 166
                    *    **  *******   ******* ************ ***
```

23. GlsR23

Genome	Sequence	Start	End	Strand	#Nucleotides
G. lamblia WB	GLCHR04	1890711	1890876	-	166
G. lamblia GS	ACGJ01001044	23638	23804	-	167
G. lamblia P15	contig54	22832	22998	+	167

```
AssemblageA         GACTTAGATACTGACATACTGCAGCTG-CGATAAAAAA-TCGTCTGGCCGCCTCGTGCCT 58
G. lamblia P15      GTCCTGGATACTGACA-ACCGCGGCTGGTAACAAAAAAATCGCCTTGTCGTCTCATGCCT 59
G. lamblia GS       GATCTGAGTGTTGGTATGCTGCAAAGGACGATAAAAAA-TCGCCATATGGTCTCATGCCT 59
                    *   *   *  **  *  * **    *   * ****** *** *     * *** *****

G. lamblia WB       ACGATGGGCTAGGGAAATGCCGTGACGAGACACGCACTGGGTGGCCATTGCGTCTGCGGT 118
G. lamblia P15      ACGATGGGCTAAAGAAGCACTGTGGCGAAACATGCACTGGGTGGCCATTGCGTCTGCGGT 119
G. lamblia GS       ACGATGGGCCAGAGGAGCATTTTGGCAAGACACACACTGGGCGGCCATTGCGTCTGCGGT 119
                    ********* *  * *      ** * * ***  ******* ******************

G. lamblia WB       AGATCCGCCGATTCCACAGCCCAGAAACACCCTTTACTCAAGCTGGCT 166
G. lamblia P15      AGACCCGCCGATTCCACAGCCCAGAAACATCCTTTACTTAAGCTGGCT 167
G. lamblia GS       AGACCCGCCGATTCCACAACCCAGGAACAGCCTTTACTCAAACCGATT 167
                    *** ************** ***** **** ******** ** * *  *
```

24. GlsR24

Genome	Sequence	Start	End	Strand	#Nucleotides
G. lamblia WB	GLCHR05	1301238	1301403	+	166
G. lamblia GS	ACGJ01002360	10351	10498	-	120
G. lamblia P15	contig463	79	259	-	181

```
G. lamblia WB       -----------------ACCGCCTGTAAACGCCTTCCCCGAATAATTTCGGG-CAGTCCT 42
G. lamblia P15      ACCAGCGCCTCTGAGCCCCCGCCTGTAAACGCCTTCCCCAAAA---TTCGGG-CAGTCCT 56
G. lamblia GS       -----------------------------------CTCCAATTTATTTCGGGGCAGTTCT 25
                                                       * ** *     ****** **** **

G. lamblia WB       TGCCCGCGGAGGGCACTTAAGGCTCCGGGGCCCGGGGCAGAGTCGGCCCTCCCAGAGCCC 102
G. lamblia P15      TGCCCGCGGAGGATCCTTAATGCCCCGGGGCCCGGGGCAGAGTCGGCCCTCCCAGAGCCC 116
G. lamblia GS       TGCCCGGGGAGGATACTTAATGCTCGGGTCCGCGCAGCAGAGTCAGTCCTCCCAGAGCCC 85
                    ****** *****   ***** ** * **  * **  ******** * *************

G. lamblia WB       GCCGACGCCCCCGAGCGCCAGCCCGGCGCAGGGGCCCGGCCACACTCATTTATTAAAC-A 161
G. lamblia P15      GCCGGCGCCCCCGAGCGTCAGCCCGGCGCAGGGGGCCGGCCACACTCATTTATTAAACCA 176
G. lamblia GS       GCCGGGACCCCCATGCGTCTGGCAGGCGCGGGGG-CCGGCCACATTCATTTATTAAAC-G 143
                    ****   *****  *** * * * ***** **** ********* *************
```

25. GlsR25

Genome	Sequence	Start	End	Strand	#Nucleotides
G. lamblia WB	GLCHR05	1459333	1459498	+	166
G. lamblia GS	ACGJ01001903	4369	4535	+	167
G. lamblia P15	contig778	18935	19100	+	166

```
G. lamblia WB     CTTTGCAACGGCACTTGTAACGAAAAAGTAAATCGAGGCTGCTAAAACACAGGGCTGCAC 60
G. lamblia P15    CTTTGCAACGGCACTTGCAACGAAAAAGTAAATTGAGGCTGCTAAAACACAGGGCTGCGC 60
G. lamblia GS     CTTTGCAACGGCATTTATAACGAAAAAGTAAATTGAGGCTGCTAAAACACAGGGCTGCAC 60
                  ************* **  *************** ************************ *

G. lamblia WB     AGCATCCTTGCACCTGCGTAGCCGATAGGTACGGGTGACCGTTTATCCCGGGCTCGTGTG 120
G. lamblia P15    AGCATCCTTGCACCTGCGCAGCCGACAGGTACGGGTGACCGTTTATCCCGGGCCCACGTG 120
G. lamblia GS     AGCATCCTTGCACCTGCGTAGCCGATAGACGCATTTGGCTGTTTATCTAGGATCTGCGTG 120
                  ****************** ****** **   *   ** * *******  **      ***

G. lamblia WB     GGCCCGGGTAGGCACGGTCAAAGAGTTTCCTTCATTCAA-TTTTTAG 166
G. lamblia P15    GATCCGAGCAGGCACGGTCAAAGACTTTCCTTCATTCAA-TTTTTAG 166
G. lamblia GS     GGTCTGAACAGGCACGGTCAAACAGTTTCCTTTACTCAAGTTTTTAG 167
                  *  * *   ************* * ******* * **** *******
```

26. Candidate-16 [as per reference (27)]

Genome	Sequence	Start	End	Strand	#Nucleotides
G. lamblia WB	GLCHR05	1006432	1006597	-	166
G. lamblia GS	ACGJ01001794	6880	7044	-	165
G. lamblia P15	contig399	33225	33390	+	166

```
G. lamblia WB     TGGATTGCTTCTTAAAAGATGGCCG-GAAGAGAAAAAGATCAAAAGCAAGGCTAGAGCCA 59
G. lamblia P15    TGGATTGCTTTTTAAAAAATGGCTG-GAGGAGAAAAAGATCAAAAGCAAGGCTAGAGCCA 59
G. lamblia GS     TGGATAGCTTTTTAAA-GATGGCTACAAAGAGAAAAAGATCAAAAGCAAGGCTAAAGCCA 59
                  ***** **** *****  *****    * ************************* *****

G. lamblia WB     TGGAGCGCGGATCTGCGCTCTGCCAGATACGCCGACAGAAAGCACCAAGGAAGGATGTGG 119
G. lamblia P15    TGGAGCGCAGACCTGCGCTCTGCCAGATACGCCGATAGGAAGCGCCAAGGAAGGACGCGG 119
G. lamblia GS     TGGAGCGCGGATTCGCGTTCTGCCAGATACGCCGATAGAAAGCAACAAGGAAGGACGTGG 119
                  ******** **   *** ***************** ** ****  ********** * **

G. lamblia WB     ATCTCCATGTCTGCCGTGTGCGCGCATATCCTTTACTCAATCTGTGT 166
G. lamblia P15    GCCTCCGTGTCTGCCGTGTGCGCGCATATCCTTTACTCAATCTGTGT 166
G. lamblia GS     -TTTCCATGTCTGCCATGTGCGCGCATATCCTTTAATCAATTTACGT 165
                     *** ******** ******************* ***** *  **
```

27. GlsR26

Genome	Sequence	Start	End	Strand	#Nucleotides
G. lamblia WB	GLCHR05	1459471	1459636	+	166
G. lamblia GS	ACGJ01001903	4507	4674	+	168
G. lamblia P15	contig778	19073	19238	+	166

```
G. lamblia WB     CAAAGAGTTTCCTTCATTCAA-TTTTTAGATCCATCTGGCGCACGTTACGAGGCCTGGCT 59
G. lamblia P15    CAAAGACTTTCCTTCATTCAA-TTTTTAGATCCATCTGGCGCACGTTACGAGGCCTGGCT 59
G. lamblia GS     CAAACAGTTTCCTTTACTCAAGTTTTTAGATCCATCTGGCGCACGTTACGAGGCTATGCT 60
                  **** * ******* * **** ********************************   ***

G. lamblia WB     CCCGGTCCGGCCCTACCTTGCGTGCGCATATCTCCGGGATCTGCGCCGCGTCTGCTCGCG 119
G. lamblia P15    TCCGGTCCGGCCCTACCTTGCGTGCGCATAGCTCCGGGATATGCGCCGTGTCTGCTCACG 119
G. lamblia GS     TCCGGCCTGGCCCTACCTTGCGTGCGCATAGCTCCGGGACATACACTACGTCTGGTCGTG 120
                   **** * ********************** ********  * * *   ***** **  *

G. lamblia WB     GCGATTTCGGTTATGCCGGCCCGAACA---CTCCTTCATTCAACAGGCCC 166
G. lamblia P15    GCGATTTCGGCTATGCCGGCCCGAACA---CTCCTTCGTTCAACAGGCTC 166
G. lamblia GS     ACGACTGCGACTGTACTGACCCGAACATTGCTCCTTCATTCAACAAAC-- 168
                   *** * **  * * * * ********   ******* *******  *
```

28. GlsR27

Genome	Sequence	Start	End	Strand	#Nucleotides
G. lamblia WB	GLCHR01	1371240	1371405	+	166
G. lamblia GS	ACGJ01002332	21871	22119	-	249
G. lamblia P15	contig9	61259	61425	-	167

```
G. lamblia WB     GGGCGAGATAT--------------------------CTT-------------------- 14
G. lamblia P15    GGGCGAGATAT--------------------------CTC-------------------- 14
G. lamblia GS     GAGCGAGAGATTGTACGGTATAGTTTGAGTAAAATCCCTTAACAGGAAATTTGCTTAGCC 60
                  * ****** **                          **

G. lamblia WB     -----TCA-------------------------------GCATTTATAAC-CA-AAAAAT 36
G. lamblia P15    -----TCA-------------------------------GTATCTATGGCTCA-AAAAAT 37
G. lamblia GS     AGTAATCAATGGGTCATTGCATTAAGTCAAGTAGGTGCTGTATTTACAAGCCACAAAAAT 120
                       ***                               * ** **     ** ******

G. lamblia WB     TAAGCTCACCCAAAGTCAACGGAGCGCCAGCTACGTGTTATGGGCAGCGAAAGTACCAGA 96
G. lamblia P15    TAAGCTCACCCAAAGTCAACGGAGCGTCAGCTACGTGTTATGGGCAGCGAAAGTACCAGA 97
G. lamblia GS     TAAGCTCACCCAAAGTCAACGAAACGCTAGCTTCGTGTTATGGGCAGCGAAAGTGCCAGA 180
                  ********************* * **  **** ********************* *****

G. lamblia WB     GCCAAAGAGTTCCTCTGATCGCCTGGCCGGAGCACATTTGTGATCTCCTATACCTTCATT 156
G. lamblia P15    GCCAAAGAGTTCCTCTGATCGCCTGGTCGGAGCACATCTGTGATCTCCTACACCTTCACT 157
G. lamblia GS     GCCAAAGAGTTCCTCTGATCGCCTGGCTGGAGCACACATGTGACCTCCTACACCTTTATT 240
                  **************************  ********  ***** ****** ***** * *

G. lamblia WB     TAATTAGCGT 166
G. lamblia P15    TAATTAGCCT 167
G. lamblia GS     TAAC-GGCCT 249
                  ***   ** *
```

Spliceosomal snRNA Candidates and RNase MRP RNA

29. Gl U1 snRNA

Genome	Sequence	Start	End	Strand	#Nucleotides
G. lamblia WB	GLCHR03	661194	661359	-	166
G. lamblia GS	ACGJ01002471	10962	11125	+	164
G. lamblia P15	contig621	4079	4244	+	166

```
G. lamblia WB     TGAGCAGGTCAAAAATTGAAGGTAATTTTAACTTACCTCAAGGGTGGCGACGAGCCAGTG 60
G. lamblia P15    TGAGCAGGCTAAAAATTGGAGGTAATTTTAACTTACCTCAAGGGTGGCAACGAGCCAGTG 60
G. lamblia GS     TGAGCAGGTCAAAA-TTGAAGACAATTTTAACTTACCTTAAGGGTGGCGATGAGCCATTG 59
                  ********  **** *** **  *************** ********* * ****** **

G. lamblia WB     TTCGGGCCAGGCTGGTGCTGCGCATACCGCGCTGGCACTGGTCACGGGGCAGTGCTCTCA 120
G. lamblia P15    TTCGGGCCAGGCTGGTGTTGCGCATACCGCGCTAGCACCGGTCACGGGGCAGTGCTCTCA 120
G. lamblia GS     TTCAGGCC-GGCTAATGCTGCGCATACCGCGCTGGTGTTTGTCACAGAGCAGTGCTCTCA 118
                  *** **** ****  ** *************** *     ***** * ************

G. lamblia WB     GACCTGCTACCGTACCCTTTTAATTTTTCCTTCACTTAAGGCCCAT 166
G. lamblia P15    GACCTGTTACCGTACCCTTTTAATTTTTCCTTCACTTAAGGCCCAT 166
G. lamblia GS     GACCTGCTACTGTACCCTTTTAATTTTTCCTTCGCTCAAGGCCTAT 164
                  ****** *** *********************  ** ****** **
```

30. Gl U2 snRNA

Genome	Sequence	Start	End	Strand	#Nucleotides
G. lamblia WB	GLCHR04	581396	581561	-	166
G. lamblia GS	ACGJ01002726	93831	93991	+	161
G. lamblia P15	contig698	112750	112915	+	166

```
G. lamblia WB     AGGCAAAAATTAAAATCAGAGTCGGCTTCGACTTTAGTGTAGTTACTGTTTCGTCGGCTT 60
G. lamblia P15    AGGCAAAAATTAAAATCAGAGTCGGCTTCGACTTTAGTGTAGTTACTGTTTTATCGGCTT 60
G. lamblia GS     -----GTAATTAAAACCAGAGTCGGCTTCGACTTTAGTGTAGTTACTGTTTTGTCGGCTT 55
                         ******** ***********************************  *******

G. lamblia WB     AACCGCCGATCCACTACATGCAAGGGGCAGCCGGGCTGTGAGGCAGCTGCCAGGATGGTC 120
G. lamblia P15    AGCCGCCGATCCACTGCATGCAAGGGGCAGTCGGGCTGTGAGGCAGCTGCCAGGATGGTC 120
G. lamblia GS     AACCGCCGATCCATTACATTCAAGGGGCAGTCGGGCTGTGAGGCAGCTGCCAGGATGGTC 115
                  * *********** * *** ********** *****************************

G. lamblia WB     CTGCCCTTGTCCCGGCTGGCGCCGTCCACCTTTATTCAAGTTTTCT 166
G. lamblia P15    CTGCCCTTGTCCCGGCTGGCGCCGTCCACCTTTATTCAAGCTTTCT 166
G. lamblia GS     CTACCCTTGTTCCGGCTGGCGCCGTCCACCTTTACTCAAGCTTTCT 161
                  ** ******* *********************** ***** *****
```

31. Gl U4 snRNA

Genome	Sequence	Start	End	Strand	#Nucleotides
G. lamblia WB	GLCHR02	1194980	1195145	+	166
G. lamblia GS	ACGJ01002266	10833	10998	-	166
G. lamblia P15	contig371	58049	58214	-	166

```
G. lamblia WB      TGTAAAATAAACTATTTTAAATTCAATTCTGTAAAATAAATTTTTTATTTTTTGACTCTA 60
G. lamblia P15     TGTAAAATTAACCATTTTAAATTCAATTTTGTAAAAATCAGTTTTTGTTTTTTGACTCTA 60
G. lamblia GS      TGTAGAATAAACTATTTTAAATTCAATTCTATTAAACTAATTTCTTTTTTCATGACTCTA 60
                   **** *** *** *************** * * ***   * ** ** ***  ********

G. lamblia WB      GGCTGAAGCTGCCAAGGTGCGTGATCCCTCGGTGATGCCTTGAGTGTTGCTTCACCAAAG 120
G. lamblia P15     GGCTGAAGCTGCCAAGGTGCGTGATCCCTCGGTGATGCCTTGAGTGTTGCTTCACCAAAG 120
G. lamblia GS      GACCGAAGCTGCCAAGGTGCGTGATCCCTCGGTGATGCCTTGAGTGTTGCTTCGCCAAAA 120
                   * * ************************************************* *****

G. lamblia WB      AACAACCACACGGCACAGCCGAATCTCTCATTTTTTAAACTTTCTC 166
G. lamblia P15     AACAACCACACGGCACAGCCGAATCTCTCATTTTTTAAACTCTTCT 166
G. lamblia GS      AACAACCACACGGCCTAGCCGAATTTCTCATTTTTTAAATCTCTCC 166
                   **************  ******** **************
```

32. Gl U6 snRNA

Genome	Sequence	Start	End	Strand	#Nucleotides
G. lamblia WB	GLCHR04	1813414	1813579	-	166
G. lamblia GS	ACGJ01001509	9049	9214	+	166
G. lamblia P15	contig24	136825	136989	-	165

```
G. lamblia WB      TAAACCATTTTAAATTGAAATAGGCGGTTGGAAATAAAAGCGCGGCGTGGTTAACAAAAA 60
G. lamblia P15     TAAACTATTTTAAATTGAAATAGATGATTAGAAATAAAAGCGTAGCGTGGTTAACAAAAA 60
G. lamblia GS      TAAACAATTTTAAATTAAAATCTACAATTCAGAATAAAAATGGAGCGTGGTTAACAAAAA 60
                   ***** ********** ****      **   *******  *  ****************

G. lamblia WB      CAGAGACAGTTAGCACCAGCTTCAGTCTAGAGTCGCTGGGGGACCTCTGGTTTCGCGGGA 120
G. lamblia P15     CAGAGACAGTTAGCACCAGCTTCAGTCTAGAGTCGCTGGGAGACCTCTGGTTTCGCGGGA 120
G. lamblia GS      CAGAGACAGTTAGCACCAGCTTCGGTCTAGAGTCGCTGGGGAACCTCTGGTTTCGCGGGA 120
                   *********************** ****************  ******************

G. lamblia WB      GCCCGTTGGCGCGTGCTTGCACCCCGCTCCTTTTCTCAATCTTCGC 166
G. lamblia P15     GCCCGTTGGCGCGTGCTTGCACCCCGCTCCTTTTCTCAATCCT-GC 165
G. lamblia GS      GCCTGTTGGCGCGTGCTTGCACCCCGCTCCTTTTCTCAATTGCTGC 166
                   *** ************************************    **
```

33. RNase MRP RNA

Genome	Sequence	Start	End	Strand	#Nucleotides
G. lamblia WB	GLCHR01	479835	480000	+	166
G. lamblia GS	ACGJ01002287	25852	26017	+	166
G. lamblia P15	contig818	88174	88339	-	166

```
G. lamblia WB      TCCCTGGGCGTCGGGCAGAAAGTGCCGGTCCTCTGGATTCCGGGGAGTGTCTGGTGCCGA 60
G. lamblia P15     TCCCTGGGCGTTGGGCAGAAAGTGCCGGTCCTCTGGACTCCGGGGAGTGTCTGGTGCCGA 60
G. lamblia GS      TCCCTGGGCGTTGGGCAGAAAGTGCCAGTCCTCTGGACTCCGGGGGGTGTCTGGTGCTAA 60
                   *********** ************** ********** ******* ***********  *

G. lamblia WB      TCGGACACTCCCTAGCCGCCACACTGACAGTTATGGTTGCAGGACAAGCTTAGCGAGTCC 120
G. lamblia P15     TCGGACACTCCCCAGCCGCCACACTGACAGTTATGGTTGCAGGACGAGCTTGGCGAGTCC 120
G. lamblia GS      TCGGACACCCCCTAGCCGCCACACTGACAGTTATGGTTGCAAGACGAGCTTAGCGAGTCT 120
                   ******** *** **************************** *** ***** *******

G. lamblia WB      GAACTCGACAGGGATACTCTACAGCGTTCCTTTATTCAATCATTGA 166
G. lamblia P15     GAACTCGATAGGGATACTCTACAGCGTTCCTTTATTCAATCGTTGG 166
G. lamblia GS      GAACTTGACAGGGATACTCTGCGGCGTTCCTTTATTAAATTGCGGG 166
                   ***** ** *********** * ************* ***    *
```

NcRNAs with no Assigned Function

34. Candidate-3 [as per reference (27)]

Genome	Sequence	Start	End	Strand	#Nucleotides
G. lamblia WB	GLCHR01	702197	702362	-	166
G. lamblia GS	ACGJ01002928	73869	74040	-	172
G. lamblia P15	contig780	40672	40838	+	167

```
G. lamblia WB      CCATTTCAGATAAAGTGGCGCATCTAGAACGGTCAAAAA-GGACCGATCGAAGACCAAGC 59
G. lamblia P15     CCATTTCAAATAAAATTGCATGTCTGGAGTGGCCAAAAA-GGACCGATCGAAGACCAAGC 59
G. lamblia GS      CCATTTCAGATGAAATTACACATCTGGAGCAACCAAAAAAGGATCGATCGAAGACCAGGC 60
                   ******** ** ** *  *   *** **      ****** *** ************* **

G. lamblia WB      GGTGCTAGGTTCAAGCCAGGTCCAAGACCCGGGCAGTCTGTGCTGTGGGGCGCCGCTGTA 119
G. lamblia P15     AGTGCTAGGTTCAAGCCGGGTCCAAGACCTGGGCAGTTTGTGCTGCGGGGCGTTGCTGTA 119
G. lamblia GS      AGTGCTAGGTTCAAACCGTATCCNAGAGTTTAGCAGCCTGTACCGTGGAGTGTTGATGTA 120
                    ************* **   *** ***     ****  *** * * ** * *  * ****

G. lamblia WB      GACGTCTTCCGAACACACCTGCGATAA-A-CCCTTTATTTAAA---AGATTA 166
G. lamblia P15     GACGTCTTTCGAACACACCTGCAACAA-AACCCTTTATTTAAA---AGGTTA 167
G. lamblia GS      GACGTCTTCTGAACACGGCTGCAACAATAATCCTTTATTTAAATCTAAGCTA 172
                   ********  ******  **** * ** *  ************   *   **
```

35. Candidate-5

Genome	Sequence	Start	End	Strand	#Nucleotides
G. lamblia WB	GLCHR03	299369	299534	+	166
G. lamblia GS	ACGJ01002279	13909	14070	-	162
G. lamblia P15	contig236	1707	1900	-	194

```
G. lamblia WB      GTGAAGGGAAGCAGACTCCATGGCATAAATAAATGCAAAATT---------------CTT 45
G. lamblia P15     GTGAAGGGAAGCGGACTCCATGACATAAATAAATGAAAAATTTCTTAATGTATAATTCTT 60
G. lamblia GS      GTGAAGTGAAGCAGACTCCATGGCATAAAAAA--GCAAATTT----------------CT 42
                   ****** ***** ********* ****** **  * *** **                 *

G. lamblia WB      TAACCTGAAAACA-------------AAATGGCTAGCAACACGAGGAAACGAGTGTTTCG 92
G. lamblia P15     TAATCTAAAAACATAACCCGGAAATAAAATGGCCAGCAACACGAGGGAACGAGTGTTCTG 120
G. lamblia GS      CAGTCC-AAAATA-------------AAATGGCTAAGAGCATGAGGAAACGACTGCTCTG 88
                    *  *  **** *             ******* *  * ** **** ***** ** *  *

G. lamblia WB      CCGGGCATAACTGGGCATGCATTTTCCTTGCCCAGTCTGCCTCCATACTAATTTCTCCTT 152
G. lamblia P15     CCGGGCATAACTGGGCATGCATTTTCCTTGCCCAGTCTGCCCTTGTATTAATTTCTCCTT 180
G. lamblia GS      CCGGGCATAACTGGGCATGCATTTTCCTTGCTCAGTCTGCCTCCTTACAAATTTCTCCTT 148
                   ******************************* *********    **  ***********

G. lamblia WB      TACTCAATCAGGAT 166
G. lamblia P15     TATTAAATCGAAGT 194
G. lamblia GS      TAATAAATCGAAGT 162
                   ** * ****    *
```

36. Candidate-12

Genome	Sequence	Start	End	Strand	#Nucleotides
G. lamblia WB	GLCHR03	474659	474824	-	166
G. lamblia GS	ACGJ01002930	21466	21613	-	148
G. lamblia P15	contig34	18717	18871	+	155

```
G. lamblia WB     TTCTTAGTCTCTTCTTAGCATCCAGAATAAATCACATTAAATGTATTTTAATTTGAATTT 60
G. lamblia P15    -----------TTCTTAGTATCCAGAATAAATTAAATTAAATGTATTTTAATTTGACTTT 49
G. lamblia GS     -----------TTCTCACTCACCAGAGTAAATTACAC-AAATGTATTTTGATTTGACTTC 48
                             **** *    ***** ***** * *  *********** ****** **

G. lamblia WB     TGATCCCCCGAGAAAAAGAACCCCAACCCGATGACGAATAGCTGTCCTGGCGGAGGCGGT 120
G. lamblia P15    TGATCCTTCAAGAAAAAGGGTCCCAACCCGATGACGAGTAGCTGTCCTGGCGGAGGCGGT 109
G. lamblia GS     CGGTATCCCGAGAAAAGAAAGCCCAACCCGATGACGAATAGCTGTCCTGGCGGAGGCGGT 108
                   * *    * ******     **************** **********************

G. lamblia WB     CATGACGACGAAGCCAT-CACGTAGGATCCCTTCACTCAACCTCTGC 166
G. lamblia P15    CATGACGACGATGCCAT-TACGTAGGATTCCTTCACTCAACCTTTGC 155
G. lamblia GS     CATGACGACAATGCCATATGCGTAGGTTCCCTTTACTCAA------- 148
                  ********* * *****   ****** * **** ******
```

37. Candidate-15

Genome	Sequence	Start	End	Strand	#Nucleotides
G. lamblia WB	GLCHR02	350512	350677	+	166
G. lamblia GS	ACGJ01002258	38250	38415	+	166
G. lamblia P15	contig571	3380	3545	-	166

```
G. lamblia GS     CGGCCTCTGGCTTGGACCCCGTGGCGCTGTCGGCCCCCGCGGAGGCAGGGGTTGGCTCGT 60
G. lamblia P15    TGGCCTCTGGCTTGGACCCCGTGGCGTTGTCGGCCCCCGCGGAGGCAGGGGCCGGCCCGC 60
G. lamblia WB     CGGCCTCTGGCTTGGACCCCGTGGCGTCGCCGGCCTCCGCGGAGGCAGGGGCCGGCCCGC 60
                   *************************  * ***** ***************  *** **

G. lamblia GS     CTTCAACTCAGCTGGACAGCCGGAGGCCGGAGACGGAGCACGGTCAGGCGGGCGGGGTGC 120
G. lamblia P15    CTTCAACTCAGCTGGACAGCCGGAGGCCGGAGACGGAGCACGGTCAGGCGGGCGGGGTGC 120
G. lamblia WB     CTTCAACTCAGCCGGACAGCCGGAGGCCGGAGACGGAGCACGGTCAGGCGGGCGGGGTGC 120
                  ************ ***********************************************

G. lamblia GS     AGTGCCAGCCTCAGTCGCAGAGCGGCTTCCTTTACTCAAGATCGGG 166
G. lamblia P15    AGTGCCAGCCTCAGTCGCAGAGCGGCTTCCTTTACTCAAGATCGGG 166
G. lamblia WB     AGTGCCAGCCCCAGCCGCAGAGCGGCTTCCTTTACTCAAGATCGGG 166
                  ********** *** *******************************
```

38. Candidate-17

Genome	Sequence	Start	End	Strand	#Nucleotides
G. lamblia WB	GLCHR03	1601283	1601448	-	166
G. lamblia GS	ACGJ01002096	4585	4750	-	166
G. lamblia P15	contig753	29559	29724	-	166

```
G. lamblia WB     GAGTTAATACCACCAAACCCCTGTGCGTACATGTCGCCCCCTAACCTTCTGATGCGGATA 60
G. lamblia GS     GAGTTAATACCACCAAACCCCTGTGCGTACATGTCGCCCCCTAACCTTCTGATGCGGATA 60
G. lamblia P15    GAGTTAACACCACCAAACCCCTGTGCGTACATGTCGCCCCCTAACCTTCTGATGCGGATA 60
                  ******* ****************************************************

G. lamblia WB     CCTTGCCGCAGGGCCGTTAAGCGAGGCTTGGCCCGTGCGACGATGAGGCTCCCTGCGGGG 120
G. lamblia GS     CCTTGCCGCAGGGCCGTTAAGCGAGGCTTGGCCCGTGCGACGATGAGGCTCCCCACGGGG 120
G. lamblia P15    TCTTGCCGCAGGGCCGTTAAGCGAGGCTTGGCCTGTGCGACGATGAGGCTCCCTGCGGGG 120
                   ******************************** *******************  *****

G. lamblia WB     AAGCCCTGCGGCGCGTCTTAAGGAGGCTCCTTCACTCAACGGCGTC 166
G. lamblia GS     AAGCCCTGCGGCGCGTCTTAAGGAGGCTCCTTCACTCAACGGCGTC 166
G. lamblia P15    AAGCCCTGCGGCTCGTCTTAAGGAGGCTCCTTCACTCAATGGCGTC 166
                  ************ ************************** ******
```

39. Candidate-21

Genome	Sequence	Start	End	Strand	#Nucleotides
G. lamblia WB	GLCHR05	1896857	1897022	-	166
G. lamblia GS	ACGJ01002272	33335	33498	+	164
G. lamblia P15	contig632	33226	33399	+	174

```
G. lamblia WB       CGCCAGCTATTCTACGTCTGTGGCCGTTCTGGCTGCGCTGGACGATGAACTGGAGATGCT 60
G. lamblia P15      CACCAGCTATTCTACGTCTGTGGCCGTTCTGGCTGCGCTAGGCGATGAACTGGAGATGTT 60
G. lamblia GS       CACCAGCTATTCTACGCCTGTGGCCGTTCTGGTTGCGCTGGGTGTTGAACAGGTGATG-T 59
                    * ************** *************** ****** *  * ***** ** **** *

G. lamblia WB       GGACACGGCTTTGCTCTCCCACCGGAGCACATATGCTGCAGGATGACCGGCGCCTGTCTC 120
G. lamblia P15      GGACACGGCTTTGCTCTCCCACCGGAGAACATATGCTGCAGAACGGACGGCACCTGTCCC 120
G. lamblia GS       GTACACGACTCTGCTCTCCCACTGGAGTACACCTGCTGTAGAACGGACGGTACCTGTCCC 119
                    * ***** ** *********** **** ***  ***** ** * *  ***  ****** *

G. lamblia WB       CCACCACGTGCCAGCTAAACTGCAGCC--------ACATTTATTCAACCTTTTC 166
G. lamblia P15      CCACCATGTGCCAGATAAACTGCAGCAACATTACAACATTTATTCAACCTCTTC 174
G. lamblia GS       CCACTGTGTGCCAGCTAAATTACAGCA--------ACATTTACTCAACCCTTC- 164
                    ****   ******* **** * **** *****   ******* ******  *
```

40. GlsR28

Genome	Sequence	Start	End	Strand	#Nucleotides
G. lamblia WB	GLCHR01	978028	978193	-	166
G. lamblia GS	ACGJ01002347	4321	4497	-	177
G. lamblia P15	contig59	153500	153665	+	166

```
G. lamblia WB       AAACTGCACCCTTGTGTTACTC-TGGTGTGTTCTTTATTACCCTACTCTGTCTCGTGAAC 59
G. lamblia P15      AAACTGCACCCTTGCGTTACTC-CGGTGTGTTCTTTATTACCCTACTCTGTCTCGTGAAT 59
G. lamblia GS       AAACTGCACCCTTGCGTCACCCCTGGTGTGTTCTTTATTACCCTACTCTGTCTAGTGATC 60
                    ************** ** ** *  ***************************** ****

G. lamblia WB       CCTCACCACCAGTTCTTCTCGCAGTGGTCTCTGTCAAAGACTGCCGCACGGTACACCAGA 119
G. lamblia P15      TCTCACCACCAGTTCTTCTCGCAGTGGTCTCTGTCAAAGACTGCCGCATGGTACACCGGA 119
G. lamblia GS       TCTCACCACCAGTTCTTCTCGCAGTGGTCTCTGTCAAAGACTGCCGCATGGTACACCAGA 120
                     *********************************************** ******** **

G. lamblia WB       AGCAAGGGGAAGGATCCCATCCACGCAGTCCTTTACTTAAACA--------TGGG-- 166
G. lamblia P15      AGCAAGGGGAAGGATCCCATCCACGCAGTCCTTTACTTAAACG--------CGGG-- 166
G. lamblia GS       AGCAAGGGAAAGGAACCCATCCACGCAGTCCTTTACTTAAATAAGACCCTACGGGGG 177
                    ******** ***** **************************           ***
```

Trans-spliced Intron 5' Halves

41. Hsp90 Exon 1-Intron 5' half

Genome	Sequence	Start	End	Strand	#Nucleotides
G. lamblia WB	GLCHR05	2515245	2515410	+	166
G. lamblia GS	ACGJ01002286	15902	16067	-	166
G. lamblia P15	contig421	16140	16305	-	166

```
G. lamblia WB      TCGACGGCGCCGCCCAGTTCCGCGGGATCCTCTTCATCCCCAAGCGCGCGCCCTTCGACA 60
G. lamblia P15     TAGACGGCGCCGCTCAGTTCCGCGGTATCCTCTTCATCCCCAAGCGCGCGCCCTTCGACA 60
G. lamblia GS      TCGATGGCGCCGCACAGTTCCGCGGCATCCTCTTCATCCCTAAGCGCGCGCCCTTCGACA 60
                   * ** ******** *********** ************** *******************

G. lamblia WB      TGTGGGACGCTCAGAAGAAGAAGACGGGCATCAAGCTCATGGTCAAGAAAGTGTATGTT- 119
G. lamblia P15     TGTGGGACGCTCAAAAGAAGAAGACGGGCATCAAGCTCATGGTCAAGAAAGTGTATGTT- 119
G. lamblia GS      TGTGGGACGCTCAGAAGAAGAAGACGGGCATCAAGCTCATGGTCAAGAAAGTGTATGTTT 120
                   ************* *********************************************

G. lamblia WB      ATGTTTGTATGCTGTATGTGTGCGAGACTCCTTTACTCAAATTTGCG 166
G. lamblia P15     ATGTTTGTATGCTGTATGTGTGCGAGACTCCTTTACTCAAATTCGCG 166
G. lamblia GS      ATGTATGTGTGTTGTATGTGTGCGAGACTCCTTTACTCAA-TTCGTG 166
                   **** *** ** **************************** ** * *
```

42. DHC β Exon 2-Intron 5' half

Genome	Sequence	Start	End	Strand	#Nucleotides
G. lamblia WB	GLCHR03	577578	577743	+	166
G. lamblia GS	ACGJ01002314	1045	1211	-	167
G. lamblia P15	contig30	114285	114449	-	165

```
G. lamblia WB      GCCCGTAAGAAATACAAAGCTGTCATGGACAACGTTGCAAAGCTGAACAAAAAGCTCCAA 60
G. lamblia P15     GCCCGTAAGAAGTACAAAGCTGTCATGGACAACGTTGCAAAGCTGAACAAGAAGCTTCAA 60
G. lamblia GS      GCCCGTAAGAAGTACAAAGCCGTCATGGACAACGTTTCGAAGCTCAACAAGAAGCTCCAA 60
                   *********** ******** *************** * ***** ***** ***** ***

G. lamblia WB      ACTCTCAAGGATCAATTTGACAAGGGTATGTTACTGGGTGAAACGCTACTTATGTATGTA 120
G. lamblia P15     ACTCTCAAGGATCAATTTGACAAGGGTATGTTACTAGGTGAAACGCTACTTATGTGTGTA 120
G. lamblia GS      ACTCTCAAAGACCAATTCGACAAGGGTATGTTACC-GGTGAAACGCTACTTATGTGTGTA 119
                   ******** ** ***** ****************  ******************* ****

G. lamblia WB      TGCTTATATGT-CTTCGCGCTCAGGCGCTCCTTTACTCAAT-TATCAG 166
G. lamblia P15     TGTCTATATGT-CT-CGCGCTCGGGCGCTCCTTTACTCAAT-TATCAA 165
G. lamblia GS      TGTCTATATGTTCTTCGCGCTTAGGCGCTCCTTTACTCAAAACACCAA 167
                   **  ******* ** ******  *****************   * **
```

43. DHC β Exon 3-Intron 5’ half

Genome	Sequence	Start	End	Strand	#Nucleotides
G. lamblia WB	GLCHR05	4266308	4266473	+	166
G. lamblia GS	ACGJ01002906	4407	4562	+	156
G. lamblia P15	contig39	71963	72134	+	172

```
G. lamblia WB      GTTTGCTCTGATCTTCTTCCATGCAATCGTCATCGAGAGACGGAAGTTCGGTCCTATAGG 60
G. lamblia P15     GTTTGCTCTGATTTTCTTCCATGCAATTGTCATCGAGAGACGAAAGTTCGGTCCTATAGG 60
G. lamblia GS      GTTTGCTTTAATATTCTTCCATGCAATCGTCATCGAGAGACGAAAATTCGGTCCCATAGG 60
                   ******* * ** ************** ************** ** ******** *****

G. lamblia WB      GTATGTTTGTAATCTGTGTAGTCGCAGTATGCCATTATTTTATAACGTGTATGTCATTAT 120
G. lamblia P15     GTATGTTCGTAATCTGTGTAGTTGCAGTATGCCATTGTTTTATAACGTGTATGTCACTAT 120
G. lamblia GS      GTATGTTCACG--CTGTGTAGT-GCAGTATGCCATT--TTTAAA-----TATGTTAGTAT 110
                   *******      ********* *************  **** *     ***** * ***

G. lamblia WB      GTCAGTATGCCAGTGCGCTGGTG------AGTTTCCTTTATTCAAATGTTGT 166
G. lamblia P15     GTCAGTATGTCAGTACGCCAGTACAAGTAATTTTCCTTTACTCAAATATTTT 172
G. lamblia GS      GTTAATATGTCAGTACGCTAGTAC------ATTTCCTTTACTCAAGTGCTAT 156
                   ** * **** **** ***  **         ********* **** *  * *
```

44. DHC γ Exon 1-Intron 5’ half

Genome	Sequence	Start	End	Strand	#Nucleotides
G. lamblia WB	GLCHR03	967465	967630	+	166
G. lamblia GS	ACGJ01002918	40974	41139	+	166
G. lamblia P15	contig38	36690	36855	-	166

```
G. lamblia GS      TCTTGCGGTATCTTCATCACGATGAACCCCGGTTACGCCGGGCGTCAAGAACTTCCAGAG 60
G. lamblia P15     TCTTGCGGTATCTTCATCACGATGAACCCCGGTTACGCCGGGCGTCAGGAACTTCCAGAG 60
G. lamblia WB      TCTTGCGGTATTTTCATCACGATGAACCCCGGTTACGCCGGGCGTCAAGAACTTCCAGAG 60
                   *********** *********************************** ************

G. lamblia GS      AATCTCAAAGCTTTATTCCGTAGTGTTGCAATGATATGTTTACAGGTGGTTCGGTGTGTA 120
G. lamblia P15     AATCTCAAAGCCTTATTCCGTAGCGTTGCAATGATATGTTTACAGGTGGTTCGGTGTGTA 120
G. lamblia WB      AATCTCAAAGCCTTATTCCGTAGCGTTGCAATGATATGTTCACAGGTGGTTTGGTGTGTA 120
                   *********** *********** **************** ********** ********

G. lamblia GS      TGCTTGGCGTGTATGTGTGTATGTCCTTCCTTTACTCAATGCCCAG 166
G. lamblia P15     TGCTTGGCGTGTATGTGTGTATGTCCTTCCTTTACTCAATGCTTGG 166
G. lamblia WB      TGCTTGGCGTGTATGTGTGTATGTTCCTCCTTTACTCAATACTTGG 166
                   ************************ * ************* *   *
```

Supplementary Figure 3 – Motif sequence variants in *Giardia* WB, P15 and GS isolates

Occurrences of variant motif sequences in ncRNA and *trans*-spliced intron containing genes in *G. lamblia* WB, P15 and GS isolates (132 total motif instances) identified in this study are annotated in descending order of observed frequency. The consensus 'TCCTTTACTCAA' motif sequence was observed 34 times and nucleotides differing in motif variants are highlighted in bold red text.

No.	Motif Variant	WB	P15	GS	Frequency
1	TCCTTTACTCAA	GlsR7<br>GlsR15<br>Candidate-1<br>Candidate-23<br>GlsR18<br>Candidate-16<br>Candidate-15<br>Hsp90 Exon 1<br>DHC β Exon 2<br>DHC γ Exon 1<br>Candidate-5	GlsR2<br>GlsR7<br>GlsR15<br>Candidate-1<br>Candidate-23<br>Candidate-16<br>Candidate-15<br>Hsp90 Exon 1<br>DHC β Exon 2<br>DHC β Exon 3<br>DHC γ Exon 1	GlsR2<br>GlsR5<br>GlsR7<br>GlsR15<br>GlsR18<br>GlsR20<br>GlsR25<br>Candidate-15<br>Hsp90 Exon 1<br>DHC β Exon 2<br>DHC β Exon 3<br>DHC γ Exon 1	34
2	TCCTTTACT**T**AA	GlsR2<br>Candidate-13<br>GlsR20<br>GlsR28	Candidate-13<br>GlsR20<br>GlsR23<br>GlsR28	GlsR28	9
3	TCCTT**C**ACTCAA	GlsR5<br>GlsR6<br>Candidate-17	GlsR6<br>Candidate-12<br>Candidate-17	GlsR6<br>GlsR8<br>Candidate-17	9
4	**A**CCTTTACTCAA	GlsR22<br>GlsR24	GlsR22<br>GlsR24	GlsR22<br>GlsR24<br>Gl U2 snRNA	7
5	TCCTTT**T**CTCAA	GlsR10<br>Gl U6 snRNA	GlsR10<br>Gl U6 snRNA	Gl U6 snRNA	5
6	TCCTTTA**T**TCAA	RNase MRP<br>DHC β Exon 3	GlsR18<br>RNase MRP	Candidate-23	5
7	**A**C**A**TTTA**T**TCAA	GlsR21<br>Candidate-21	GlsR21<br>Candidate-21	GlsR21	5
8	TCCTTTA**A**T**A**AA	GlsR9	GlsR9	GlsR9<br>Candidate-5	4
9	**C**CCTTTACTCAA	GlsR23		GlsR13<br>Candidate-1<br>Candidate-12	4
10	TCCTT**C**A**T**TCAA	GlsR25<br>GlsR26	GlsR25	GlsR26	4
11	TCCTT**C**A**T**T**T**AA	GlsR1	GlsR1	GlsR1	3
12	**C**CCTT**C**ACTCAA	GlsR8<br>Candidate-12	GlsR8		3
13	TCCTT**C**A**AT**T**A**A	GlsR19	GlsR19	GlsR19	3
14	TC**A**TTT**TT**T**A**AA	Gl U4 snRNA	Gl U4 snRNA	Gl U4 snRNA	3
15	TC**A**TT**C**ACTCAA	GlsR4	GlsR4		2
16	**C**CCTTTACT**T**AA	GlsR13	GlsR13		2
17	**C**CCTTTA**T**TCAA			GlsR14<br>Candidate-13	2

Supplementary Figure 3 (continued)

No.	Motif Variant	WB	P15	GS	Frequency
18	TCCTTTGCTCAA	Candidate-2	Candidate-2		2
19	TCCTTTTCTAAA	GlsR17	GlsR17		2
20	TCCTTTATTAAA		Candidate-5	RNase MRP	2
21	CCCTTTATTTAA	Candidate-3	Candidate-3		2
22	TCCTTCACTTAA	Gl U1 snRNA	Gl U1 snRNA		2
23	ACCTTTATTCAA	Gl U2 snRNA	Gl U2 snRNA		2
24	TCATTTACTCAA			GlsR4	1
25	ACATTTACTCAA			Candidate-21	1
26	ACCTTCACTTAA		GlsR27		1
27	ACCTTCATTTAA	GlsR27			1
28	ACCTTTATTTAA			GlsR27	1
29	CCCTTTGCTCAA		GlsR14		1
30	GCCTTTACTCAA			GlsR23	1
31	TCCTTCGCTCAA			Gl U1 snRNA	1
32	TCCTTCGCTTAA			GlsR17	1
33	TCCTTCGTTCAA		GlsR26		1
34	TCCTTCTCTCAA			GlsR10	1
35	TCCTTTAATCAA			Candidate-16	1
36	TCCTTTACTAAA		GlsR5		1
37	TCCTTTATTTAA			Candidate-3	1
38	TCCTTTCCTCAA	GlsR14			1
39	TCCTTTTCTTAA			Candidate-2	1
**Total Motif Occurrences**					**132**

Supplementary Figure 4. RT-PCR detection of motif-containing ncRNA and *trans*-spliced intron precursor transcripts

RT-PCR product sequences (top) are compared with *Giardia* WB isolate genomic DNA sequences (bottom, Gl_WB). Genomic locations of amplified regions are given as well as the number of unique clones that were isolated and sequenced. Regions specifying ncRNA or protein coding sequences are indicated in red text with motif sequences highlighted in green. *Trans*-spliced intron 5' splice sites are underlined. Primer annealing sites are indicated with arrows that denote the direction of amplification during PCR.

1. GlsR17 and GlsR18 dicistronic transcript (GLCHR01:149338-149577) – 2 Clones

```
                                                 GlsR17
                     oAH74
RT-PCR       1 ATAATGCGCTTCTTTGAGCCGCGGGTTTACTCGTGGTGAGGATCCGGGGCACTGAGCAATCCCCAGGACACAGGCGGAGCGGAAGGCACGGCTGCGCCACGCAGCCTAATCACCGCCCCT 120
               ||||||||||||||||||||||||||||||||||||||||||||||||||||||||||||||||||||||||||||||||||||||||||||||||||||||||||||||||||||||||
Gl_WB   149338 ATAATGCGCTTCTTTGAGCCGCGGGTTTACTCGTGGTGAGGATCCGGGGCACTGAGCAATCCCCAGGACACAGGCGGAGCGGAAGGCACGGCTGCGCCACGCAGCCTAATCACCGCCCCT 149457

                                                          GlsR18
Query:     121 ATAGTCCTTTTCTAAACGCGTGGCCGGTGCAGCTGCCCGCTGGCGCTTGCGAGCGTGCACAGGCCTACATCCAGGGTCATAGGTGGGGAGCGGATCCCGTCCATCCTCAATCCGGGCCCG 240
               ||||||||||||||||||||||||||||||||||||||||||||||||||||||||||||||||||||||||||||||||||||||||||||||||||||||||||||||||||||||||
Gl_WB   149458 ATAGTCCTTTTCTAAACGCGTGGCCGGTGCAGCTGCCCGCTGGCGCTTGCGAGCGTGCACAGGCCTACATCCAGGGTCATAGGTGGGGAGCGGATCCCGTCCATCCTCAATCCGGGCCCG 149577
                                                                                                                    oAH73
```

2. GlsR25 and GlsR26 dicistronic transcript – 2 Clones

```
                                                     GlsR25
                    oAH75
RT-PCR        1 GAGGCTGCTAAAACACAGGGCTGCACAGCATCCTTGCACCTGCGTAGCCGATAGGTACGGGTGACCGTTTATCCCGGGCTCGTGTGGGCCCGGGTAGGCACGGTCAAAGAGTTTCCTTCA 120
                ||||||||||||||||||||||||||||||||||||||||||||||||||||||||||||||||||||||||||||||||||||||||||||||||||||||||||||||||||||||||
Gl_WB   1459367 GAGGCTGCTAAAACACAGGGCTGCACAGCATCCTTGCACCTGCGTAGCCGATAGGTACGGGTGACCGTTTATCCCGGGCTCGTGTGGGCCCGGGTAGGCACGGTCAAAGAGTTTCCTTCA 1459486

                                                          GlsR26
RT-PCR      121 TTCAATTTTTAGATCCATCTGGCGCACGTTACGAGGCCTGGCTCCCGGTCCGGCCCTACCTTGCGTGCGCATATCTCCGGGATCTGCGCCGCGTCTGCTCGCGGCGATTTCGGTTATGCCGGCC 244
                ||||||||||||||||||||||||||||||||||||||||||||||||||||||||||||||||||||||||||||||||||||||||||||||||||||||||||||||||||||||||||||
Gl_WB   1459487 TTCAATTTTTAGATCCATCTGGCGCACGTTACGAGGCCTGGCTCCCGGTCCGGCCCTACCTTGCGTGCGCATATCTCCGGGATCTGCGCCGCGTCTGCTCGCGGCGATTTCGGTTATGCCGGCC 1459610
                                                                                                                          oAH60
```

3. Candidate-23 and DNA polymerase dicistronic transcript (GLCHR01:450046-449887) - 2 Clones

```
                  Candidate-23
                     oAH77                                                      DNA Polymerase (GL50803_35094)
RT-PCR       1 GGCATGGAGAAGAGCAGACTTGAGGCCTGCTCCTTTACTCAATTTTGTGGCATCGCACACAACTTACTTTTCTGGCCAGATGGAGCAGTTCCTGCTAGGCCTTAGGAGGCCGTCTGTCGG 120
               ||||||||||||||||||||||||||||| ||||||||||||||||||||||||||||||||||||||||||||||||||||||||||||||||||||||||||||||||||||||||||
Gl_WB   450046 GGCATGGAGAAGAGCAGACTTGAGGCCTGTTCCTTTACTCAATTTTGTGGCATCGCACACAACTTACTTTTCTGGCCAGATGGAGCAGTTCCTGCTAGGCCTTAGGAGGCCGTCTGTCGG 449926

RT-PCR     121 GCCAGAGGTTTACGAGGAAGACGTGGAATTCCAGCTTAT 159
               |||||||||||||||||||||||||||||||||||||||
Gl_WB   449925 GCCAGAGGTTTACGAGGAAGACGTGGAATTCCAGCTTAT 449887
                                  oAH76
```

4. Candidate-5 and Serine/Threonine Kinase (GLCHR03: 299486-299755) – 2 Clones

Candidate-5

oAH79

```
RT-PCR       1 CCTTGCCCAGTCTGCCTCCATACTAATTTCTCCTTTACTCAATCAGGATGGCGAGCGGCAGTGTGAATGATGTGTCTTAGCAGAATTTTAACTAGGGGAAAATGGCCGTATCAGTCATCA 120
               ||||||||||||||||||||||||||||||||||||||||||||||||||||||||||||||||||||||||||||||||||||||||||||||||||||||||||||||||||||||||
Gl_WB   299486 CCTTGCCCAGTCTGCCTCCATACTAATTTCTCCTTTACTCAATCAGGATGGCGAGCGGCAGTGTGAATGATGTGTCTTAGCAGAATTTTAACTAGGGGAAAATGGCCGTATCAGTCATCA 299605
```

Serine/Threonine Kinase (GL50803_4329)

```
RT-PCR     121 CGACGGACGATTGGTTTAAGGCCATCCGTTGTTCTAAGTACCCTCTTGTCACAAAGTACATCCATGAGTTCAAGGGCAAGCGATGCTCTATGGAGTGGACGGCACTAATGTGGGCAGCAA 240
               ||||||||||||||||||||||||||||||||||||||||||||||||||||||||||||||||||||||||||||||||||||||||||||||||||||||||||||||||||||||||
Gl_WB   299606 CGACGGACGATTGGTTTAAGGCCATCCGTTGTTCTAAGTACCCTCTTGTCACAAAGTACATCCATGAGTTCAAGGGCAAGCGATGCTCTATGGAGTGGACGGCACTAATGTGGGCAGCAA 299725

RT-PCR     241 AATTTGGGGATGTGAAAATGGTCGAGCTCC 270
               ||||||||||||||||||||||||||||||
Gl_WB   299726 AATTTGGGGATGTGAAAATGGTCGAGCTCC 299755
```

oAH78

5. Hsp90 Exon-Intron 5' half and Replication Factor C Subunit 5 dicistronic transcript (GLCHR05:2515270-2515568) – 2 Clones

Hsp90 5' Exon

oAH2

```
RT-PCR        1 GATCCTCTTCATCCCCAAGCGCGCGCCCTTCGACATGTGGGACGCTCAGAAGAAGAAGACGGGCATCAAGCTCATGGTCAAGAAAGTGTATGTTATGTTTGTATGCTGTATGTGTGCGAG 120
                ||||||||||||||||||||||||||||||||||||||||||||||||||||||||||||||||||||||||||||||||||||||||||||||||||||||||||||||||||||||||
Gl_WB   2515270 GATCCTCTTCATCCCCAAGCGCGCGCCCTTCGACATGTGGGACGCTCAGAAGAAGAAGACGGGCATCAAGCTCATGGTCAAGAAAGTGTATGTTATGTTTGTATGCTGTATGTGTGCGAG 2515389
```

Replication Factor C Subunit 5 (GL50803_16127)

```
RT-PCR      121 ACTCCTTTACTCAAATTTGCGGCGTCGTGCTGCGGTCCTTCTCACGAAAATTCTTTGACCTCGCCTCGCCGGCATGTGGTGCAACAAGTACACCCCCACGCAGCTGGAGGCCATGGATTA 240
                ||||||||||||||||||||||||||||||||||||||||||||||||||||||||||||||||||||||||||||||||||||||||||||||||||||||||||||||||||||||||
Gl_WB   2515390 ACTCCTTTACTCAAATTTGCGGCGTCGTGCTGCGGTCCTTCTCACGAAAATTCTTTGACCTCGCCTCGCCGGCATGTGGTGCAACAAGTACACCCCCACGCAGCTGGAGGCCATGGATTA 2515509

RT-PCR      241 TCATTGCGACGCCACCAGGCTTTTCATCAGCGTCGCCCGGGCAAAAAATCCACCTCATC 299
                |||||||||||||||||||||||||||||||||||||||||||||||||||||||||||
Gl_WB   2515510 TCATTGCGACGCCACCAGGCTTTTCATCAGCGTCGCCCGGGCAAAAAATCCACCTCATC 2515568
```

oAH1

6. DHC Beta Exon 2-Intron 1 5' half (GLCHR03:577556-577881) – 2 Clones

DHC Beta Exon 2

oAH10

```
RT-PCR       1 GGCCGCGATGCAGCTCGAAGCTGCCCGTAAGAAATACAAAGCTGTCATGGACAACGTTGCAAAGCTGAACAAAAAGCTCCAAACTCTCAAGGATCAATTTGACAAGGGTATGTTACTGGG 120
               ||||||||||||||||||||||||||||||||||||||||||||||||||||||||||||||||||||||||||||||||||||||||||||||||||||||||||||||||||||||||
Gl_WB   577556 GGCCGCGATGCAGCTCGAAGCTGCCCGTAAGAAATACAAAGCTGTCATGGACAACGTTGCAAAGCTGAACAAAAAGCTCCAAACTCTCAAGGATCAATTTGACAAGGGTATGTTACTGGG 577675

RT-PCR     121 TGAAACGCTACTTATGTATGTATGCTTATATGTCTTCGCGCTCAGGCGCTCCTTTACTCAATTATCAGCAAATTCGTTTACAGGCCGCCGAGAAGTCAGACCACCGATAGCGTGGAACTC 240
               ||||||||||||||||||||||||||||||||||||||||||||||||||||||||||||||||||||||||||||||||||||||||||||||||||||||||||||||||||||||||
Gl_WB   577676 TGAAACGCTACTTATGTATGTATGCTTATATGTCTTCGCGCTCAGGCGCTCCTTTACTCAATTATCAGCAAATTCGTTTACAGGCCGCCGAGAAGTCAGACCACCGATAGCGTGGAACTC 577795
```

```
RT-PCR    241 AGGCAGCCGTTATCTTTGTAACCCCGTAGTTTGCCTCTAGGAATTGCAGAATCTCGTCTGTGAGGAAGAGAGGAAGAGAGGGGCCC 326
              ||||||||||||||||||||||||||||||||||||||||||||||||||||||||||||||||||||||||||||||||||||||
Gl_WB  577796 AGGCAGCCGTTATCTTTGTAACCCCGTAGTTTGCCTCTAGGAATTGCAGAATCTCGTCTGTGAGGAAGAGAGGAAGAGAGGGGCCC 577881
                                                                                     oAH9
```

7. DHC Beta Exon 3-Intron 2 5' half (GLCHR05:4266265-4266624) - 2 clones

```
                                           DHC Beta Exon 3
                 oAH14
RT-PCR       1 CGTTTGAAATGTGCTCCAAGGGTAACGAGTTCAAGAGTATTTTGTTTGCTCTGATCTTCTTCCATGCAATCGTCATCGAGAGACGGAAGTTCGGTCCTATAGGGTATGTTTGTAATCTGT 120
               ||||||||||||||||||||||||||||||||||||||||||||||||||||||||||||||||||||||||||||||||||||||||||||||||||||||||||||||||||||||||
Gl_WB  4266265 CGTTTGAAATGTGCTCCAAGGGTAACGAGTTCAAGAGTATTTTGTTTGCTCTGATCTTCTTCCATGCAATCGTCATCGAGAGACGGAAGTTCGGTCCTATAGGGTATGTTTGTAATCTGT 4266384

RT-PCR     121 GTAGTCGCAGTATGCCATTATTTTATAACGTGTATGTCATTATGTCAGTATGCCAGTGCGCTGGTGAGTTTCCTTTATTCAAATGTTGTGCAGTTGCTCTGTTCTATTCTATTCTGTTGG 240
               ||||||||||||||||||||||||||||||||||||||||||||||||||||||||||||||||||||||||||||||||||||||||||||||||||||||||||||||||||||||||
Gl_WB  4266385 GTAGTCGCAGTATGCCATTATTTTATAACGTGTATGTCATTATGTCAGTATGCCAGTGCGCTGGTGAGTTTCCTTTATTCAAATGTTGTGCAGTTGCTCTGTTCTATTCTATTCTGTTGG 4266504

RT-PCR     241 TTTTCGAGTATGGTGACTCATTCTGAGACTCTTACTTTAGCCAAGACTGCGTCCGGATCGCAGATAATGCACCGGCTGCAGTTTCCTTGTTTATATATGCCTACCAATCTATCTGCCAAC 360
               ||||||||||||||||||||||||||||||||||||||||||||||||||||||||||||||||||||||||||||||||||||||||||||||||||||||||||||||||||||||||
Gl_WB  4266505 TTTTCGAGTATGGTGACTCATTCTGAGACTCTTACTTTAGCCAAGACTGCGTCCGGATCGCAGATAATGCACCGGCTGCAGTTTCCTTGTTTATATATGCCTACCAATCTATCTGCCAAC 4266624
                                                                                                                          oAH13
```

Supplementary Figure 5. 5’ and 3’ RACE analysis of *Giardia* ncRNAs and *trans*-spliced introns

Giardia WB isolate genomic sequences are shown with sequencing results from 5’ and 3’ RACE experiments overlaid in red text and motif sequences highlighted in green. *Trans*-spliced intron 5’ exons are in grey and the 5’ splice sites are highlighted in blue. Names of primers used for RACE experiments are indicated to the left of sequences and indicate whether they were used for 3’ (3’R) or 5’ (5’R) RACE analysis. Primer annealing positions for RACE experiments are underlined. Sequences reported by Chen *et al*. 2007 (27) are also shown.

Box H/ACA RNAs

GlsR26 — GLCHR05:1459471-1459636

```
>ODE3 3’R #1 CAAAGAGTTTCCTTCATTCAATTTTTAGATCCATCTGGCGCACGTTACGAGGCCTGGCTCCCGGTCCGGCCCTACCTTGCGTGCGCATATCTCCGGGATCTGCGCCGCGTCTGCTCGCGGCGATTTCGGTTATGCCGGCCCGAACACTCCTTCATTCAACAGGCCC
>ODE3 3’R #2 CAAAGAGTTTCCTTCATTCAATTTTTAGATCCATCTGGCGCACGTTACGAGGCCTGGCTCCCGGTCCGGCCCTACCTTGCGTGCGCATATCTCCGGGATCTGCGCCGCGTCTGCTCGCGGCGATTTCGGTTATGCCGGCCCGAACACTCCTTCATTCAACAGGCCC
>oAH60 5’R #1CAAAGAGTTTCCTTCATTCAATTTTTAGATCCATCTGGCGCACGTTACGAGGCCTGGCTCCCGGTCCGGCCCTACCTTGCGTGCGCATATCTCCGGGATCTGCGCCGCGTCTGCTCGCGGCGATTTCGGTTATGCCGGCCCGAACACTCCTTCATTCAACAGGCCC
>oAH60 5’R #2CAAAGAGTTTCCTTCATTCAATTTTTAGATCCATCTGGCGCACGTTACGAGGCCTGGCTCCCGGTCCGGCCCTACCTTGCGTGCGCATATCTCCGGGATCTGCGCCGCGTCTGCTCGCGGCGATTTCGGTTATGCCGGCCCGAACACTCCTTCATTCAACAGGCCC
```

GlsR27 — GLCHR01:1371240-1371405

```
>ODE4 3’R #1 GGGCGAGATATCTTTCAGCATTTATAACCAAAAAATTAAGCTCACCCAAAGTCAACGGAGCGCCAGCTACGTGTTATGGGCAGCGAAAGTACCAGAGCCAAAGAGTTCCTCTGATCGCCTGGCCGGAGCACATTTGTGATCTCCTATACCTTCATTTAATTAGCGT
>ODE4 3’R #2 GGGCGAGATATCTTTCAGCATTTATAACCAAAAAATTAAGCTCACCCAAAGTCAACGGAGCGCCAGCTACGTGTTATGGGCAGCGAAAGTACCAGAGCCAAAGAGTTCCTCTGATCGCCTGGCCGGAGCACATTTGTGATCTCCTATACCTTCATTTAATTAGCGT
>OAH62 5’R #1GGGCGAGATATCTTTCAGCATTTATAACCAAAAAATTAAGCTCACCCAAAGTCAACGGAGCGCCAGCTACGTGTTATGGGCAGCGAAAGTACCAGAGCCAAAGAGTTCCTCTGATCGCCTGGCCGGAGCACATTTGTGATCTCCTATACCTTCATTTAATTAGCGT
>OAH62 5’R #2GGGCGAGATATCTTTCAGCATTTATAACCAAAAAATTAAGCTCACCCAAAGTCAACGGAGCGCCAGCTACGTGTTATGGGCAGCGAAAGTACCAGAGCCAAAGAGTTCCTCTGATCGCCTGGCCGGAGCACATTTGTGATCTCCTATACCTTCATTTAATTAGCGT
>OAH62 5’R #3GGGCGAGATATCTTTCAGCATTTATAACCAAAAAATTAAGCTCACCCAAAGTCAACGGAGCGCCAGCTACGTGTTATGGGCAGCGAAAGTACCAGAGCCAAAGAGTTCCTCTGATCGCCTGGCCGGAGCACATTTGTGATCTCCTATACCTTCATTTAATTAGCGT
>OAH62 5’R #4GGGCGAGATATCTTTCAGCATTTATAACCAAAAAATTAAGCTCACCCAAAGTCAACGGAGCGCCAGCTACGTGTTATGGGCAGCGAAAGTACCAGAGCCAAAGAGTTCCTCTGATCGCCTGGCCGGAGCACATTTGTGATCTCCTATACCTTCATTTAATTAGCGT
```

NcRNAs without Assigned Function

Candidate-5 — GLCHR03:299369-299534

```
>CHEN ET AL. GTGAAGGGAAGCAGACTCCATGGCATAAATAAATGCAAAATTCTTTAACCTGAAAACAAAATGGCTAGCAACACGAGGAAACGAGTGTTTCGCCGGGCATAACTGGGCATGCATTTTCCTTGCCCAGTCTGCCTCCATACTAATTTCTCCTTTACTCAATCAGGAT
>ODE8 3’R #1 GTGAAGGGAAGCAGACTCCATGGCATAAATAAATGCAAAATTCTTTAACCTGAAAACAAAATGGCTAGCAACACGAGGAAACGAGTGTTTCGCCGGGCATAACTGGGCATGCATTTTCCTTGCCCAGTCTGCCTCCATACTAATTTCTCCTTTACTCAATCAGGAT
>ODE8 3’R #2 GTGAAGGGAAGCAGACTCCATGGCATAAATAAATGCAAAATTCTTTAACCTGAAAACAAAATGGCTAGCAACACGAGGAAACGAGTGTTTCGCCGGGCATAACTGGGCATGCATTTTCCTTGCCCAGTCTGCCTCCATACTAATTTCTCCTTTACTCAATCAGGAT
```

Candidate-12 — GLCHR03:474659-474824

```
>CHEN ET AL. TTCTTAGTCTCTTCTTAGCATCCAGAATAAATCACATTAAATGTATTTTAATTTGAATTTTGATCCCCCGAGAAAAAGAACCCCAACCCGATGACGAATAGCTGTCCTGGCGGAGGCGGTCATGACGACGAAGCCATCACGTAGGATCCCTTCACTCAACCTCTGC
>ODE2 3’R #2 TTCTTAGTCTCTTCTTAGCATCCAGAATAAATCACATTAAATGTATTTTAATTTGAATTTTGATCCCCCGAGAAAAAGAACCCCAACCCGATGACGAATAGCTGTCCTGGCGGAGGCGGTCATGACGACGAAGCCATCACGTAGGATCCCTTCACTCAACCTCTGC
```

Candidate-14 — GLCHR04:581396-581561

```
>CHEN ET AL. AGGCAAAAATTAAAATCAGAGTCGGCTTCGACTTTAGTGTAGTTACTGTTTCGTCGGCTTAACCGCCGATCCACTACATGCAAGGGGCAGCCGGGCTGTGAGGCAGCTGCCAGGATGGTCCTGCCCTTGTCCCGGCTGGCGCCGTCCACCTTTATTCAAGTTTTCT
>ODE9 3’R #1 AGGCAAAAATTAAAATCAGAGTCGGCTTCGACTTTAGTGTAGTTACTGTTTCGTCGGCTTAACCGCCGATCCACTACATGCAAGGGGCAGCCGGGCTGTGAGGCAGCTGCCAGGATGGTCCTGCCCTTGTCCCGGCTGGCGCCGTCCACCTTTATTCAAGTTTTCT
>ODE9 3’R #2 AGGCAAAAATTAAAATCAGAGTCGGCTTCGACTTTAGTGTAGTTACTGTTTCGTCGGCTTAACCGCCGATCCACTACATGCAAGGGGCAGCCGGGCTGTGAGGCAGCTGCCAGGATGGTCCTGCCCTTGTCCCGGCTGGCGCCGTCCACCTTTATTCAAGTTTTCT
```

Candidate-15 — GLCHR02:350512-350677

```
>CHEN ET AL. CGGCCTCTGGCTTGGACCCCGTGGCGTCGCCGGCCTCCGCGGAGGCAGGGGCCGGCCCGCCTTCAACTCAGCCGGACAGCCGGAGGCCGGAGACGGAGCACGGTCAGGCGGGCGGGGTGCAGTGCCAGCCCCAGCCGCAGAGCGGCTTCCTTTACTCAAGATCGGG
>ODE5 3’R #1 CGGCCTCTGGCTTGGACCCCGTGGCGTCGCCGGCCTCCGCGGAGGCAGGGGCCGGCCCGCCTTCAACTCAGCCGGACAGCCGGAGGCCGGAGACGGAGCACGGTCAGGCGGGCGGGGTGCAGTGCCAGCCCCAGCCGCAGAGCGGCTTCCTTTACTCAAGATCGGG
>ODE5 3’R #1 CGGCCTCTGGCTTGGACCCCGTGGCGTCGCCGGCCTCCGCGGAGGCAGGGGCCGGCCCGCCTTCAACTCAGCCGGACAGCCGGAGGCCGGAGACGGAGCACGGTCAGGCGGGCGGGGTGCAGTGCCAGCCCCAGCCGCAGAGCGGCTTCCTTTACTCAAGATCGGG
```

Candidate-17 — GLCHR03:1601283-1601448

```
>CHEN ET AL. GAGTTAATACCACCAAACCCCTGTGCGTACATGTCGCCCCCTAACCTTCTGATGCGGATACCTTGCCGCAGGGCCGTTAAGCGAGGCTTGGCCCGTGCGACGATGAGGCTCCCTGCGGGGAAGCCCTGCGGCGCGTCTTAAGGAGGCTCCTTCACTCAACGGCGTC
>ODE1 3’R #1 GAGTTAATACCACCAAACCCCTGTGCGTACATGTCGCCCCCTAACCTTCTGATGCGGATACCTTGCCGCAGGGCCGTTAAGCGAGGCTTGGCCCGTGCGACGATGAGGCTCCCTGCGGGGAAGCCCTGCGGCGCGTCTTAAGGAGGCTCCTTCACTCAACGGCGTC
>ODE1 3’R #1 GAGTTAATACCACCAAACCCCTGTGCGTACATGTCGCCCCCTAACCTTCTGATGCGGATACCTTGCCGCAGGGCCGTTAAGCGAGGCTTGGCCCGTGCGACGATGAGGCTCCCTGCGGGGAAGCCCTGCGGCGCGTCTTAAGGAGGCTCCTTCACTCAACGGCGTC
```

Candidate-18 — GLCHR01:479835-480000

```
>CHEN ET AL. TCCCTGGGCGTCGGGCAGAAAGTGCCGGTCCTCTGGATTCCGGGGAGTGTCTGGTGCCGATCGGACACTCCCTAGCCGCCACACTGACAGTTATGGTTGCAGGACAAGCTTAGCGAGTCCGAACTCGACAGGGATACTCTACAGCGTTCCTTTATTCAATCATTGA
>ODE10 3’R #1TCCCTGGGCGTCGGGCAGAAAGTGCCGGTCCTCTGGATTCCGGGGAGTGTCTGGTGCCGATCGGACACTCCCTAGCCGCCACACTGACAGTTATGGTTGCAGGACAAGCTTAGCGAGTCCGAACTCGACAGGGATACTCTACAGCGTTCCTTTATTCAATCATTGA
>ODE10 3’R #1TCCCTGGGCGTCGGGCAGAAAGTGCCGGTCCTCTGGATTCCGGGGAGTGTCTGGTGCCGATCGGACACTCCCTAGCCGCCACACTGACAGTTATGGTTGCAGGACAAGCTTAGCGAGTCCGAACTCGACAGGGATACTCTACAGCGTTCCTTTATTCAATCATTGA
```

Candidate-23 — GLCHR01:449998-450163

```
>CHEN ET AL. GAGGCATGTATAATTATACCAAAATTAATTGCAGAGTTCTCCTTTTTCAAAAAGCCTCTCTGTAGGTAGGGCCGATGAGCTATTTTGTACCACTCTGACCGTGAGGCGTATGCCTAGGGCATGGAGAAGAGCAGACTTGAGGCCTGTTCCTTTACTCAATTTTGTG
>oDE7 3’R #1 GAGGCATGTATAATTATACCAAAATTAATTGCAGAGTTCTCCTTTTTCAAAAAGCCTCTCTGTAGGTAGGGCCGATGAGCTATTTTGTACCACTCTGACCGTGAGGCGTATGCCTAGGGCATGGAGAAGAGCAGACTTGAGGCCTGTTCCTTTACTCAATTTTGTG
>oDE7 3’R #2 GAGGCATGTATAATTATACCAAAATTAATTGCAGAGTTCTCCTTTTTCAAAAAGCCTCTCTGTAGGTAGGGCCGATGAGCTATTTTGTACCACTCTGACCGTGAGGCGTATGCCTAGGGCATGGAGAAGAGCAGACTTGAGGCCTGTTCCTTTACTCAATTTTGTG
```

GlsR28 — GLCHR01:978028-978193

```
>ODE12 3’R #1AAACTGCACCCTTGTGTTACTCTGGTGTGTTCTTTATTACCCTACTCTGTCTCGTGAACCCTCACCACCAGTTCTTCTCGCAGTGGTCTCTGTCAAAGACTGCCGCACGGTACACCAGAAGCAAGGGGAAGGATCCCATCCACGCAGTCCTTTACTTAAACATGGG
>ODE12 3’R #2AAACTGCACCCTTGTGTTACTCTGGTGTGTTCTTTATTACCCTACTCTGTCTCGTGAACCCTCACCACCAGTTCTTCTCGCAGTGGTCTCTGTCAAAGACTGCCGCACGGTACACCAGAAGCAAGGGGAAGGATCCCATCCACGCAGTCCTTTACTTAAACATGGG
>ODE12 5’R #1AAACTGCACCCTTGTGTTACTCTGGTGTGTTCTTTATTACCCTACTCTGTCTCGTGAACCCTCACCACCAGTTCTTCTCGCAGTGGTCTCTGTCAAAGACTGCCGCACGGTACACCAGAAGCAAGGGGAAGGATCCCATCCACGCAGTCCTTTACTTAAACATGGG
>ODE12 5’R #2AAACTGCACCCTTGTGTTACTCTGGTGTGTTCTTTATTACCCTACTCTGTCTCGTGAACCCTCACCACCAGTTCTTCTCGCAGTGGTCTCTGTCAAAGACTGCCGCACGGTACACCAGAAGCAAGGGGAAGGATCCCATCCACGCAGTCCTTTACTTAAACATGGG
```

Spliceosomal snRNA Candidates

U1 Candidate — GLCHR03:258090-258039

```
>oAH133 3'R#1TGAGCAGGTCAAAAATTGAAGGTAATTTTAACTTACCTCAAGGGTGGCGACGAGCCAGTGTTCGGGCCAGGCTGGTGCTGCGCATACCGCGCTGGCACTGGTCACGGGGCAGTGCTCTCAGACCTGCTACCGTACCCTTTTAATTTTTCCTTCACTTAAGGCCCAT
>oAH133 3'R#2TGAGCAGGTCAAAAATTGAAGGTAATTTTAACTTACCTCAAGGGTGGCGACGAGCCAGTGTTCGGGCCAGGCTGGTGCTGCGCATACCGCGCTGGCACTGGTCACGGGGCAGTGCTCTCAGACCTGCTACCGTACCCTTTTAATTTTTCCTTCACTTAAGGCCCAT
>oAH95 5'R#1 TGAGCAGGTCAAAAATTGAAGGTAATTTTAACTTACCTCAAGGGTGGCGACGAGCCAGTGTTCGGGCCAGGCTGGTGCTGCGCATACCGCGCTGGCACTGGTCACGGGGCAGTGCTCTCAGACCTGCTACCGTACCCTTTTAATTTTTCCTTCACTTAAGGCCCAT
>oAH95 5'R#2 TGAGCAGGTCAAAAATTGAAGGTAATTTTAACTTACCTCAAGGGTGGCGACGAGCCAGTGTTCGGGCCAGGCTGGTGCTGCGCATACCGCGCTGGCACTGGTCACGGGGCAGTGCTCTCAGACCTGCTACCGTACCCTTTTAATTTTTCCTTCACTTAAGGCCCAT
>oAH95 5'R#3 TGAGCAGGTCAAAAATTGAAGGTAATTTTAACTTACCTCAAGGGTGGCGACGAGCCAGTGTTCGGGCCAGGCTGGTGCTGCGCATACCGCGCTGGCACTGGTCACGGGGCAGTGCTCTCAGACCTGCTACCGTACCCTTTTAATTTTTCCTTCACTTAAGGCCCAT
>oAH95 5'R#4 TGAGCAGGTCAAAAATTGAAGGTAATTTTAACTTACCTCAAGGGTGGCGACGAGCCAGTGTTCGGGCCAGGCTGGTGCTGCGCATACCGCGCTGGCACTGGTCACGGGGCAGTGCTCTCAGACCTGCTACCGTACCCTTTTAATTTTTCCTTCACTTAAGGCCCAT
```

Candidate-14 (U2 Candidate) — GLCHR04:581396-581561

```
>CHEN ET AL. AGGCAAAAATTAAAATCAGAGTCGGCTTCGACTTTAGTGTAGTTACTGTTTCGTCGGCTTAACCGCCGATCCACTACATGCAAGGGGCAGCCGGGCTGTGAGGCAGCTGCCAGGATGGTCCTGCCCTTGTCCCGGCTGGCGCCGTCCACCTTTATTCAAGTTTTCT
>ODE9 3'R #1 AGGCAAAAATTAAAATCAGAGTCGGCTTCGACTTTAGTGTAGTTACTGTTTCGTCGGCTTAACCGCCGATCCACTACATGCAAGGGGCAGCCGGGCTGTGAGGCAGCTGCCAGGATGGTCCTGCCCTTGTCCCGGCTGGCGCCGTCCACCTTTATTCAAGTTTTCT
>ODE9 3'R #2 AGGCAAAAATTAAAATCAGAGTCGGCTTCGACTTTAGTGTAGTTACTGTTTCGTCGGCTTAACCGCCGATCCACTACATGCAAGGGGCAGCCGGGCTGTGAGGCAGCTGCCAGGATGGTCCTGCCCTTGTCCCGGCTGGCGCCGTCCACCTTTATTCAAGTTTTCT
>OAH120 3'R#1AGGCAAAAATTAAAATCAGAGTCGGCTTCGACTTTAGTGTAGTTACTGTTTCGTCGGCTTAACCGCCGATCCACTACATGCAAGGGGCAGCCGGGCTGTGAGGCAGCTGCCAGGATGGTCCTGCCCTTGTCCCGGCTGGCGCCGTCCACCTTTATTCAAGTTTTCT
>OAH120 3'R#2AGGCAAAAATTAAAATCAGAGTCGGCTTCGACTTTAGTGTAGTTACTGTTTCGTCGGCTTAACCGCCGATCCACTACATGCAAGGGGCAGCCGGGCTGTGAGGCAGCTGCCAGGATGGTCCTGCCCTTGTCCCGGCTGGCGCCGTCCACCTTTATTCAAGTTTTCT
>OAH140 5'R#1AGGCAAAAATTAAAATCAGAGTCGGCTTCGACTTTAGTGTAGTTACTGTTTCGTCGGCTTAACCGCCGATCCACTACATGCAAGGGGCAGCCGGGCTGTGAGGCAGCTGCCAGGATGGTCCTGCCCTTGTCCCGGCTGGCGCCGTCCACCTTTATTCAAGTTTTCT
```

Candidate-11 (U4 Candidate) — GLCHR02:1194980-1195145

```
>Chen et al. TGTAAAATAAACTATTTTAAATTCAATTCTGTAAAATAAATTTTTTATTTTTTGACTCTAGGCTGAAGCTGCCAAGGTGCGTGATCCCTCGGTGATGCCTTGAGTGTTGCTTCACCAAAGAACAACCACACGGCACAGCCGAATCTCTCATTTTTTAAACTTTCTC
>oAH118 3'R#1TGTAAAATAAACTATTTTAAATTCAATTCTGTAAAATAAATTTTTTATTTTTTGACTCTAGGCTGAAGCTGCCAAGGTGCGTGATCCCTCGGTGATGCCTTGAGTGTTGCTTCACCAAAGAACAACCACACGGCACAGCCGAATCTCTCATTTTTTAAACTTTCTC
>oAH118 3'R#2TGTAAAATAAACTATTTTAAATTCAATTCTGTAAAATAAATTTTTTATTTTTTGACTCTAGGCTGAAGCTGCCAAGGTGCGTGATCCCTCGGTGATGCCTTGAGTGTTGCTTCACCAAAGAACAACCACACGGCACAGCCGAATCTCTCATTTTTTAAACTTTCTC
>oAH139 5'R#1TGTAAAATAAACTATTTTAAATTCAATTCTGTAAAATAAATTTTTTATTTTTTGACTCTAGGCTGAAGCTGCCAAGGTGCGTGATCCCTCGGTGATGCCTTGAGTGTTGCTTCACCAAAGAACAACCACACGGCACAGCCGAATCTCTCATTTTTTAAACTTTCTC
```

U6 Candidate — GLCHR04:1813414-1813579

```
>oDE14 3'R#1 TAAACCATTTTAAATTGAAATAGGCGGTTGGAAATAAAAGCGCGGCGTGGTTAACAAAAACAGAGACAGTTAGCACCAGCTTCAGTCTAGAGTCGCTGGGGGACCTCTGGTTTCGCGGGAGCCCGTTGGCGCGTGCTTGCACCCCGCTCCTTTTCTCAATCTTCGC
>oDE14 3'R#2 TAAACCATTTTAAATTGAAATAGGCGGTTGGAAATAAAAGCGCGGCGTGGTTAACAAAAACAGAGACAGTTAGCACCAGCTTCAGTCTAGAGTCGCTGGGGGACCTCTGGTTTCGCGGGAGCCCGTTGGCGCGTGCTTGCACCCCGCTCCTTTTCTCAATCTTCGC
>oAH72 5'R#1 TAAACCATTTTAAATTGAAATAGGCGGTTGGAAATAAAAGCGCGGCGTGGTTAACAAAAACAGAGACAGTTAGCACCAGCTTCAGTCTAGAGTCGCTGGGGGACCTCTGGTTTCGCGGGAGCCCGTTGGCGCGTGCTTGCACCCCGCTCCTTTTCTCAATCTTCGC
>oAH72 5'R#2 TAAACCATTTTAAATTGAAATAGGCGGTTGGAAATAAAAGCGCGGCGTGGTTAACAAAAACAGAGACAGTTAGCACCAGCTTCAGTCTAGAGTCGCTGGGGGACCTCTGGTTTCGCGGGAGCCCGTTGGCGCGTGCTTGCACCCCGCTCCTTTTCTCAATCTTCGC
```

Trans-Spliced Introns

HSP90_EX_1 — GLCHR05:2515245-2515410

```
>OAH102 3'R#1TCGACGGCGCCGCCCAGTTCCGCGGGATCCTCTTCATCCCCAAGCGCGCGCCCTTCGACATGTGGGACGCTCAGAAGAAGAAGACGGGCATCAAGCTCATGGTCAAGAAAGTGTATGTTATGTTTGTATGCTGTATGTGTGCGAGACTCCTTTACTCAAATTTGCG
>OAH102 3'R#2TCGACGGCGCCGCCCAGTTCCGCGGGATCCTCTTCATCCCCAAGCGCGCGCCCTTCGACATGTGGGACGCTCAGAAGAAGAAGACGGGCATCAAGCTCATGGTCAAGAAAGTGTATGTTATGTTTGTATGCTGTATGTGTGCGAGACTCCTTTACTCAAATTTGCG
```

DHC_β_EX_2 — GLCHR03:577578-577743

```
>OAH103 3'R#1GCCCGTAAGAAATACAAAGCTGTCATGGACAACGTTGCAAAGCTGAACAAAAAGCTCCAAACTCTCAAGGATCAATTTGACAAGGGTATGTTACTGGGTGAAACGCTACTTATGTATGTATGCTTATATGTCTTCGCGCTCAGGCGCTCCTTTACTCAATTATCAG
>OAH103 3'R#2GCCCGTAAGAAATACAAAGCTGTCATGGACAACGTTGCAAAGCTGAACAAAAAGCTCCAAACTCTCAAGGATCAATTTGACAAGGGTATGTTACTGGGTGAAACGCTACTTATGTATGTATGCTTATATGTCTTCGCGCTCAGGCGCTCCTTTACTCAATTATCAG
```

DHC_β_EX_3 — GLCHR05:4266308-4266473

```
>OAH104 3'R#1GTTTGCTCTGATCTTCTTCCATGCAATCGTCATCGAGAGACGGAAGTTCGGTCCTATAGGGTATGTTTGTAATCTGTGTAGTCGCAGTATGCCATTATTTTATAACGTGTATGTCATTATGTCAGTATGCCAGTGCGCTGGTGAGTTTCCTTTATTCAAATGTTGT
>OAH104 3'R#2GTTTGCTCTGATCTTCTTCCATGCAATCGTCATCGAGAGACGGAAGTTCGGTCCTATAGGGTATGTTTGTAATCTGTGTAGTCGCAGTATGCCATTATTTTATAACGTGTATGTCATTATGTCAGTATGCCAGTGCGCTGGTGAGTTTCCTTTATTCAAATGTTGT
```

DHC_γ_EX_1 — GLCHR03:967465-967630

```
>OAH105 3'R#1TCTTGCGGTATTTTCATCACGATGAACCCCGGTTACGCCGGGCGTCAAGAACTTCCAGAGAATCTCAAAGCCTTATTCCGTAGCGTTGCAATGATATGTTCACAGGTGGTTTGGTGTGTATGCTTGGCGTGTATGTGTGTATGTTCCTCCTTTACTCAATACTTGG
>OAH105 3'R#2TCTTGCGGTATTTTCATCACGATGAACCCCGGTTACGCCGGGCGTCAAGAACTTCCAGAGAATCTCAAAGCCTTATTCCGTAGCGTTGCAATGATATGTTCACAGGTGGTTTGGTGTGTATGCTTGGCGTGTATGTGTGTATGTTCCTCCTTTACTCAATACTTGG
```

Supplementary Figure 6. Motif inclusion (no cleavage) in mature mRNAs

(A) RT-PCR analysis of expression of *G. lamblia* protein coding sequences containing internal motif sequences. Experiments were carried out in either the presence (RT +) or absence (RT -) of SuperScript II™ reverse transcriptase and PCR products were resolved by 2% agarose gel electrophoresis. Expected sizes for products of Rhp26p mRNA (lanes 1 and 2), U5 snRNP 200 kDa helicase mRNA (3 and 4), protein 21.1 mRNA (5 and 6) and hypothetical protein (7 and 8) are indicated in parentheses and size marker (M) bands are in base-pairs. (B) Sequencing results for RT-PCR experiments (top sequence) are compared to *G. lamblia* WB isolate genomic sequences (bottom sequence). Open reading frame (ORF) identifiers are provided with the specific regions amplified indicated. Motif sequences within ORFs are highlighted in green and primer binding sites are shown in bold text with arrows indicating direction of amplification during PCR.

A

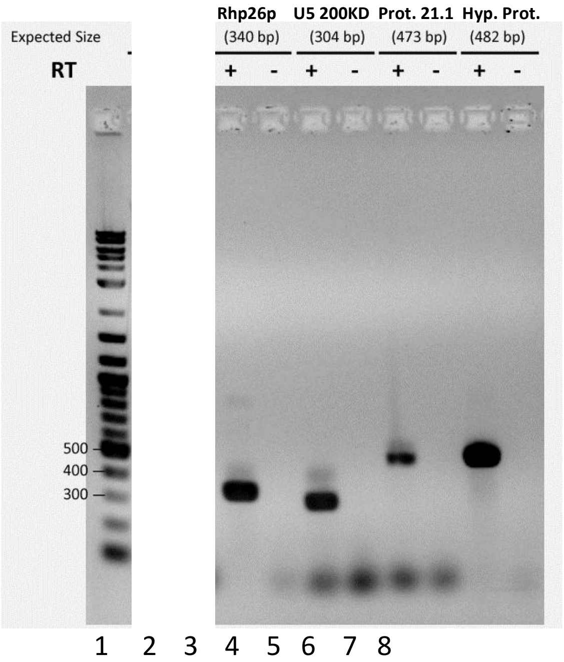

B

1. DNA repair and recombination protein Rhp26p (GL50803_87205:1009-1348)

```
                 oDE17
RT-PCR     1 CTATCCAGCCAAGAGCCGTGTATTCAGTATGCTATTTTCGATGAAGTTCACTATTTGAAAAATACAGAAACAATTCGCATCAAGGCATTGCGGGCCCTTAGGATATCATGTAAGCTTGGT 120
             ||||||||||||||||||||||||||||||||||||||||||||||||||||||||||||||||||||||||||||||||||||||||||||||||||||||||||||||||||||||||
Gl_WB   1009 CTATCCAGCCAAGAGCCGTGTATTCAGTATGCTATTTTCGATGAAGTTCACTATTTGAAAAATACAGAAACAATTCGCATCAAGGCATTGCGGGCCCTTAGGATATCATGTAAGCTTGGT 1128

RT-PCR   121 ATCTCAGCAACGCCCGTTCAAAATAGTTTAGTAGAGATCTATTCACTGATAACCTTCATTCAACCAAACATTCTTGGAGATTATGATTCCTTTCTGCAAGAGTATGACATTCCCATTCGC 240
             ||||||||||||||||||||||||||||||||||||||||||||||||||||||||||||||||||||||||||||||||||||||||||||||||||||||||||||||||||||||||
Gl_WB   1129 ATCTCAGCAACGCCCGTTCAAAATAGTTTAGTAGAGATCTATTCACTGATAACCTTCATTCAACCAAACATTCTTGGAGATTATGATTCCTTTCTGCAAGAGTATGACATTCCCATTCGC 1248

RT-PCR   241 AAGGGATCAATGGAAAATTCTAATTATGAGGACATATCTTTGGCGGCCTCCTTGGCACAGAGACTTGCAAATAGACTCAAACCTTACATACTACGACGCC 340
             ||||||||||||||||||||||||||||||||||||||||||||||||||||||||||||||||||||||||||||||||||||||||||||||||||||
Gl_WB   1249 AAGGGATCAATGGAAAATTCTAATTATGAGGACATATCTTTGGCGGCCTCCTTGGCACAGAGACTTGCAAATAGACTCAAACCTTACATACTACGACGCC 1348
                                                                                            oDE18
```

2. U5 small nuclear ribonucleoprotein 200 kDa helicase, putative (GL50803_9352: 2570-2873)

```
              oDE19
RT-PCR     1 GATGCCTTAGAGAACTTGCGTGCCTTCTCATCCAAAGCAAATCTCACCAGGCTCTAACCAGGCTGTATTGTATGCTTCAGTCTCGATGCTGGCCTGCGTTTCTATTTGTGCGAGAAGTTC 120
             ||||||||||||||||||||||||||||||||||||||||||||||||||||||||||||||||||||||||||||||||||||||||||||||||||||||||||||||||||||||||
Gl_WB   2570 GATGCCTTAGAGAACTTGCGTGCCTTCTCATCCAAAGCAAATCTCACCAGGCTCTAACCAGGCTGTATTGTATGCTTCAGTCTCGATGCTGGCCTGCGTTTCTATTTGTGCGAGAAGTTC 2689

RT-PCR   121 TGAGCAATCCTTTATTCAAATTCGATTTTCTCTCACCAGAGTACATACTGTGGATAGAACAATTAGAGTTAAAACAATTCAGTCCAAATGTTCTTCGCAGATTGACAGTAGACCAGCTTT 240
             ||||||||||||||||||||||||||||||||||||||||||||||||||||||||||||||||||||||||||||||||||||||||||||||||||||||||||||||||||||||||
Gl_WB   2690 TGAGCAATCCTTTATTCAAATTCGATTTTCTCTCACCAGAGTACATACTGTGGATAGAACAATTAGAGTTAAAACAATTCAGTCCAAATGTTCTTCGCAGATTGACAGTAGACCAGCTTT 2809

RT-PCR   241 CTCAGATAATTGTTTCTCCAGATGATAGAACCCCACTCGATAAACGGCAGCTATCTCATCTACG 304
             ||||||||||||||||||||||||||||||||||||||||||||||||||||||||||||||||
Gl_WB   2810 CTCAGATAATTGTTTCTCCAGATGATAGAACCCCACTCGATAAACGGCAGCTATCTCATCTACG 2873
                                                               oDE18
```

3. Protein 21.1 (GL50803_25296:542-1014)

```
              oDE21
RT-PCR     1 ATGGGTCTGACACGTTGGACAGAGACAATGCACTAGGTAGTGTTCCTACCGAGAAAAGAGCTATCAAGTCAGCTATGCGTGCAAGGACTCCGGTAAACATGATACGATTTTCCACAGAGA 120
             ||||||||||||||||||||||||||||||||||||||||||||||||||||||||||||||||||||||||||||||||||||||||||||||||||||||||||||||||||||||||
Gl_WB    542 ATGGGTCTGACACGTTGGACAGAGACAATGCACTAGGTAGTGTTCCTACCGAGAAAAGAGCTATCAAGTCAGCTATGCGTGCAAGGACTCCGGTAAACATGATACGATTTTCCACAGAGA 661

RT-PCR   121 TCTCCTTGTCCGTTCGGCGGGCGACGTCGAGACCAGCCAAATCATCTCTAGAAGCATCGGCAAACGCCTCCCTCTGTATACCAACACTCAAGTATGTTCTTGCGTTAATGAACTCCCCTT 240
             ||||||||||||||||||||||||||||||||||||||||||||||||||||||||||||||||||||||||||||||||||||||||||||||||||||||||||||||||||||||||
Gl_WB    662 TCTCCTTGTCCGTTCGGCGGGCGACGTCGAGACCAGCCAAATCATCTCTAGAAGCATCGGCAAACGCCTCCCTCTGTATACCAACACTCAAGTATGTTCTTGCGTTAATGAACTCCCCTT 781

RT-PCR   241 CATTCAATGTCATTATGACGGCAGAAGCACTGCTGAATACCCCGAATATCCACAAGCTCATGGAGCCCATCAAACAAAGCTACACACAAAAACTAGACACATCGAAGTACTTCCTTTGCC 360
             ||||||||||||||||||||||||||||||||||||||||||||||||||||||||||||||||||||||||||||||||||||||||||||||||||||||||||||||||||||||||
Gl_WB    782 CATTCAATGTCATTATGACGGCAGAAGCACTGCTGAATACCCCGAATATCCACAAGCTCATGGAGCCCATCAAACAAAGCTACACACAAAAACTAGACACATCGAAGTACTTCCTTTGCC 901

RT-PCR   361 CACTTATTGAATCTATGCTGGCAGGAGATAGCTGGTTCAGCGAGAACACGCTCTATCTTCTACACTTTGCTGGCCGTGTAGATAGAGTGGGACGCACATCGCTGATTCATCTA 473
             |||||||||||||||||||||||||||||||||||||||||||||||||||||||||||||||||||||||||||||||||||||||||||||||||||||||||||||||||
Gl_WB    902 CACTTATTGAATCTATGCTGGCAGGAGATAGCTGGTTCAGCGAGAACACGCTCTATCTTCTACACTTTGCTGGCCGTGTAGATAGAGTGGGACGCACATCGCTGATTCATCTA 1014
                                                                                                                   oDE22
```

4. Hypothetical Protein (GL50803_7350:3160-3641)

```
              oDE23
RT-PCR     1 GTGACCGTCCTCTGTACGGCAGGCGACCTCATATCGCCCTTTGTTCGACCGAACCATCTCATTAAATCGAAGTTTTCACTTAACGAGCGCACGCTGATTAGTTTGCCTTCTCCTATAAAG 120
             ||||||||||||||||||||||||||||||||||||||||||||||||||||||||||||||||||||||||||||||||||||||||||||||||||||||||||||||||||||||||
Gl_WB   3160 GTGACCGTCCTCTGTACGGCAGGCGACCTCATATCGCCCTTTGTTCGACCGAACCATCTCATTAAATCGAAGTTTTCACTTAACGAGCGCACGCTGATTAGTTTGCCTTCTCCTATAAAG 3279

RT-PCR   121 ATAAGTTTTACGTACATCAATATGTTGTCTGCCCTGGATATGGAGGAGATGTCTATGAGGTCAGAGAAGCTTTCTAAAACCACTCCCATCATAGAAAAGCTTCTGCCCTTTTCTCAAAGC 240
             ||||||||||||||||||||||||||||||||||||||||||||||||||||||||||||||||||||||||||||||||||||||||||||||||||||||||||||||||||||||||
Gl_WB   3280 ATAAGTTTTACGTACATCAATATGTTGTCTGCCCTGGATATGGAGGAGATGTCTATGAGGTCAGAGAAGCTTTCTAAAACCACTCCCATCATAGAAAAGCTTCTGCCCTTTTCTCAAAGC 3399

RT-PCR   241 TCGTACATGGCCCCACTGGTCAAATTCACTCCTTATCACAAAGGAACCACTGTCCTGTTTAAGAAGGAGGACGGGTTTGATTGGAACGAGAAGCTTCCGTCTGATCTGGCATTTGAAAGC 360
             ||||||||||||||||||||||||||||||||||||||||||||||||||||||||||||||||||||||||||||||||||||||||||||||||||||||||||||||||||||||||
Gl_WB   3400 TCGTACATGGCCCCACTGGTCAAATTCACTCCTTATCACAAAGGAACCACTGTCCTGTTTAAGAAGGAGGACGGGTTTGATTGGAACGAGAAGCTTCCGTCTGATCTGGCATTTGAAAGC 3519

RT-PCR   361 CTCGTGATCAGGGCTGTCGGGGGTTTTTCGCGCAAGTCGTACCAGGTGGACGTCAAGTCGCGGGCACGGATTGAAAAGCTAGCTACCGCAGCCTTTAAGACACGTTTTGAGCCCTTCATCTT 482
             ||||||||||||||||||||||||||||||||||||||||||||||||||||||||||||||||||||||||||||||||||||||||||||||||||||||||||||||||||||||||||
Gl_WB   3520 CTCGTGATCAGGGCTGTCGGGGGTTTTTCGCGCAAGTCGTACCAGGTGGACGTCAAGTCGCGGGCACGGATTGAAAAGCTAGCTACCGCAGCCTTTAAGACACGTTTTGAGCCCTTCATCTT 3641
                                                                                                                         oDE24
```

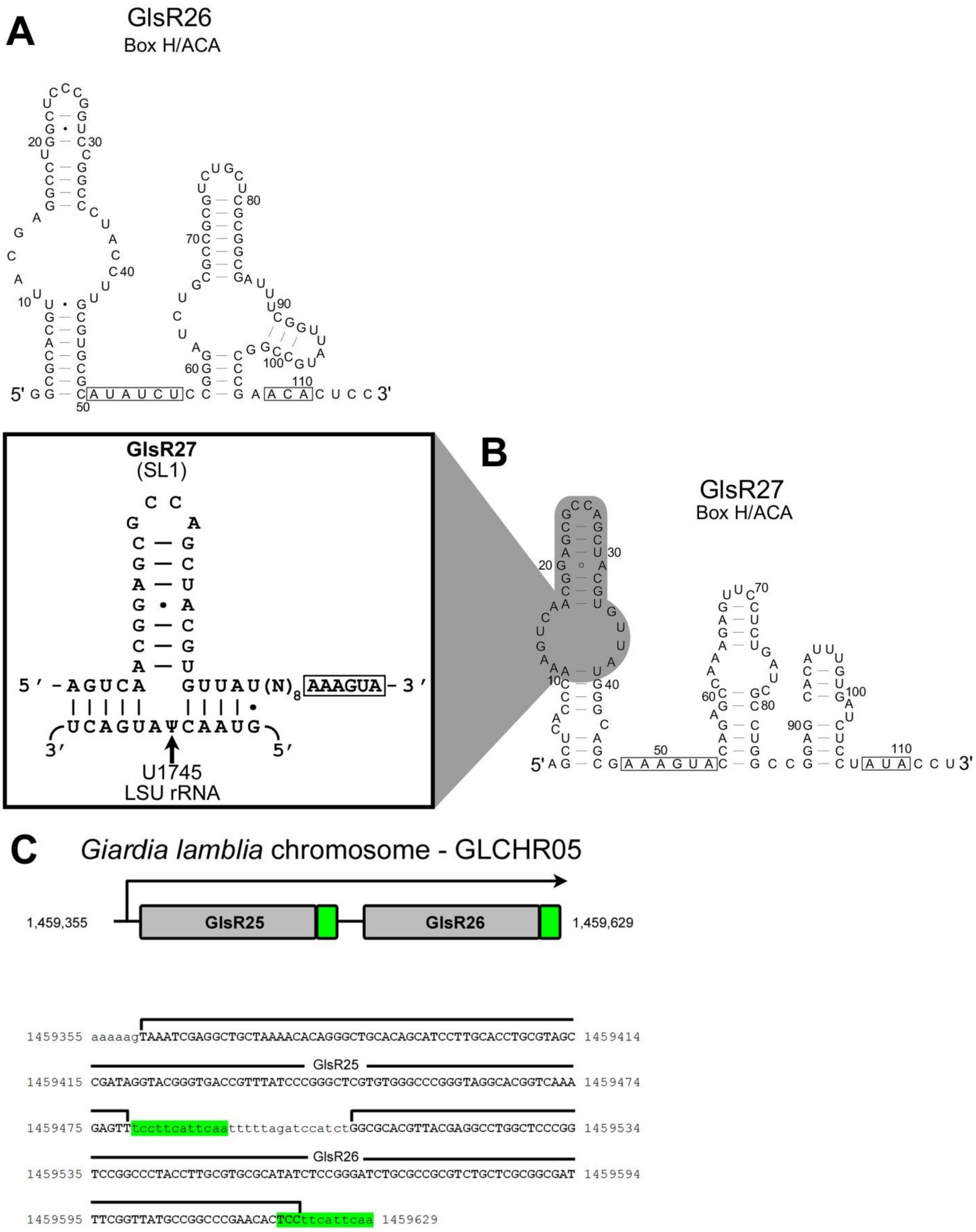

Supplementary Figure 7 – Two novel *G. lamblia* box H/ACA snoRNAs

(A and B) Secondary structures based on M-fold (31) structural predictions for GlsR26 and GlsR27 snoRNAs. Mature ends were determined by RACE experiments and the predicted “H” and “ACA” box sequence elements are in white boxes. (B, inset) The “pseudouridine pocket” formed by the predicted pairing of the guide region of GlsR27 stem-loop 1 (SL1) to the *G. lamblia* 28S large ribosomal subunit (LSU) rRNA. This interaction is predicted to target U1745 for pseudouridine formation. (C) Organization of the GlsR25-GlsR26 snoRNA gene cluster is schematically represented with genomic DNA sequence from *G. lamblia* WB isolate displayed below. A line with arrowhead indicates a predicted single transcription initiation site to produce a precursor polycistronic transcript containing both GlsR25 and GlsR26 snoRNAs. Nucleotide sequences found in the mature snoRNAs are in uppercase bold and intervening sequences in lowercase. The locations of processing motif sequences are highlighted in green.

Supplementary Figure 8 – Primary and secondary structural features of previously predicted *Giardia lamblia* U1, U2, U4 and U6 snRNA candidates

(A to D) *G. lamblia* (*G. intestinalis)* WB isolate U1, U2, U4 and U6 snRNA candidate primary nucleotide sequences identified by (24) are aligned with syntenic genomic regions of the P15 and GS isolates using ClustalW 2.0 (29). SnRNA candidate nucleotides predicted to constitute functionally critical regions are highlighted in grey with nucleotide changes observed in P15 and GS isolates indicated with red text. Where appropriate, consensus sequences for evolutionarily-conserved snRNA elements are shown above the alignments to highlight unexpected nucleotide substitutions. "5'SS" = Intron 5' splice site interacting sequence.

(E to G) Mfold based secondary structural predictions for *G. lamblia* WB isolate snRNA candidate sequences (24) are shown with nucleotide changes observed in the P15 (black arrows) and GS (grey arrows) isolates indicated.

A) U1 snRNA Candidate [identified in reference (24)]

Genome	Sequence	Start	End	Strand	#Nucleotides
G. lamblia WB	GLCHR02	846330	846451	+	122
G. lamblia GS	ACGJ01002714	394	515	+	122
G. lamblia P15	contig48	66075	66196	+	122

```
                              U1-70K Binding Site
                 5'SS           (GATCACGAAGG)
G. lamblia WB   AAACATCAGCGGCATCGTCATCACGAAGATGAGCAAAAGCATAAAGTTCGAGATCCTCAT 60
G. lamblia P15  AAACATTAGCGGCATCGTCATCATGAAGATGAGCAAAAGCATGAAGTTCGAGATCCTCAT 60
G. lamblia GS   GAACATCAAGGGCAGTGTCATCATAAACACGAATAGAAGCATGAAATTTGAAATTCTCAG 60
                 ***** *  ****  *******  ** * **  * ****** ** ** ** ** ****

                                                          Sm site
G. lamblia WB   CGTGTCTGCGAAGAGGAGGTTGACCAGGTTGCCGGCGGCAGAATTTTGGCGGGTGATGTC 120
G. lamblia P15  TGTGTCTGCGAAGAGGAGGTTGACCAGGTTACCGGCAGCAGAATTTTGGCGGGTGATGTC 120
G. lamblia GS   TGTGTCTGCAAAGAGGAGATTGACTAGGTTGCCGGCAGCAGAGTTCTGACGAGTGATATC 120
                 ******** ******** ***** ***** ***** ***** ** ** ** ***** **

G. lamblia WB   CG 122
G. lamblia P15  CG 122
G. lamblia GS   CG 122
                **
```

Maps to antisense strand of gene encoding multidrug resistance-associated protein 1 (Gene ID: GL50803_115052)

B) U2 snRNA Candidate (24)

Genome	Sequence	Start	End	Strand	#Nucleotides
G. lamblia WB	GLCHR05	2371858	2372031	+	174
G. lamblia GS	ACGJ01000614	18412	18563	-	152
G. lamblia P15	contig59	52096	52253	-	158

```
                                  U2/U6        Branch point
                                 Helix Ib     binding sequence

G. lamblia WB     ACTTGCGTCGAACCACAGCTGCATTGAACAATAGTTTCTGCTCAAATGAGAGATCAGTAT 60
G. lamblia P15    ACTTACGTCGAATCACGGTTGCATTGAACAATAGTTTTTGCTCAAACGAAAGATTAGTAT 60
G. lamblia GS     ACTTGCGTCGGATAACGGCTGCATTGAATAGCAGCTTCTGTTCAAATGAAAGATTGGTGT 60
                  **** ***** *  ** * ********* *  ** ** ** ***** ** ****  ** *

                                                       Sm site         SL III
G. lamblia WB     AATATGGCTGATTAGCGTGCAGCTGCATGCCTTTCATATTCGTTTGTTTGTTTGCTTGTT 120
G. lamblia P15    AATATGGCTGACTGGCGTGCAGCTGCATGCCCTTCGTATTCGTTTGTTT----------- 109
G. lamblia GS     AGTAGGGCTGATTAGCGTGCAGATGCATGCCCTTCATATTTGTT-GTTT----------- 108
                  * ** ****** * ******** ******** *** **** *** ****

G. lamblia WB     TGTTTTAAACTAACAACTAGGATAGTCGCCTTGCAGCGA-CAAGAATATCCTACG---- 174
G. lamblia P15    -----TAAATTAACAGCTAGGATAGTCGCCTTACAGCGA-CAAGAATATCCTACG---- 158
G. lamblia GS     -----TAAAC-AACACTGAGCACCATCATCTTACAGCAAGCAAGAGCATCAAAAGAAGT 161
                       ****  ****   ** *   **  *** **** * *****  ***  * *
```

```
                    Intron Sequence
                    Branch Point (BP)
                  3’...ACCCAGUCAA...5’
                            .||||
G. lamblia WB     5’...AACAAUAGUU...3’

G. lamblia P15    5’...AACAAUAGUU...3’
G. lamblia GS     5’...AAUAGCAGCU...3’
                     U2 Candidate
             Branch Point binding sequence
```

Overlaps Rrmp3 helicase protein coding sequence on opposite strand (Gene ID: GL50803_16747)

C) U4 snRNA Candidate (24)

Genome	Sequence	Start	End	Strand	#Nucleotides
G. lamblia WB	GLCHR05	1169325	1169457	-	133
G. lamblia GS	ACGJ01000362	2873	3005	-	133
G. lamblia P15	contig380	17649	17781	+	133

```
                   U4/U6
                  helix II                   5' stem-loop region
G. lamblia WB     AATATTGCGAGAAAACCCTCTTAGAATTGATAGAAGACAGTCCTGGCGGGATTCCAATAG 60
G. lamblia P15    AATATTGTGAGAAAACTCTCTTAGAATTGATAGAAGACAGTCCTGGCGGAATTCCAATAG 60
G. lamblia GS     AATATTGTGAGAAAACTCTCTTAGAATTGATAGAAGACAGCCCTGGGGGAATTCCAATTG 60
                  ******* ******** ********************** ***** ** ******** *

                              U4/U6
                             helix I                         Sm site
G. lamblia WB     AAACTGTTAAGCTTCTAACCTTTCAGATGCTTCGTGGTGTCGAATTTTTGTGGGAGTTCA 120
G. lamblia P15    AAACCGTTAAGCTTCTAACCTTTCAAATGCTTCGCGGTGTCGAATTTTTGTGGGAGTTCA 120
G. lamblia GS     AAACTGTTAAGCTTTTAACCTTTCAAATGCTTCGTGGTGTCGAATTTTTGTGGGAATTCA 120
                  **** ********* ********** ******** ******************** ****

G. lamblia WB     TGGAGATATGTCA 133
G. lamblia P15    TGGAGATATGTCA 133
G. lamblia GS     TGGAGATATGCCA 133
                  ********** **
```

Genomic organization: Maps to intergenic region

D) U6 snRNA Candidate (24)

Genome	Sequence	Start	End	Strand	#Nucleotides
G. lamblia WB	GLCHR05	3863206	3863322	-	117
G. lamblia GS	ACGJ01002923	23027	23143	+	117
G. lamblia P15	contig393	54509	54625	-	117

```
                                                              'ACAGAGA'     'AGC'
                                                               Element   trinucleotide
G. lamblia WB     GAAGTGTCCGGGAACAAGTGAGGCCTGCACTTTTCTGCAAACAGAGGAAGTTCAAGCTGT 60
G. lamblia P15    GAGGTGTCTGGGGATAAGTGTGGTCTACACTTTTCTGCAAATAAAGGAAGTTCGAGTTGC 60
G. lamblia GS     GAGGTGCTTGGTAACAAATGTGGCCTGCACTTCTCCGCGAACAAGGGGAGTTCAATTTGC 60
                  ** ***    **   * ** ** ** ** ***** ** ** ** *   ** ***** *   **

                  __________ISL______________
G. lamblia WB     TCGTGCATTGAGTATATTACTACAGAGTCGTGGTACTCAGACCCTACAGTGTCCTCT 117
G. lamblia P15    TCATGCATAGAGTATATCACCACTGAGTCATGGTATTCGGAGCCCGTAGTATCCTCC 117
G. lamblia GS     TCGTGCATGGAATATATTACAACAGAATCGTGGTACTCGCGTCCCGTGGTATCCTCT 117
                  ** ***** ** ***** ** ** ** ** ***** **     **     ** *****
```

Maps to antisense region of Hypothetical Protein coding sequence (GL50803_21048)

E)

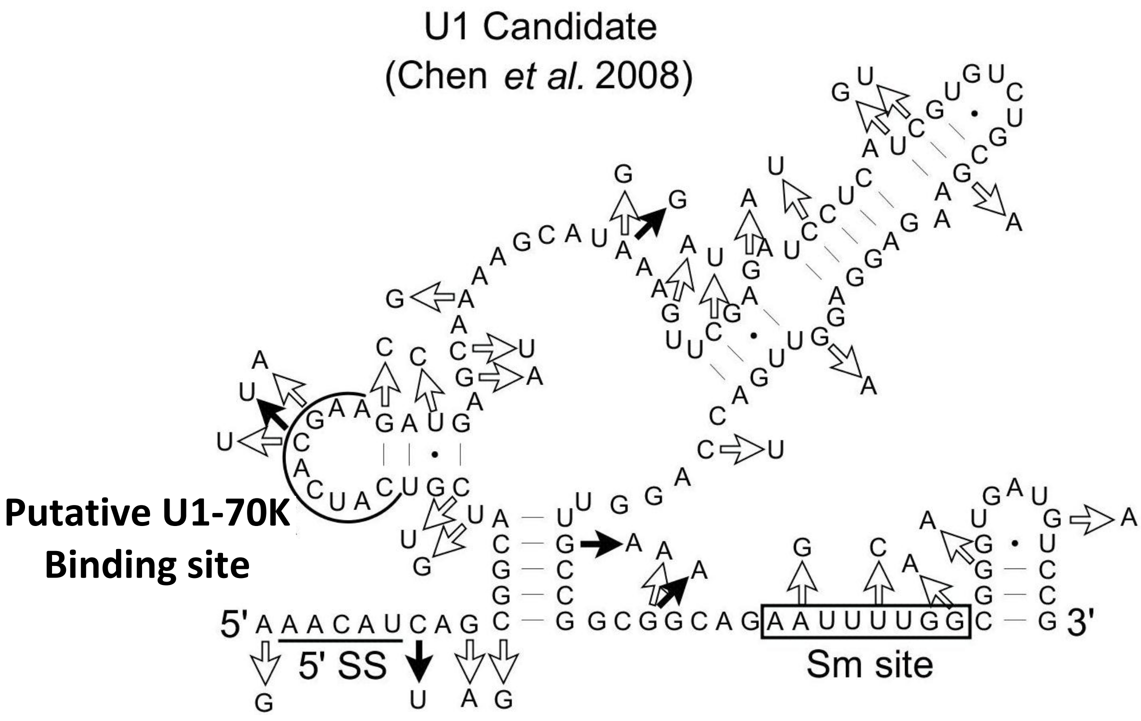
U1 Candidate
(Chen *et al.* 2008)
Putative U1-70K Binding site
5'
3'
5' SS
Sm site

F)

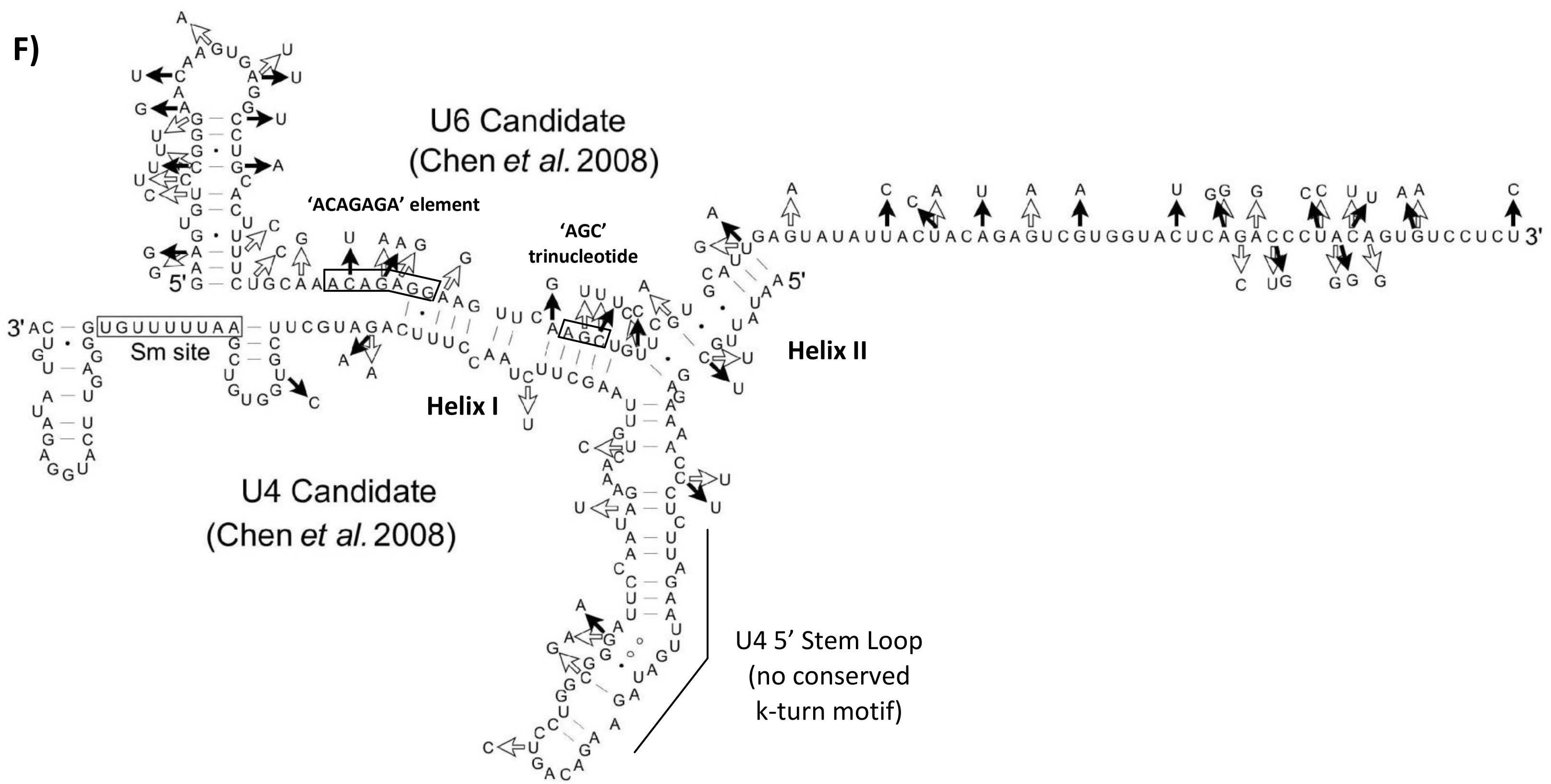
U6 Candidate
(Chen et al. 2008)
'ACAGAGA' element
'AGC' trinucleotide
Helix I
Helix II
Sm site
U4 Candidate
(Chen et al. 2008)
U4 5' Stem Loop
(no conserved
k-turn motif)

G)

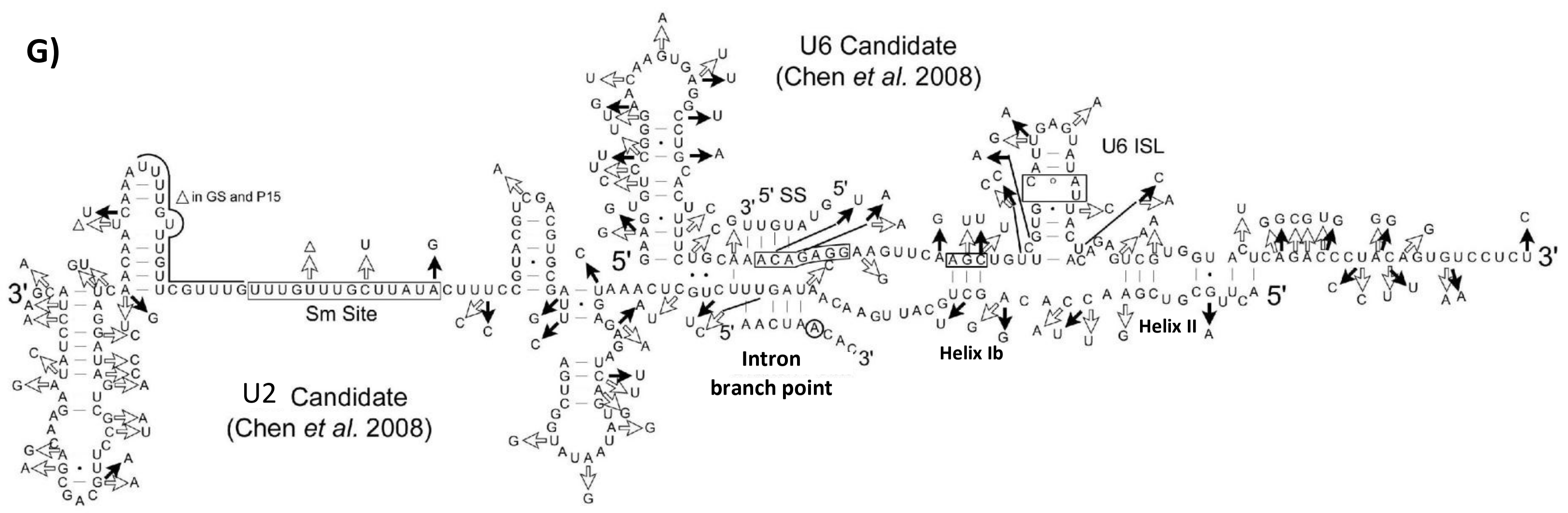
U6 Candidate
(Chen et al. 2008)
U2 Candidate
(Chen et al. 2008)
Sm Site
△ in GS and P15
5' SS
U6 ISL
Intron
branch point
Helix Ib
Helix II

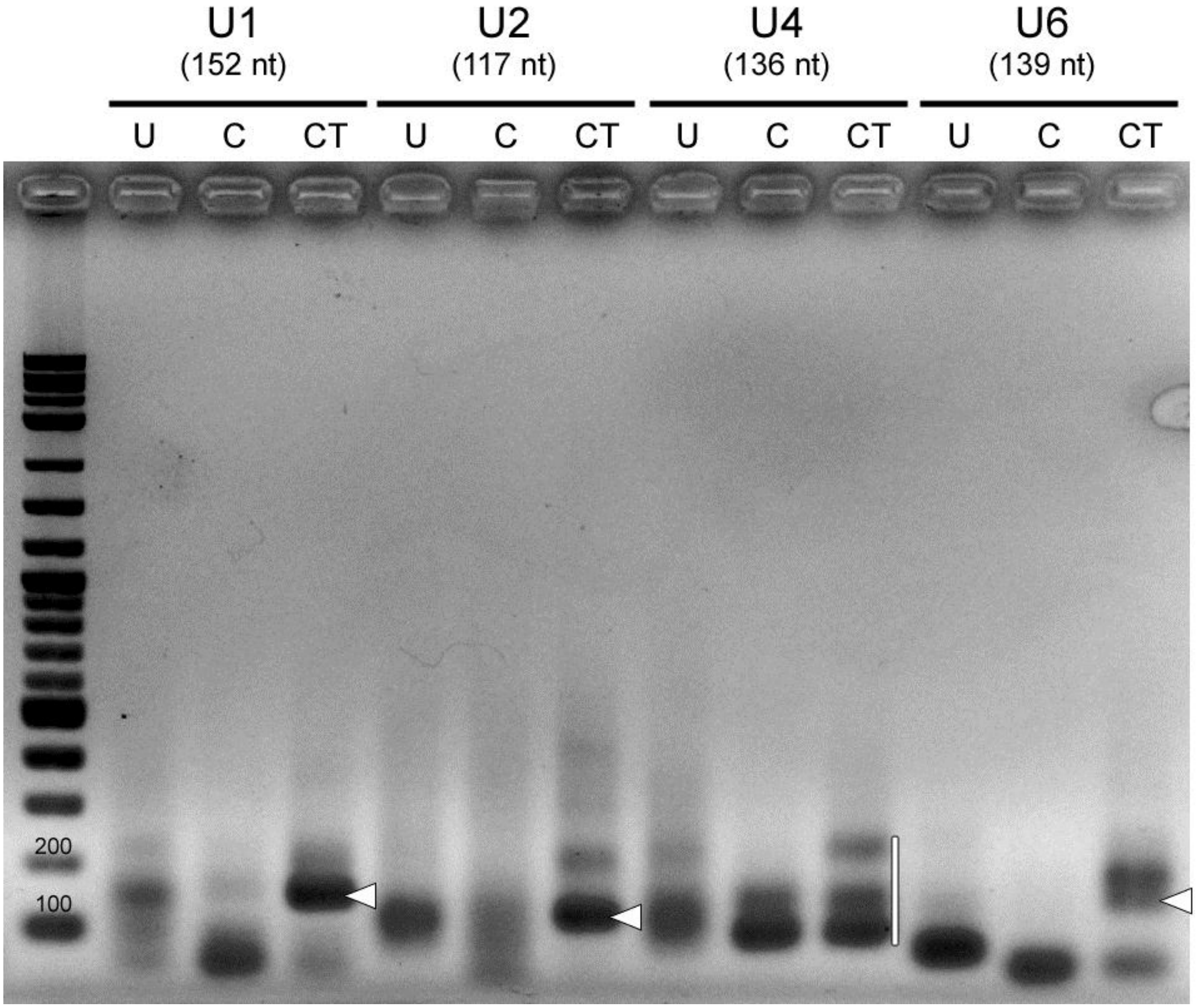

Supplementary Figure 9. RNA linker mediated (RLM) 5' RACE analysis of snRNA candidates
Giardia WB total RNA was untreated (U), CIP-treated (C) or CIP plus TAP-treated (CT) before ligation of a common RNA linker to RNA 5' ends followed by RT-PCR amplification (see methods for more details). Only products indicated with open arrowheads from "CT" treatments were determined to be snRNA products by DNA sequencing following gel extraction of bands, thus indicating the presence of 5' nucleotide cap structures. Other sequences were determined to be non-specific (i.e. non-snRNA) amplification products. Sequences appearing in the "U" and "C" treatments determined they were also non-specific RT-PCR amplicons and did not correspond to uncapped versions of the snRNAs. U4 "CT" treatment products (white bar) were individually gel-extracted, cloned and sequenced which identified several non-specific amplification products and a single U4 sequence clone. Expected sizes for snRNA RT-PCR products and DNA marker bands are given in nucleotides (nt).

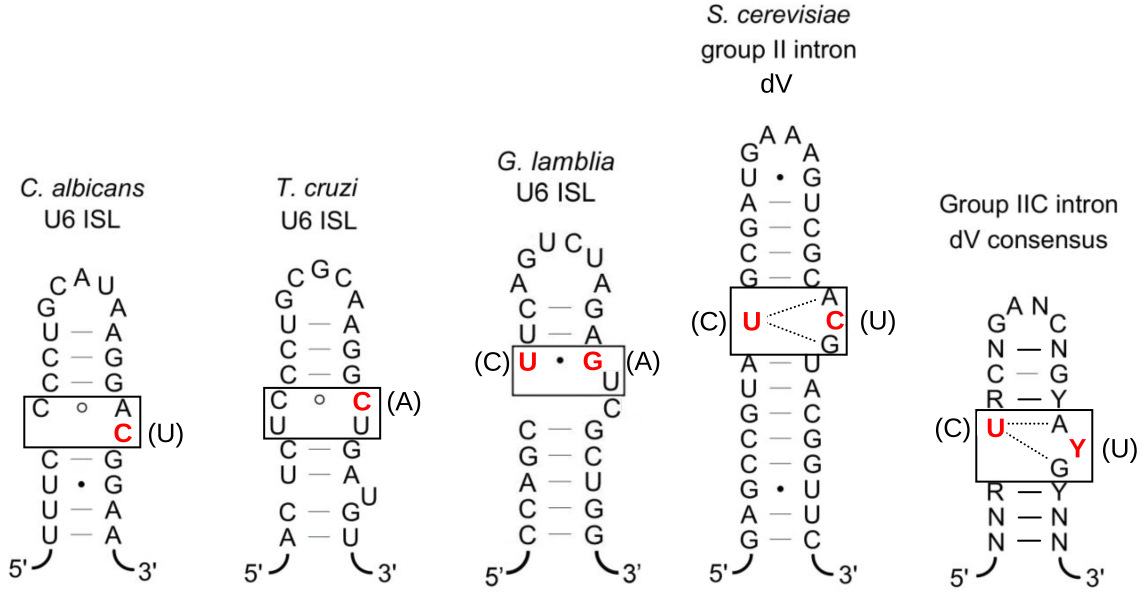

Supplementary Figure 10. Sequence divergence within the internal stem loops (ISL) in U6 snRNAs from diverse eukaryotes compared to domain V of group II introns

Predicted secondary structures for the U6 ISL from the human pathogens *C. albicans* (43), *T. cruzi* (50) and *G. lamblia* (this study). These are compared to domain V (dV) from yeast ai5γ group II intron (16) and the group IIC intron consensus (17). Non-canonical interactions in the predicted Mg^{2+} binding sites are highlighted with boxes where those nucleotides differing from the eukaryotic consensus are indicated in red text, with eukaryotic consensus nucleotides at those positions indicated in brackets.

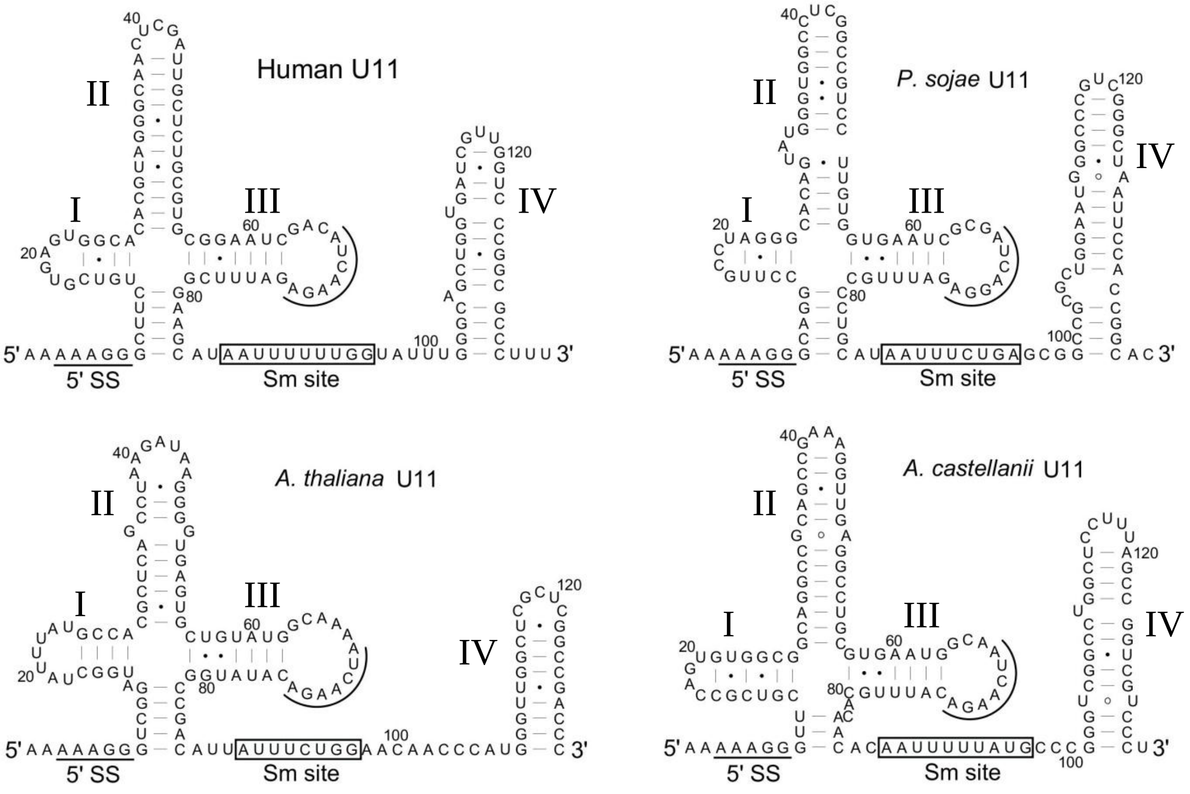

Supplementary Figure 11 – A novel conserved sequence motif in U11 snRNA stem-loop III

Secondary structural predictions of representative eukaryotic U11 snRNAs from humans (66), *Phytophthora sojae* (10), *Arabidopsis thaliana* (66) and *Acanthamoeba castellanii* (11) are shown with their 5’ splice site interacting sequences (5’ SS) underlined and Sm protein binding sites (Sm site) in boxes. We note a novel conserved sequence element of unknown function (underlined loop sequence with consensus ‘AUCARGA’) within stem-loop III (SL III) that is also present at the same relative position within SL III from the novel *Giardia* U1 snRNA.

Supplementary Figure 12 – Primary sequence comparison of *G. lamblia* U2, U4 and U6 snRNA candidates with representative U2- and U12-dependent spliceosomal snRNAs

Full-length *G. lamblia* WB isolate U2, U4 and U6 snRNA primary sequences (this study) were aligned with U2- and U12-dependent spliceosomal snRNAs from diverse eukaryotes using ClustalW 2.0 (29). Regions predicted to form snRNA-snRNA, snRNA-intron or intramolecular base pairings in the *G. lamblia* snRNAs are indicated above the alignments. Letters below the alignments in A-1, A-2 indicate *G. lamblia* snRNA nucleotide positions which are major/U2-dependent ('M') and/or minor/U12-dependent ('m') spliceosomal-like whereas in B and C, they indicate a position exclusively major or minor spliceosomal-like. (**A and B**) Grey boxed nucleotides are conserved in at least four snRNAs. (**C**) Nucleotides which are universally conserved between all U6 and U6atac snRNAs are in red text. "BP" = intron branch point interacting sequence, "SL" = stem-loop. Alignments were constructed using *Giardia lamblia* (Gl) U2 [JX416862], U4 [JX416863] and U6 [JX416864]; *Acanthamoeba castellanii* (Acan_cas) U2 [GenBank CW933695: nucleotide positions 787-579], U12 [CW917526:369-205], U6 [CW934080:725-629] and U6atac [AEYA01001292:233679-233780]; *Phytophthora* spp. (Phy_species) U2 [*Phythophthora ramorum* genome release V1.0 scaffold_1672:234-416], U12 [AAQY02000248:644532-644696], U4 [AATU01001408:348748-348878], U6 [AATU01006594:28185-28081] and U6atac [AATU01001737:24057-24182]; *Arabidopsis thaliana* (Arabi) U2 [X06478:200-359], U12 [CP002684:22603122-22603295], U4 [X67145:194-344], U4atac [CP002687:9096362-9096515], U6 [X52527:306-408] and U6atac [CP002688:16166306-16166185]; and *Homo sapiens* (Human) U2 [NR_002716:1-187], U12 [L43846:331-480], U4 [NR_003137:1-141], U4atac [AC073911.38:96778-96890], U6 [M14486:329-435] and U6atac [NR_023344:1-125] snRNA sequences.

A-1) U2 Alignment

```
                                            U2/U6 hI              U2/U6
                               U2/U6 hII     Ib   Ia         BP       hIII
Acan_cas_U2           -ACATCTT-CTCGGCCCAAGTGGCTAAGATCA-TGTGAAGTATCTGTTCTTATCAGCTTA 57
Phy_ramorum_U2        ---ACCTT-CTCGGCCTTT-TGGCTAAGATCA-AGTGTAGTATCTGTTCTAATCAGTGTG 54
Arabi_U2              -ATACCTTTCTCGGCCTTT-TGGCTAAGATCA-AGTGTAGTATCTGTTCTTATCAGTTTA 57
Human_U2              -ATCGCTT-CTCGGCCTTT-TGGCTAAGATCA-AGTGTAGTATCTGTTCTTATCAGTTTA 56
Gl_U2                 --------TAAAATCAGAGTCGGCTTCGACTTTAGTGTAGTTACTGTT-TCGTCGGCTTA 51
                                    M      MMMM  MM    MMMMMMMM  MMMMM M  MM M MMM
                                                                       Sm site
Acan_cas_U2           ATCTCTGGTAGTGAGGCCTCCTGTGCCTCACCTCAAGGTTAGACTTATTTTTCTTGTTGG 117
Phy_ramorum_U2        AAAACTGGT---TCCGACGTTTTTCGTT--GGTCTTTTTCACATTCATTTTTGG------ 104
Arabi_U2              ATATCTGATATGTGGGCCATCGGC-CCACACGAT---ATTAACTCTATTTTTTAAGGGAG 113
Human_U2              ATATCTGATA--CGTCCTCTATCCGAGGAC--AATATATTAAATGGATTTTTGGA----- 107
Gl_U2                 ACCGCCGAT-------------CCACTACATGCAAGGGGCAGCCGG------GCTGTGAG 92
                      M   M   M                               M

Acan_cas_U2           GC-TCCTGGCACCATGCCCTTCCAGCTATGCTGTGGGCAGTCCAGAGAGCAGTGATC--- 173
Phy_ramorum_U2        GCATCCCGATGTCGCGC------AGCT-TGCTGTGCGAGGTC---GGGGCGGTTTCCGGG 154
Arabi_U2              AAAGCCCGTTAAGAT--------AGCT-TGCTATCTG----------------------- 141
Human_U2              GCAGGGAGATGGAATAGG-----AGCT-TGCTCCGTCCACTC-------CACGCATC--- 151
Gl_U2                 GCAGC-TGCCAGGATG--------GTCCTGCCTTGTC------------------CC--- 123
                      MMM M  M     MM         M   MMM  M                      M

Acan_cas_U2           AGCTTTGTACTGCACCACCCTGCAAAGTTCTTCAAAT- 210
Phy_ramorum_U2        GGCTTT-CACCTCTCC--CCCGCGAGGC----CAAC-- 183
Arabi_U2              GGCTTT--------------CGCGAGTCGCC-CA---- 160
Human_U2              GACCTGGTATTGCAGTACCTCCAGGAACGGTGCACC-- 187
Gl_U2                 GGCT-------------------GGCGCCGTCCACCTT 142
                      MMMM                   M   M    MM
```

A-2) U12 Alignment

```
                                   U2/U6 hI        U2/U6
                                    Ib     Ia        BP     hIII
Human_U12      ------------ATGCCTTAAACTTA-TGAGTAAGGAAAATAACGATTCGGGGTGA------------- 43
Phy_sojae_U12  --------------GGCTTAAACTCAATGAGTAAGGAAACTAACGC-CCTACCTGACAAGTAGGGCTGC 54
Acan_cas_U12   --------------GCCTTAAACT-AATGAGTAAGGAAAATACCGC-ACCGGTTGAC-ACCGGTGTTAT 52
Arabi_U12      --------------GCCTTAAACT-AATGAGTAAGGAAAACAAAGC-GTCCGGTGAGAACCG--GTCGC 51
Gl_U2          TAAAATCAGAGTCGGCTTCGACTTTA---GTGTAGTTACTGTTTCGT--------------------- 44
                             m mmm  mmm  m   mm   m  m m    m

                                                      Sm site
Human_U12         CGCCCGAATCCTC-ACTGCTA--ATGTGAGACGAATTTTTGA-GCGGGTAAA--GGTC-- 95
Phy_sojae_U12     CTGTTGGATCG---AGTGATC-CATGGGTTTCTAATATTTGACGGAGGTGGAATGGCC-- 108
Acan_cas_U12      GGCGTGAACCG-----CGTTCACGTCGGCTTCCAATTTCTGGCG-GGCTACA--GGCCTC 104
Arabi_U12         GGCGCTAATCGTAAAACACAAAATTGGCGCAATAATTTATGGAGGGGTTATA--GGCT-- 107
Gl_U2             CGGCTTAACCGCCGATCCACTACATG------CAAGGGGCAGCCGGGCTGTGA-GGCA-- 95
                   m     mm mm    m         mm       mm           mm m      mmm

Human_U12         -GCCCTCAA--GGTGACC--CGCCTACTTTG---CGGGATGCCT-----GGGAGTTGCGA 142
Phy_sojae_U12     -GTCATTTT--AGGAACC--CG-CTACTTT----TAGGGTGACTAAAAAGGGGGCCGGTC 158
Acan_cas_U12      GGCCCTCTTGTAGTGACCTGCGCCTACTTTCGCGCGGGATGCTCGA---GTTGGCTGGCC 161
Arabi_U12         GGCCGATGT--GTTGACGC-TGCTTACTTTT--GCAGAACTCACCTCGTGCGGGCCTCCC 162
Gl_U2             -GCTGCCAG--GATGGTC-----CTGCCCT---------TGTCCCG---GCTGGC-GCCG 134
                   mm          mm  m      mm m  m         mm         m  mmm m

Human_U12         TCTGCCCG---- 150
Phy_sojae_U12     CCCGCCC----- 165
Acan_cas_U12      TCCG-------- 165
Arabi_U12         TACACCCATCCC 174
Gl_U2             TCCACCTT---- 142
                  m m mm
```

B) U4+U4atac Alignment

```
                       U4/U6 or U4a/U6a hII          U4 5′ Stem-loop
Human_U4atac      --ACCATCCTTCTCGTGGG-GTTGTGTTACTGTCCAGTGAGCGCATGGT-GAGGGCA--A 54
Arabi_U4atac      AACCCGTTTCTGTCAGAGGTGAAGGATGATCCGTCAATGATCGTTTAGA-GACGGCGG-A 58
Human_U4          -----AGCTTTGCGCAGTG-GCAGTATCGTAGCC-AATGAGGTT-TATCCGAGGCGCGAT 52
Arabi_U4          -----ATCTTTGCGCTTGGGGCAATGACGCAGCT-AATGAGGTTCTAACCGAGGCGCGTC 54
Phy_inf_U4        -----ATCTTTGTGCTTGGGGCAATACGATAGTGT-GTGAAGCTCTGCT-GATGCATCGT 53
Gl_U4             -----GACTCTAGGCT-GAAGC--TGCCAAGGTGC-GTGATCCCTCGGT-GATGCCTTGA 50
                          MM      M         M                       M
                                                m      m     m       m   m

                U4/U6 or
                U4a/U6a hI
Human_U4atac    -TACTGCTAACGC----CTACA-C-AACACA---CCCACATCAA---------------CT 90
Arabi_U4atac    -TCGTGCCGACACAGAATTTGA-CGAACATAATTTCAAGGCGAGTGGGCCTTGCCTTACT 117
Human_U4        -TATTGCTAATTGAAAACTTTTCCCAATAC----CCCGCCATGA--CGACTTGAAA----T 102
Arabi_U4        -TATTGCTGGTTGAAAACTATTTCCAA-AC----CCCCTCCT----AGGCCTAAG------ 99
Phy_inf_U4      -GATTGCTAGTTGAAAACTACTCC-AACAC-----CCGTGAGAA---GGCCAC-------- 96
Gl_U4           GTGTTGCTTCACCAAAGAAC----AACCACA------------------------------ 77
                  M          M
                           m

                                 Sm site
Human_U4atac      ATGGTG--GTGC---------AATTTTTTGAAAA-------------------- 113
Arabi_U4atac      TTGGTT--GGGCCTGCCCGTCAATTTTTGGAAGC------CTCGA--------- 154
Human_U4          ATAGTC---GGCAT--TGG-CAATTTTTGACAGT---CTCTACGGAGA------ 141
Arabi_U4          CTTGTCTTAGGCCT--TCGAGAATTTCTGGAAGGGCTCCCTTTGGGGTAAAGCC 151
Phy_inf_U4        -TGGC---CAGCTC------CAATTTCTGTTTTATCTCCCACTAT--------- 131
Gl_U4             -CGGCA--CAGC-------CGAATCTCTCATT---------------------- 99
                                         M
```

C) U6+U6atac Alignment

```
                                                 U6/U2 or U6a/U2a   U6/U2 or U6a/U2a
                                                       hIII                 hI
Human_U6             GTGCTCGCTTCGGCAGCA-CATATACTAAAATTGGAACGATACAGAGAAGATTAGCATGG 59
Arabi_U6             ---GTCCCTTCGG--GGA-CATCCGATAAAATTGGAACGATACAGAGAAGATTAGCATGG 54
Acan_cas_U6          -----------GGAGGCTCCATCTGTTAAAATTGGAACGATACAGAGAAGATTAGCATGG 49
Phy_inf_U6           --GACCACTTCGGTGGT--CATCCGTAAAAATTGGAACGATACAGAGAAGATTAGCATGG 56
Phy_inf_U6atac       ----------------------------GTGTTCGTTGAGCCGAGAGAAGGTTAGCATC- 31
Acan_cas_U6atac      ----------------------------GTGCTGGTTGAGCCGAGAGAAGGTTAGCATC- 31
Human_U6atac         ----------------------------GTGTTGTATGAAAGGAGAGAAGGTTAGCACT- 31
Arabi_U6atac         ----------------------------GTGTTCGTAGAAAGGAGAGATGGTTGGCATC- 31
Gl_U6                ----------------------------GTGGTTAACAAAAACAGAGACAGTTAGCACCA 32
                                                             MM
                                                 mmm       m           m       m

                     U6/U4 or U6a/U4a
                            hII                                   U6atac 3' SL
Human_U6             CCCCTGCGCAAGGATGACA---------CGCA------AA--------TTCGTG--AAGC 94
Arabi_U6             CCCCTGCGCAAGGATGACA---------CGCAT-----AA--------ATCGAG--AAAT 90
Acan_cas_U6          CCCCTGCGCAAGGATGACA---------CGCA------AA--------ATCGAG--AAGA 84
Phy_inf_U6           CCCCTGCGCAAGGATGACA---------CGCAT-----AA--------ATCGAG--AAG- 91
Phy_inf_U6atac       TCCCTCGACAAGGACGGGATTGCGCGTTTGCGTATC-CAAC-CACTGGATGGT-TTAAGC 88
Acan_cas_U6atac      TCCCTGCATAAGGACGGGAAAAGAC-TCCG-GTCTT-CAACTCAC---ATCGTGTTAAGG 85
Human_U6atac         CCCCTTGACAAGGATGGAAGAG-GCCCTCGGGCCTGACAACACGC---ATACGGTTAAGG 87
Arabi_U6atac         TCCTCTGACAGAGACGGGATTTGACCTTCGGGTCTTTGAACC--AC---ATCCGGTTAAGG 86
Gl_U6                GCTTCAGTCTAGAGTCGCTGGGGGACCTCTGGTTTCGCGGG-------AGCCCGTTGGCG 85
                                     M
                           m         m          m   m  m  m                mm   m

                       U6atac 3' SL                 Lsm Site
Human_U6             GTT------------------------CCATATTTTT-- 107
Arabi_U6             GGT------------------------CCAAATTTTT-- 103
Acan_cas_U6          TAC------------------------CCAACTTTTT-- 97
Phy_inf_U6           TAT------------------------CGCACTTTTTG- 105
Phy_inf_U6atac       T-CTGTCATCCTTCTGGAAGACATCTACCAGTTTTTTTT 126
Acan_cas_U6atac      C-TAGTAAC------------------ACTAACTTTT--- 103
Human_U6atac         CATTGCCACCTACTTCGTGGCATCTAACCATCGTTTTT- 125
Arabi_U6atac         C-TCTCCACATTCGTGTGGATCTAAACCCAATTTTTT-- 122
Gl_U6                CGTGCTTGCACCCCGCTCCT------------------- 105

                     m       m
```

Name	Sequence (5'-3')	Description

Supplementary Figure 13 – Oligonucleotide Primers Used in this Study

p-94	AAT AAA GCG GCC GCG GAT CCA ATT TTT TTT TTT TTT TTT V	Reverse primer for 3' RACE of polyA-tailed *Giardia* total RNAs. 'V' indicates any nucleotide except for 'T'.
oAH1	GAT CCT CTT CAT CCC CAA GCG C	Forward primer for RT-PCR used in combination with oAH2 to detect Hsp90 and Replication Factor C dicistronic transcript. Primer sequence corresponds to position +954 to +976 of Hsp90 exon 1.
oAH2	GAT GAG GTG GAT TTT TTG CCC GG	Reverse primer for RT-PCR used in combination with oAH2 to detect Hsp90 and Replication Factor C dicistronic transcript. Complementary to the coding sequence +84 to +106 nt. downstream of Replication Factor C start codon.
oAH9	GGC CGC GAT GCA GCT CGA AGC	Forward primer used for RT-PCR detection of the expression of DHC beta exon 2 and downstream flanking region precursor transcript. Primer sequence spans +3924 to +3944 coding sequence of DHC beta exon 2.
oAH10	GGG CCC CTC TCT TCC TCT CTT CC	Reverse primer used with oAH9 for RT-PCR detection of the expression of DHC beta exon 2 and downstream flanking region precursor transcript. Primer is complementary to the region +197 to +219 nt. downstream of the 5' splice site
oAH13	CGT TTG AAA TGT GCT CCA AGG G	Forward primer used with oAH14 for RT-PCR detection of DHC beta exons 3 and downstream flanking region precursor transcript. Primer anneals in exonic region -82 to -103 nt. upstream of the *trans*-intron 5' splice site.
oAH14	GTT GGC AGA TAG ATT GGT AGG C	Reverse primer used with oAH13 for RT-PCR detection of DHC beta exons 3 and downstream flanking region precursor transcript. Primer is complementary to +236 to +257 nt. downstream of the *trans*-intron 5' splice site.
oAH32	GTT ATT ACC CTC ATC CCC TCT TGC	Forward primer used with oAH33 for RT-PCR detection of DHC gamma exon 1 and Hypothetical Protein dicistronic transcript. Primer anneals within exonic region -88 to -111 nt. upstream of the *trans*-intron 5' splice site.
oAH33	GAG CTG ACC TGG ACA TAA AGA GC	Reverse primer used with oAH32 for RT-PCR detection of DHC gamma exon 1 and

Name	Sequence (5’-3’)	Description
		Hypothetical Protein dicistronic transcript. Sequence is complementary to +24 to +46 nt. downstream of the Hypothetical Protein start codon.
oAH60	GGC CGG CAT AAC CGA AAT CG	Reverse primer for primer extension and 5’ RACE of *G. lamblia* **GlsR26**. Primer anneals -9 to -28 nt. upstream of 3’ processing motif sequence.
oAH62	GAG ATC ACA AAT GTG CTC CGG CCA GG	Reverse primer for primer extension and 5’ RACE of *G. lamblia* **GlsR27**. Primer anneals from -6 to -31 nt. upstream of 3’ processing motif sequence.
oAH70	GGA TGG GAT CCT TCC CCT TGC TTC TGG	Reverse primer for primer extension and 5’ RACE of *G. lamblia* **GlsR28**. Primer anneals from -8 to -34 nt. upstream of 3’ processing motif sequence.
oAH72	GGT GCA AGC ACG CGC CAA CGG GC	Reverse primer for primer extension and 5’ RACE of *G. lamblia* **U6 snRNA candidate**. Primer anneals from -6 to -28 nt. upstream of 3’ processing motif sequence.
oAH73	CGG GCC CGG ATT GAG GAT GGA CG	Reverse primer for RT-PCR detection of GlsR17 + GlsR18 polycistronic transcript precursor. Anneals within GlsR18 mature sequence, -7 to -29 nt. upstream of the GlsR18 3’ processing motif.
oAH74	ATA ATG CGC TTC TTT GAG CCG CGG G	Forward primer to be used with oAH73 for RT-PCR detection of GlsR17 + GlsR18 polycistronic transcript precursor. Anneals within the mature GlsR17 sequence, -101 to -125 nt. upstream of the GlsR17 3’ processing motif.
oAH75	GAG GCT GCT AAA ACA CAG GGC	Forward primer to be used with oAH60 for RT-PCR detection of GlsR25 + GlsR26 polycistronic transcript precursor. Anneals within GlsR25 mature sequence, -94 to -115 nt. upstream of the GlsR25 3’ processing motif.
oAH76	ATA AGC TGG AAT TCC ACG TCT TCC TCG	Reverse primer to be used with oAH77 for RT-PCR detection of Candidate-23 + DNA polymerase delta catalytic subunit polycistronic transcript. Primer is antisense to region +54 to +80 downstream of predicted first “AUG” codon of DNA pol mRNA (protein CDS in region is conserved with *Entamoeba* and *Culex Spp.*)

Name	Sequence (5'-3')	Description
oAH77	GGC ATG GAG AAG AGC AGA CTT GAG G	Forward primer to be used with oAH76 for RT-PCR detection of Candidate-23 + DNA polymerase delta subunit polycistronic transcript. Anneals within Candidate-23 mature sequence to region -7 to -31 upstream of 3' processing motif.
oAH78	GGA GCT CGA CCA TTT TCA CAT CCC	Reverse primer for use with oAH79 for RT-PCR mediated verification of Candidate-5 + Ser/Thr kinase CDS polycistronic transcript. Is complementary to +146 to +169 downstream of predicted first AUG codon of Ser/Thr mRNA. Anneals in portion of CDS which encodes an ankyrin domain.
oAH79	CCT TGC CCA GTC TGC CTC CAT AC	Forward primer to be used with oAH78 for RT-PCR amplification of Candidate-5 + Ser/Thr kinase polycistronic transcript. Anneals within Candidate-5 sequence -9 to -31 upstream of its 3' processing motif.
oAH95	GGG TAC GGT AGC AGG TCT GAG AGC	Reverse primer for primer extension and 5' RACE of *G. lamblia* U1 snRNA candidate. Anneals to region -12 to -35 upstream of the 3' motif sequence.
oAH102	GTT ATG TTT GTA TGC TGT ATG TGT GC	Forward primer for 3' RACE of *Giardia* HSP90 *trans* intron 5' half. Primer anneals to region +5 to +31 downstream of exon 1-intron splice site and upstream of the 3' motif sequence.
oAH103	GTA TGT TAC TGG GTG AAA CGC TAC	Forward primer for 3' RACE of *Giardia* DHC Beta *trans* intron #1 - 5' half. Primer anneals to region +1 to +24 downstream of exon 2 - intron 1 splice site and upstream of the 3' motif sequence.
oAH104	GTA ATC TGT GTA GTC GCA GTA TGC C	Forward primer for 3' RACE of *Giardia* DHC Beta *trans* intron #2 - 5' half. Primer anneals to region +9 to +33 downstream of exon 3 - intron 2 splice site and upstream of the 3' motif sequence.
oAH105	CAC AGG TGG TTT GGT GTG TAT GC	Forward primer for 3' RACE of *Giardia* DHC Gamma *trans* intron 5' half. Primer anneals to region +8 to +30 downstream of exon 1 - intron splice site and upstream of the 3' motif sequence.
oAH112	CAC TCA AGT ATG TTC TTG CG	Forward primer for 3' RACE of Protein 21.1 CDS (GL50803_25296) to detect processing near its 3' motif sequence. Primer anneals within protein coding region -13 to -32 nt. upstream of the predicted 3' motif sequence.

Name	Sequence (5'-3')	Description
oAH113	AGC TTT CTA AAA CCA CTC CC	Forward primer for 3' RACE of Hypothetical Protein CDS (GL50803_7350) for detection of processing near its 3' motif sequence. Primer anneals within protein coding region -20 to -39 nt. upstream of the predicted 3' motif sequence.
oAH114	CTG TAT TGT ATG CTT CAA TGG	Forward primer for 3' RACE of U5 Helicase Protein CDS (GL50803_9352) for detection of processing near its 3' motif sequence. Primer anneals within protein coding region -37 to -47 nt. upstream of the predicted 3' motif sequence.
oAH117	CGT GTG GTT GTT CTT TGG TG	Reverse primer for 5' RACE of *G. lamblia* U4 snRNA candidate (Candidate-11). Primer is antisense to region -17 to -36 upstream of 3' motif sequence.
oAH118	CAC CAA AGA ACA ACC ACA CG	Forward primer for 3' RACE of *G. lamblia* U4 snRNA candidate (Candidate-11). Primer corresponds to -17 to -36 upstream of 3' motif sequence.
oAH119	CCT GGC AGC TGC CTC ACA GC	Reverse primer for 5' RACE of *G. lamblia* U2 snRNA candidate (Candidate-14). Primer is antisense to region -35 to -54 upstream of 3' motif sequence.
oAH120	GCT GTG AGG CAG CTG CCA GG	Forward primer for 3' RACE of *G. lamblia* U2 snRNA candidate (Candidate-14). Primer corresponds to region -35 to -54 upstream of 3' motif sequence.
oAH123	GCT GTA ATA CGA CTC ACT ATA GGC TAG GCT GAA GCT GCC AAG GTG CG	Forward primer for *G. lamblia* U4 snRNA (Candidate-11) *in vitro* transcription and to generate probe for Northern blot (use with oAH 124). Primer anneals to region +1 to +24 nt from predicted mature 5' end. A T7 promoter sequence is underlined.
oAH124	AAT GAG AGA TTC GGC TGT GCC	Reverse primer for *G. lamblia* U4 snRNA (Candidate-11) *in vitro* transcription and to generate probe for Northern blot (use with oAH123). Primer is antisense to region -21 to -1 nt. from mature 3' end.
oAH125	GCT GTA ATA CGA CTC ACT ATA GGT AAC AAA AAC AGA GAC AGT TAG CAC C	Forward primer for *G. lamblia* U6 snRNA candidate *in vitro* transcription and to generate probe for Northern blot (use with oAH126). Primer anneals to region +1 to +26 nt from predicted mature 5' end. A T7 promoter sequence is underlined.
oAH126	AAG GAG CGG GGT GCA AGC ACG	Reverse primer for *G. lamblia* U6 snRNA

Name	Sequence (5'-3')	Description
		candidate *in vitro* transcription and to generate probe for Northern blot (use with oAH125). Primer is antisense to region -20 to -1 nt. from mature 3' end.
oAH127	GCT GTA ATA CGA CTC ACT ATA GGC AGA GTC GGC TTC GAC TTT AGT GTA G	Forward primer for use with oAH128 to generate PCR probe for U2 snRNA (Candidate-14) Northern blot. Primer anneals to region +1 to +26 nt from predicted mature 5' end. A T7 promoter sequence is underlined.
oAH128	AAG GTG GAC GGC GCC AGC C	Reverse primer for use with oAH127 to generate PCR probe for U2 snRNA candidate (Candidate-14) Northern blot. Primer is antisense to region -19 to -1 nt. from predicted 3' end.
oAH129	GCT GTA ATA CGA CTC ACT ATA GGT TTT AAC TTA CCT CAA GGG TGG CG	Forward primer for use with oAH130 to generate probe for U1 snRNA Northern blot. Primer anneals to region +1 to +24 nt from predicted mature 5' end. A T7 promoter sequence is underlined.
oAH130	AAG GAA AAA TTA AAA GGG TAC GGT AGC	Reverse primer for use with oAH129 to generate probe for U1 snRNA Northern blot. Primer is antisense to region -27 to -1 nt. from predicted 3' end.
oAH133	CAG TGC TCT CAG ACC TGC TAC C	Forward primer for 3' RACE of U1 snRNA candidate. Primer anneals to region -39 to -17 upstream of 3' motif sequence.
oAH136	TCT CTG TTT TTG TTA CC	Giardia U6 snRNA candidate antisense primer to be used during *in vitro* U4/U6 complex formation. Is complementary to region +1 to +17 from mature 5' end.
oAH137	GAA ACC AGA GGT CCC CCA GC	Giardia U6 snRNA candidate antisense primer to be used during *in vitro* U4/U6 complex formation. Is complementary to region +46 to +65 from mature 5' end.
oAH139	GGC GAT TCG GCT GTG CCG TGT GG	Reverse primer for 5' RACE of *G. lamblia* U4 snRNA candidate (Candidate-11). Primer is antisense to region -4 to -23 upstream of 3' end motif sequence.
oAH140	GGC CCT TGC ATG TAG TGG ATC GG	Reverse primer for 5' RACE of *G. lamblia* U2 snRNA candidate (Candidate-14). Primer anneals to region -63 to -83 nt. upstream of 3' end motif sequence.
oAH141	GGG CTT TGA CAG AGA CCA CTG CGA G	Reverse primer for 5' RACE of GlsR28. Primer anneals to region -51 to -72 nt. upstream of 3' motif sequence.
oDE1	TTC TGA TGC GGA TAC CTT GC	Forward primer for the 3'-RACE of Candidate-

**Name**	**Sequence (5'-3')**	**Description**
		17. Primer anneals -102 to -82 relative to the 3' processing motif.
oDE3	CTT GCG TGC GCA TAT CTC C	Forward primer for the 3'-RACE of GlsR26. Primer anneals -73 to -54 relative to the 3' processing motif.
oDE4	CTA CGT GTT ATG GGC AGC G	Forward primer for the 3'-RACE of GlsR27. Primer anneals -82 to -63 relative to the 3' processing motif.
oDE5	TTC AAC TCA GCC GGA CAG C	Forward primer for the 3'-RACE of Candidate-15. Primer anneals -87 to -68 relative to the 3' processing motif.
oDE7	TAG GTA GGG CCG ATG AGC	Forward primer for the 3'-RACE of Candidate-23. Primer anneals -86 to -68 relative to the 3' processing motif.
oDE8	ACG AGG AAA CGA GTG TTT CG	Forward primer for the 3'-RACE of Candidate-5. Primer anneals -76 to -56 relative to the 3' processing motif.
oDE9	GTA GTT ACT GTT TCG TCG GC	Forward primer for the 3'-RACE of U2 snRNA candidate (Candidate-14). Primer anneals -110 to -90 relative to the 3' end processing motif.
oDE17	CTA TCC AGC CAA GAG CCG	Forward primer for RT-PCR and 3' RACE of processing motif-containing CDS for DNA repair protein Rhp26p (GL50803_87205) transcript. Primer anneals +1005 to +1023 downstream of the predicted start codon and upstream of the predicted motif sequence.
oDE18	GGC GTC GTA GTA TGT AAG G	Reverse primer for RT-PCR detection of motif-containing CDS for DNA repair protein Rhp26p (GL50803_87205) transcript. Primer anneals +138 to +157 downstream of the predicted 3' processing motif.
oDE19	GAT GCC TTA GAG AAC TTG CG	Forward primer for RT-PCR and 3' RACE of motif-containing CDS for U5 200kDa Helicase (GL50803_9352) transcript. Primer anneals +2566 to +2586 downstream of the 'AUG' start codon and upstream of the predicted 3' end motif sequence.
oDE20	CGT AGA TGA GAT AGC TGC C	Reverse primer for RT-PCR detection of motif-containing CDS for U5 200kDa Helicase (GL50803_9352) transcript. Primer anneals +146 to +165 downstream of the predicted 3' end processing motif.
oDE21	ATG GGT CTG ACA CGT TGG	Forward primer for RT-PCR and 3' RACE of process motif-containing Protein 21.1 (GL50803_25296) transcript. Primer anneals to region +538 downstream to +556 of the 'AUG'

Name	Sequence (5'-3')	Description
		start codon and upstream of the predicted 3' end processing motif.
oDE22	TAG ATG AAT CAG CGA TGT GC	Reverse primer for RT-PCR detection of process motif-containing Protein 21.1 (GL50803_25296) transcript. Primer anneals to region +991 to +1011 downstream of the 'AUG' start codon and downstream of the predicted 3' processing motif.
oDE23	GTG ACC GTC CTC TGT ACG	Forward primer for RT-PCR and 3' RACE of process motif-containing hypothetical protein (GL50803_7350) transcript. Primer anneals to region +3156 to +3174 downstream of the 'AUG' start codon and upstream of the predicted 3' motif sequence.
oDE24	AAG ATG AAG GGC TCA AAA CG	Reverse primer for RT-PCR detection of process motif-containing Hypothetical Protein (GL50803_7350) transcript. Primer anneals to region +3618 to +3638 downstream of the 'AUG' start codon and downstream of the predicted 3' end motif.

Supplemental References

10. Russell, A.G., Charette, J.M., Spencer, D.F. and Gray, M.W. (2006) An early evolutionary origin for the minor spliceosome. *Nature*, **443**, 863-866.

11. Lopez, M.D., Rosenblad, M.A. and Samuelsson, T. (2008) Computational screen for spliceosomal RNA genes aids in defining the phylogenetic distribution of major and minor spliceosomal components. *Nucleic Acids Res.*, **36**, 3001-3010.

16. Zhang, L. and Doudna, J.A. (2002) Structural insights into group II intron catalysis and branch-site selection. *Science*, **295**, 2084-2088.

17. Michel, F., Costa, M. and Westhof, E. (2009) The ribozyme core of group II introns: a structure in want of partners. *Trends Biochem. Sci.*, **34**, 189-199.

24. Chen, X.S., White, W.T.J., Collins, L.J. and Penny, D. (2008) Computational identification of four spliceosomal snRNAs from the deep-branching eukaryote *Giardia intestinalis*. *PLoS One*, **3**.

25. Yang, C.Y., Zhou, H., Luo, J. and Qu, L.H. (2005) Identification of 20 snoRNA-like RNAs from the primitive eukaryote, *Giardia lamblia*. *Biochem. Biophys. Res. Commun.*, **328**, 1224-1231.

26. Luo, J., Zhou, H., Chen, C.J., Li, Y., Chen, Y.Q. and Qu, L.H. (2006) Identification and evolutionary implication of four novel box H/ACA snoRNAs from *Giardia lamblia*. *Chin. Sci. Bull.*, **51**, 2451-2456.

27. Chen, X.W., Rozhdestvensky, T.S., Collins, L.J., Schmitz, J. and Penny, D. (2007) Combined experimental and computational approach to identify non-protein-coding RNAs in the deep-branching eukaryote *Giardia intestinalis*. *Nucleic Acids Res.*, **35**, 4619-4628.

28. Chen, X.W.S., Penny, D. and Collins, L.J. (2011) Characterization of RNase MRP RNA and novel snoRNAs from *Giardia intestinalis* and *Trichomonas vaginalis*. *BMC Genomics*, **12**.

29. Larkin, M.A., Blackshields, G., Brown, N.P., Chenna, R., McGettigan, P.A., McWilliam, H., Valentin, F., Wallace, I.M., Wilm, A., Lopez, R. *et al.* (2007) Clustal W and clustal X version 2.0. *Bioinformatics*, **23**, 2947-2948.

31. Zuker, M. (2003) Mfold web server for nucleic acid folding and hybridization prediction. *Nucleic Acids Res.*, **31**, 3406-3415.

43. Mitrovich, Q.M. and Guthrie, C. (2007) Evolution of small nuclear RNAs in *S. cerevisiae*, *C. albicans*, and other hemiascomycetous yeasts. *RNA*, **13**, 2066-2080.

50. Ambrosio, D.L., Silva, M.T.A. and Cicarelli, R.M.B. (2007) Cloning and molecular characterization of *Trypanosoma cruzi* U2, U4, U5, and U6 small nuclear RNAs. *Memorias Do Instituto Oswaldo Cruz*, **102**, 97-105.

66. Schneider, C., Will, C.L., Brosius, J., Frilander, M.J. and Luhrmann, R. (2004) Identification of an evolutionarily divergent U11 small nuclear ribonucleoprotein particle in Drosophila. *Proc. Natl. Acad. Sci. U. S. A.*, **101**, 9584-9589.
